# Metformin promotes histone deacetylation of optineurin and suppresses tumour growth through autophagy inhibition in ocular melanoma

**DOI:** 10.1002/ctm2.660

**Published:** 2022-01-24

**Authors:** Ai Zhuang, Peiwei Chai, Shaoyun Wang, Sipeng Zuo, Jie Yu, Shichong Jia, Shengfang Ge, Renbing Jia, Yixiong Zhou, Wodong Shi, Xiaofang Xu, Jing Ruan, Xianqun Fan

**Affiliations:** ^1^ Department of Ophthalmology Ninth People's Hospital Shanghai Jiao Tong University School of Medicine Shanghai China; ^2^ Shanghai Key Laboratory of Orbital Diseases and Ocular Oncology Shanghai China

**Keywords:** autophagy, histone modification, metformin, ocular melanoma, OPTN

## Abstract

**Objective:**

To explore the therapeutic potential and the underlying mechanism of metformin, an adenosine monophosphate‐activated kinase (AMPK) activator, in ocular melanoma.

**Methods:**

CCK8, transwell, and colony formation assays were performed to detect the proliferation and migration ability of ocular melanoma cells. A mouse orthotopic xenograft model was built to detect ocular tumor growth in vivo. Western blot, immunofluorescence, and electron microscopy were adopted to evaluate the autophagy levels of ocular melanoma cells, and high‐throughput proteomics and CUT & Tag assays were performed to analyze the candidate for autophagy alteration.

**Results:**

Here, we revealed for the first time that a relatively low dose of metformin induced significant tumorspecific inhibition of the proliferation and migration of ocular melanoma cells both in vitro and in vivo. Intriguingly, we found that metformin significantly attenuated autophagic influx in ocular melanoma cells. Through high‐throughput proteomics analysis, we revealed that optineurin (OPTN), which is a key candidate for autophagosome formation and maturation, was significantly downregulated after metformin treatment. Moreover, excessive OPTN expression was associated with an unfavorable prognosis of patients. Most importantly, we found that a histone deacetylase, SIRT1, was significantly upregulated after AMPK activation, resulting in histone deacetylation in the OPTN promoter.

**Conclusions:**

Overall, we revealed for the first time that metformin significantly inhibited the progression of ocular melanoma, and verified that metformin acted as an autophagy inhibitor through histone deacetylation of OPTN. This study provides novel insights into metformin ‐ guided suppression of ocular melanoma and the potential mechanism underlying the dual role of metformin in autophagy regulation.

## Graphical abstract

1. Metformin induces tumor‐specific inhibition in ocular melanomas both in vitro and in vivo.
2. Metformin attenuates autophagic flux in ocular melanoma cells.
3. Metformin epigenetically silenced OPTN through histone deacetylation in the OPTN promoter.

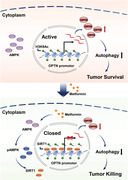

## 1 INTRODUCTION

The antihyperglycemic agent metformin, a biguanide, is the front‐line therapy for patients with type 2 diabetes mellitus (T2DM).^1^ Metformin activates adenosine monophosphate‐activated kinase (AMPK), which leads to gluconeogenesis inhibition in the liver, attenuating insulin resistance and glucose levels and increasing glucose uptake in skeletal muscle.^2^, ^3^ Recently, as epidemiologic data supported metformin may reduce cancer risk in diabetics, metformin has drawn attention in both cancer prevention and therapy.^4^, ^5^ Repurposing metformin as a cancer treatment is being tested in a wide range of clinical trials for a variety of cancers, including renal cell carcinoma, head and neck carcinomas and pancreatic carcinoma.^3^, ^6^, ^7^ In addition, combining metformin with chemotherapies has been attempted in various cancers.^1^, ^8^, ^9^ Although the mechanisms of action of metformin in cancer are not clearly understood, it is known that the energy metabolism of cancer cells differs from that of normal cells, which makes tumourigenic cells vulnerable to metabolism‐interfering drugs.^10^, ^11^

The activation of AMPK by metformin suggests that the improvement in intracellular metabolic profiles could be related to autophagic induction.^5^ Metformin upregulates autophagic activity through direct phosphorylation of UNC51‐like kinase 1 and Beclin1, which are key factors involved in the initiation of autophagy.^12^ In addition, the nicotinamide adenine dinucleotide‐dependent deacetylase Sirtuin 1 (SIRT1) was found to be significantly upregulated after AMPK phosphorylation, inducing autophagy upon glucose starvation.^5^ Although most studies have shown that metformin activates autophagic flux, metformin can also downregulate autophagy and alleviate hyperglycemia‐induced endothelial impairment in a Hedgehog pathway‐dependent manner.^13^ In addition, metformin sensitizes lung cancer cells to osimertinib by inhibiting autophagy.^11^ Thus, metformin plays a dual role in autophagy induction, but further investigations are required to determine their relationships.

The activation of AMPK by metformin suggests that the improvement in intracellular metabolic profiles could be related to autophagic induction.^5^ Metformin upregulates autophagic activity through direct phosphorylation of UNC51‐like kinase 1 and Beclin1, which are key factors involved in the initiation of autophagy.^12^ In addition, the nicotinamide adenine dinucleotide‐dependent deacetylase SIRT1 was found to be significantly upregulated after AMPK phosphorylation, inducing autophagy upon glucose starvation.^5^ Although most studies have shown that metformin activates autophagic flux, metformin can also downregulate autophagy and alleviate hyperglycemia‐induced endothelial impairment in a Hedgehog pathway‐dependent manner.^13^ In addition, metformin sensitizes lung cancer cells to osimertinib by inhibiting autophagy.^11^ Thus, metformin plays a dual role in autophagy induction, but further investigations are required to determine their relationships.

Ocular melanoma, consisting of uveal melanoma (UM) and conjunctival melanoma (CM), is the deadliest malignancy of the eye.^14^ UM is the most common primary malignancy of the eye in adults and is also resistant to the most common chemotherapy and radiotherapy.^15^, ^16^ Half of UM patients develop metastasis, mostly in the liver.^17^ If metastasis occurs, the average overall survival is less than 1 year.^18^ Several oncogenic factors have been found in UM, such as GNAQ/11 mutation, YAP/TAZ pathway activation, BAP1 deletion and chromosomal anomalies.^17^, ^19^ In addition, CM is a rare but sight‐ and life‐threatening malignancy that shows a distinct pattern of gene expression compared to uveal and cutaneous melanoma.^20^ The BRAF V600E mutation, aberrant expression of microRNAs and dysregulated m6A RNA modifications, has been identified.^21^ However, the effect of most therapies on these genetic deficiencies remains less than ideal in both UM and CM, which requires further investigation. Although metformin has been shown to induce tumour‐specific inhibition in different cancers, the role of metformin in ocular melanoma remains unclear.

Thus, we aimed to identify the therapeutic effect of metformin. Herein, we revealed that metformin induced significant tumour‐specific inhibition of the proliferation and migration of ocular melanoma cells both in vitro and in vivo. Intriguingly, we found that metformin significantly attenuated autophagic flux in ocular melanoma cells by epigenetically silencing optineurin (OPTN). These studies provide novel insights into metformin‐guided tumour suppression of malignant ocular melanoma and the potential mechanism underlying the dual role of metformin in autophagy regulation.

## 2 RESULTS

### 2.1 Metformin induces tumour‐specific inhibition in ocular melanomas both in vitro and in vivo

**FIGURE 1:**
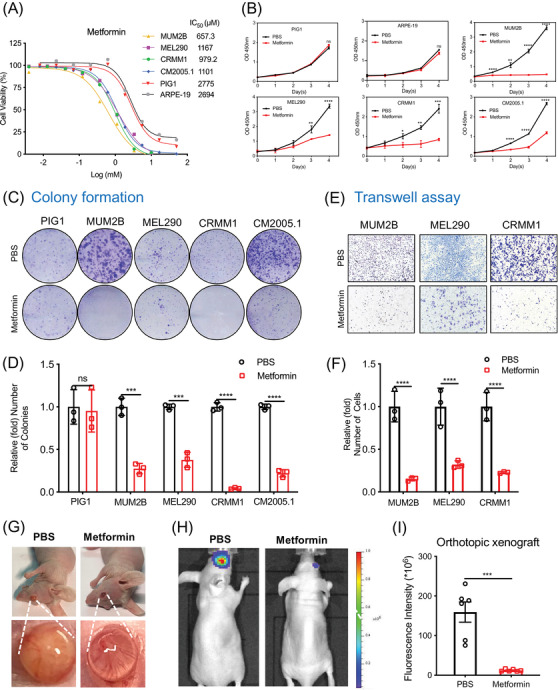
Metformin inhibits ocular melanoma growth in vitro and in vivo. (A) Metformin was tested for its effects on the viability of ocular melanoma cells (MUM2B, MEL290, CRMM1, CM2005.1) and normal control cells (PIG1, ARPE‐19). The IC_50_ values are listed after the cell names. (B) CCK‐8 assay showed the cell proliferation ability after treatment with 1.0 mM metformin. The absorbance at 450 nm was recorded and is presented as the mean ± SD. *p < .05, **p < .01, ***p < .001, ****p < .0001. (C and D) A plate colony formation assay was used to assess the cell proliferation ability after treatment with metformin (1.0 mM, 24 h). Statistical results of three individual experiments are shown as the mean ± SD. ***p < .001, ****p < .0001. (E and F) Transwell assays were performed to assess the cell migration ability after treatment with metformin (1.0 mM, 24 h). All experiments were performed in triplicate, and the statistical results are shown as the mean ± SD. ****p < .0001. (G) Overall and eyeball appearances showed the suppressive effects of metformin on tumour volume in an orthotopic xenograft model. The cells were treated with PBS or metformin (1.0 mM, 48 h) before intraocular injection. (H and I) The animal imaging system demonstrated the suppressive effects of metformin on tumour bioluminescent signals in orthotopic xenografts. Data were presented as the mean ± SEM. ***p < .001

To determine the efficacy of different concentrations of metformin in normal control pigment cells and ocular melanoma cells, we first tested the IC_50_ of metformin in these cells. Notably, the IC_50_ in ocular melanoma cells (MUM2B, MEL290, CRMM1 and CM2005.1) was approximately 1000 μM; however, the IC_50_ in normal pigmented cells was over 2600 μM (Figure 1A). Moreover, we found that 1000 μM (1 mM) metformin did not influence the proliferation of normal control cells, while that of most ocular melanoma cells was significantly inhibited (Figure 1B). In addition, metformin‐treated ocular melanoma cells formed smaller and fewer colonies, while normal control cells remained unaffected (Figure 1C,D). Moreover, 1 mM metformin significantly attenuated the migration of ocular melanoma cells, as shown by the Transwell assay (Figure 1E,F). Importantly, metformin (1.0 mM for 24 h) also triggered significant promotion of apoptosis in ocular melanoma cells (Figure S1A,B). To verify the role of metformin‐induced ocular melanoma inhibition in vivo, we then established an orthotopic UM xenograft model with a luciferase tag (Figure 1G). Animal imaging showed that metformin‐treated MUM2B cells presented with a significantly decreased intensity of bioluminescence (Figure 1H,I).

### 2.2 Metformin attenuates autophagic flux in ocular melanoma cells

**FIGURE 2:**
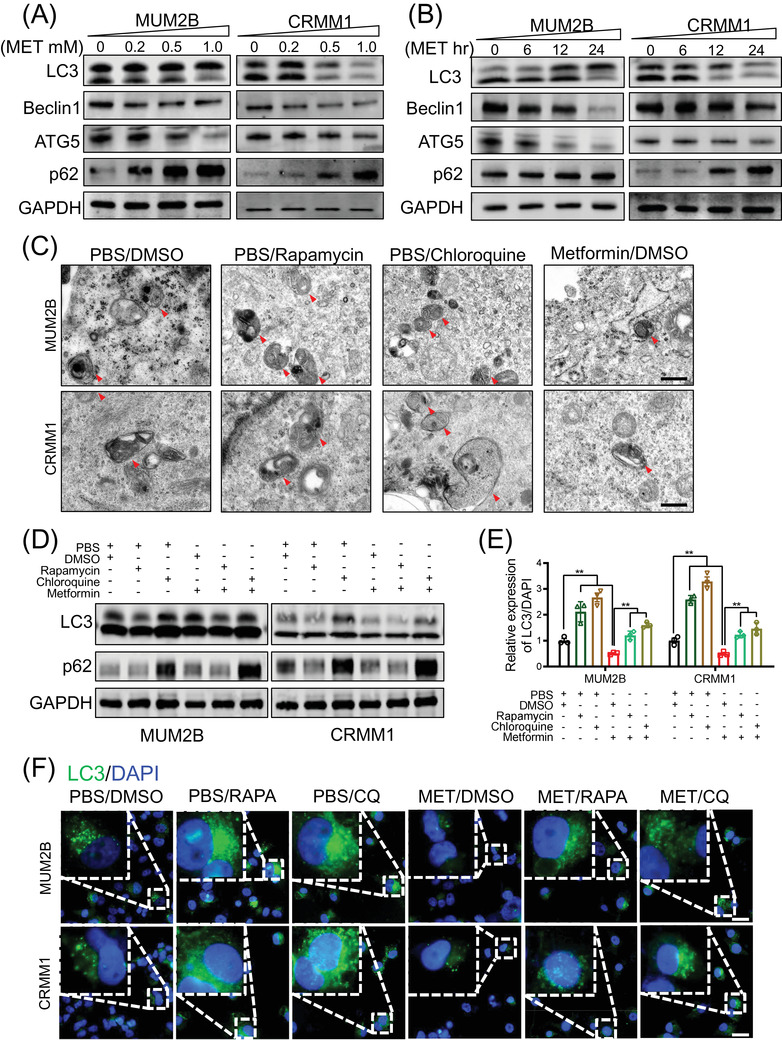
Metformin suppresses autophagy in ocular melanoma. (A) Western blot analysis showed the protein levels of autophagic flux‐related markers (LC3II/I, Beclin1, ATG5, p62) in ocular melanoma cells (MUM2B, CRMM1) treated with a gradient concentration of metformin for 24 h. GAPDH was used as the internal control. (B) Western blot analysis of the protein levels of autophagic flux‐related markers (LC3II/I, Beclin1, ATG5, p62) in ocular melanoma cells (MUM2B, CRMM1) after treatment with 1.0‐mM metformin for different durations. GAPDH was used as a loading control. (C) TEM was used to observe autophagosomes in MUM2B and CRMM1 cells treated with rapamycin (5 μM, 12 h), chloroquine (10 μM, 12 h) and metformin (1.0 mM, 24 h). Representative images are displayed and red arrows indicate autophagosome. Scale bar, 500 nm. (D) Western blot analysis of the protein levels of autophagic markers (LC3II/I, p62) in ocular melanoma cells (MUM2B, CRMM1) treated with metformin (1.0 mM) in the presence/absence of 5 μM rapamycin and 10 μM chloroquine. (E and F) IF was carried out to detect the accumulation of LC3 puncta in MUM2B and CRMM1 cells treated with metformin (MET, 1.0 mM, 24 h) in the presence/absence of rapamycin (RAPA, 5 μM, 12 h) and chloroquine (CQ, 10 μM, 12 h). PBS was used as a control for MET, and DMSO as a control for RAPA or CQ. LC3 expression is presented as the mean ± SEM (E), and representative images are displayed (F). Scale bar: 20 μm. *p < .05, **p < .01

As metformin plays an important part in autophagy regulation, we tested autophagy flux after metformin treatment in ocular melanoma cells. Unexpectedly, metformin‐treated melanoma cells presented decreased levels of LC3 II/I ratio, Beclin1 and ATG5, and increased p62 in a dose‐dependent (Figure 2A and Figure S2A) and time‐dependent (Figure 2B and Figure S2B) manner, which indicated that metformin acted as an autophagy inhibitor in ocular melanoma cells. Furthermore, we investigated autophagosome biogenesis using rapamycin as an autophagy activator and chloroquine as an autophagy inhibitor. Electron microscopy revealed that the number of autophagosomes in ocular melanoma cells treated with metformin was decreased compared to that in untreated cells. In contrast, rapamycin‐/chloroquine‐treated ocular melanoma cells presented with increased autophagosomes (Figure 2C). Western blot analyses demonstrated that LC3 II/I ratio increased and p62 decreased in rapamycin‐treated cells, whereas LC3 II/I ratio and p62 both increased after chloroquine treatment. Most importantly, decreased LC3 II/I ratio and increased p62 level were observed after metformin treatment (Figure 2D). Consistently, GFP‐LC3 puncta was decreased in metformin‐treated cells, which indicated metformin inhibited early autophagy (Figure 2E,F). In addition, tandem mRFP‐GFP fluorescence microscopy confirmed that metformin functioned as a suppressor of autophagic flux, with decreased number of autophagosomes (early) and autolysosomes (late) (Figure S3A–C). The transcription level of p62 was unchanged, which indicated that the autophagic degradation process was attenuated (Figure S2C). Together, these results indicate that metformin significantly attenuates autophagic flux in ocular melanomas. Notably, we found that autophagy was significantly activated along with an increased LC3 II/I ratio and degradation of p62 after metformin treatment in cervical cancer (HeLa), prostate cancer (PC‐3) and breast cancer (MDA‐MB‐231) cells (Figure S4), which is in accordance with other studies.

### 2.3 OPTN is a candidate target of metformin for autophagy inhibition

**FIGURE 3:**
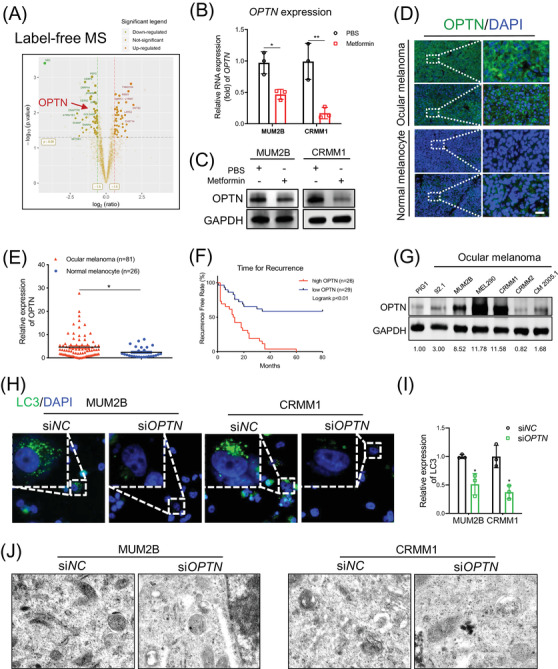
The expression and prognostic value of OPTN in ocular melanoma. (A). Volcano plots of label‐free MS showed the differentially expressed proteins in UM cells between the metformin‐treated group (1.0 mM for 24 h) and the control group. (B) Real‐time PCR showed OPTN expression in ocular melanoma cells (MUM2B, CRMM1) after treatment with 1.0 mM metformin for 24 h. (C) Western blot analysis showed OPTN expression in ocular melanoma cells (MUM2B, CRMM1) after treatment with 1.0 mM metformin for 24 h. GAPDH was used as a loading control. (D) Representative IF images showing OPTN expression in clinical samples of ocular melanoma and normal tissues. Scale bar: 50 μm. (E) Statistical results of OPTN expression in ocular melanoma and normal tissues. Data were presented as the mean ± SEM. *p < .05, ***p < .001. (F) Kaplan–Meier analysis revealed the correlation between OPTN expression and recurrence‐free rate in the internal cohort; *n* = 55, log‐rank test, *p* < .01. (G) Western blotting was carried out to detect the OPTN protein expression levels in ocular melanoma cells (92.1, MUM2B, MEL290, CRMM1, CRMM2, CM2005.1) and normal melanocytes (PIG1). GAPDH was used as an internal control. (H and I) Representative IF images showing the accumulation of LC3 puncta in MUM2B and CRMM1 cells after OPTN silencing (H). LC3 expression is presented as the mean ± SEM (I). *p < .05, **p < .01. Scale bar: 20 μm. (J) Representative TEM images showed autophagosomes in MUM2B and CRMM1 cells after OPTN knockdown, and red arrows indicate autophagosome. Scale bar: 500 nm

Through a high‐throughput proteomic assay of 3106 proteins with 19 352 identified peptides, we identified 47 upregulated proteins and 53 downregulated proteins (FC > 1.5 or <0.67, *p* < .05, unique peptide ≥2, three individual replicates; Figure S5). These differentially expressed proteins were mainly found in mitochondrial‐ and ribosomal‐associated proteins (Figure S6). Among these proteins, OPTN was the only protein that has been proven to be an activator of autophagy. We then chose OPTN as the candidate target of metformin that could be responsible for the inhibition of autophagy (Figure S7 and Figure 3A). We further confirmed that OPTN was indeed downregulated after metformin treatment at both RNA (Figure 3B) and protein (Figure 3C) levels.

Furthermore, through a cohort of 81 ocular melanoma patients (Tables S2 and S3), we found that OPTN was significantly upregulated in ocular melanoma samples compared to normal nevus tissue samples (Figure 3D,E). More importantly, follow‐up of 55 patients (26 patients were excluded from the cohort due to loss of contact) showed patients with high OPTN expression levels (*n* = 26) presented worse prognosis with a higher local recurrence rate (Figure 3F, log‐rank *p* < .01) than patients with low OPTN expression levels (*n* = 29). In addition, the TCGA cohort analysis showed that excessive expression of OPTN was associated with unfavourable prognosis in terms of disease‐free survival (Figure S8, log‐rank *p* < .01). In addition, we observed that OPTN was significantly upregulated in ocular melanoma cells at both RNA (Figure S9A) and protein levels (Figure 3G). To further reveal the role of OPTN in autophagy regulation, we first silenced OPTN expression with two small‐interfering RNAs (siRNAs) (Figure S9B). Notably, in contrast to the results in rapamycin‐treated melanoma cells, OPTN inhibition significantly attenuated global autophagic flux in MUM2B cells, with a decreased LC3 II/I ratio, Beclin1 level and ATG5 level and increased p62 accumulation, which is in accordance with the metformin‐treated group (Figure S9C). In addition, we observed a decreased number of LC3 puncta (Figure 3H,I) and autophagosomes (Figure 3J) in ocular melanoma cells. Taken together, our data show that OPTN is a key autophagic regulator in ocular melanoma cells and may serve as a downstream factor of metformin.

### 2.4 OPTN promoted tumourigenesis of ocular melanoma in vitro and in vivo

**FIGURE 4:**
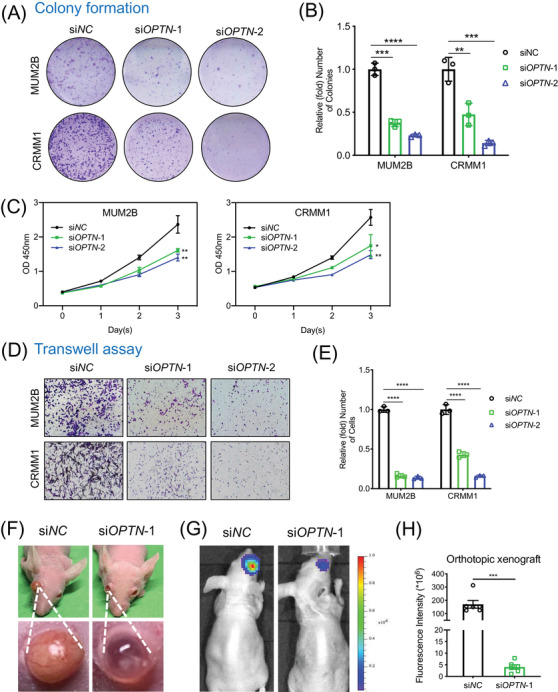
OPTN knockdown inhibits ocular melanoma growth in vitro and in vivo. (A and B) Plate colony formation assay was used to assess the growth rate of MUM2B and CRMM1 cells after OPTN knockdown. Statistical analyses of the colonies are presented as the mean ± SD. **p < .01, ***p < .001, ****p < .0001. (C) CCK‐8 assay was performed to measure the proliferation ability of MUM2B and CRMM1 cells after OPTN silencing. *p < .05, **p < .01. (D and E) Transwell assay was performed to evaluate the migration ability of MUM2B and CRMM1 cells treated with si*OPTN*s. The migrated cells were counted and are presented as the mean ± SD. ****p < .0001. (F) The overall and eyeball appearance of the mice showed the suppressive effects of OPTN knockdown on tumour volume in an orthotopic xenograft model. (G and H) The animal imaging system demonstrated the suppressive effects of OPTN silencing on tumour bioluminescent signals in orthotopic xenografts. Data are presented as the mean ± SEM. ***p < .001

As OPTN was significantly upregulated in tumours and modulated autophagic flux in ocular melanoma, we explored the role of OPTN in the tumour progression of ocular melanoma. Notably, the OPTN‐silenced group formed fewer and smaller colonies than the scramble RNA group (Figure 4A,B). Additionally, the CCK‐8 assay showed an attenuated proliferation rate after OPTN inhibition in MUM2B and CRMM1 cell lines (Figure 4C). Moreover, the migration ability was significantly attenuated in OPTN‐silenced tumour cells (Figure 4D,E). Furthermore, we verified the function of OPTN in ocular melanoma in vivo through an intraocular xenograft tumour model combined with luciferase animal imaging (Figure 4F). The results showed that OPTN‐inhibited MUM2B cells presented with a significantly decreased bioluminescence intensity (Figure 4G,H). Taken together, these results showed that OPTN was an oncogene in ocular melanoma.

### 2.5 Reintroduction of OPTN partially rescued metformin‐induced autophagic inhibition

**FIGURE 5:**
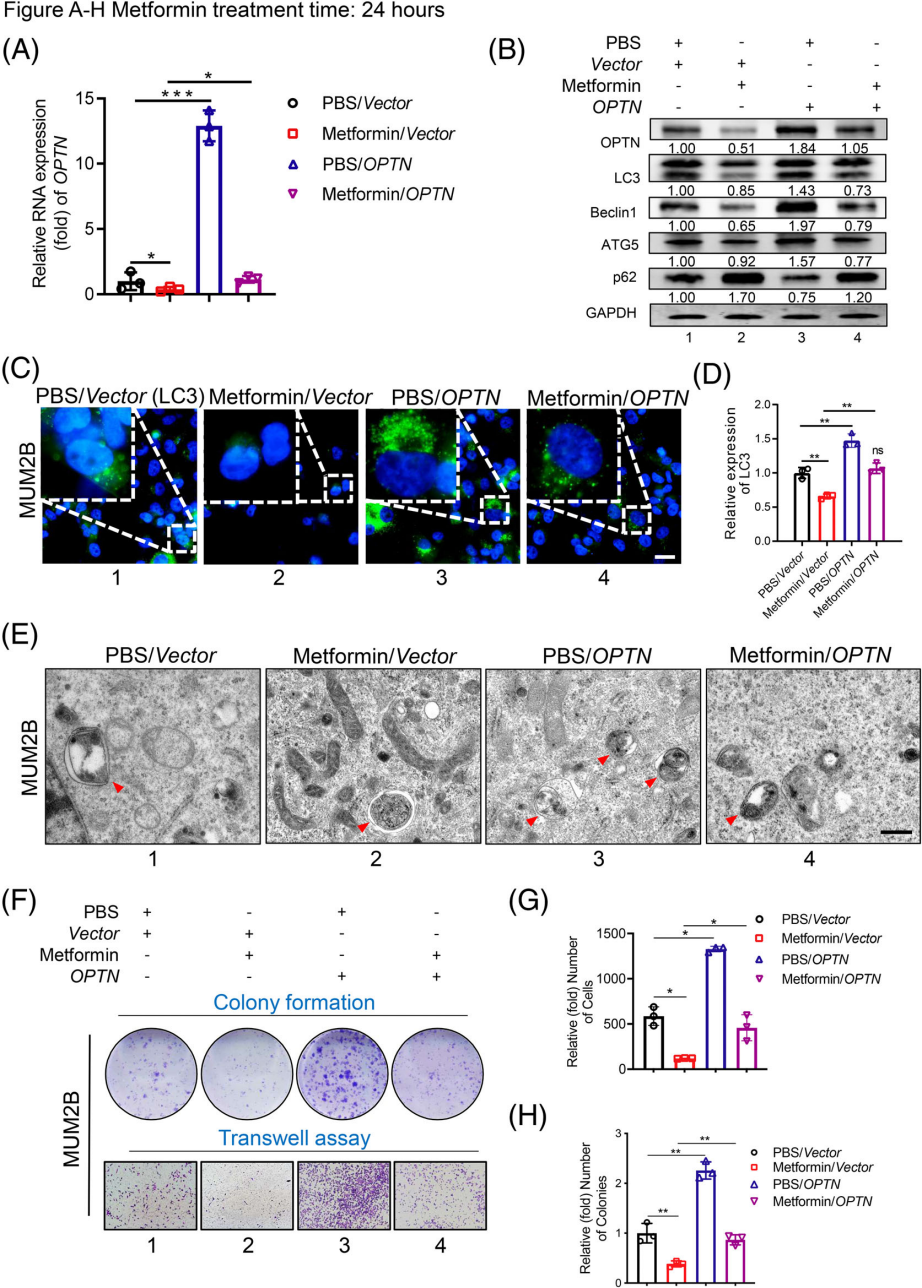
Overexpressing OPTN rescues the metformin‐mediated inhibition of autophagy, proliferation and migration of tumour cells. (A) Real‐time PCR showed OPTN expression levels in tumour cells treated with metformin (1.0 mM) alone, transfected with OPTN alone or treated/transfected with both. (B) Western blot analysis showed the protein levels of OPTN and autophagic flux‐related markers in tumour cells treated with metformin (1.0 mM) alone, transfected with OPTN alone or treated/transfected with both. GAPDH was used as a loading control. (C and D) Representative IF images demonstrating the accumulation of LC3 puncta in MUM2B cells treated with metformin (1.0 mM) alone, transfected with OPTN alone or treated/transfected with both. The relative LC3 expression is presented as the mean ± SEM. Scale bar: 20 μm. **p < .01. (E) Representative TEM images showing autophagosomes in MUM2B cells treated with metformin (1.0 mM) alone, transfected with OPTN alone or treated/transfected with both. Red arrows indicate autophagosome. Scale bar: 500 nm. (F–H) Plate colony formation assay and transwell migration assay were performed to demonstrate the proliferation ability and migration ability of MUM2B cells after treatment with metformin (1.0 mM) alone, transfection with OPTN alone or treatment/transfection with both. Statistical analyses of the colonies (G) and migrated cells (H) are shown. *p < .05, **p < .01

To further verify the relationship between metformin‐guided autophagy inhibition and OPTN expression, we rescued OPTN expression after metformin treatment in ocular melanoma cells by overexpressing OPTN (Figure 5A, PBS/OPTN group). Through RT‐qPCR analysis, we found that OPTN expression returned to the basal level after metformin treatment under simultaneous treatment with the OPTN overexpression vector (Figure 5A, metformin/OPTN group). More importantly, after reintroducing OPTN expression, we found that the decreased autophagy levels were restored, along with an elevated LC3 II/I ratio, Beclin1 and ATG5 levels and decreased p62 level compared to those in metformin‐treated cells (Figure 5B, lanes 2 and 4). In addition, the numbers of LC3 puncta (Figure 5C,D, lanes 1, 2 and 4) and autophagosomes (Figure 5E, lanes 1, 2 and 4) were restored to basal levels after overexpressing OPTN in the metformin‐treated group. Accordingly, the cellular migration and proliferation abilities were also restored after overexpressing OPTN in metformin‐treated cells (Figure 5F–H, lanes 1, 2 and 4 and Figure S10). In addition, the gain of OPTN also promoted autophagic flux (Figure 5B,C,E, lanes 1 and 3) and accelerated tumour migration and proliferation (Figure 5F–H, lanes 1 and 3 and Figure S10). Overall, these data indicated that OPTN was the downstream regulator of metformin‐guided autophagy inhibition.

### 2.6 Metformin epigenetically silenced OPTN through histone deacetylation in the OPTN promoter

**FIGURE 6:**
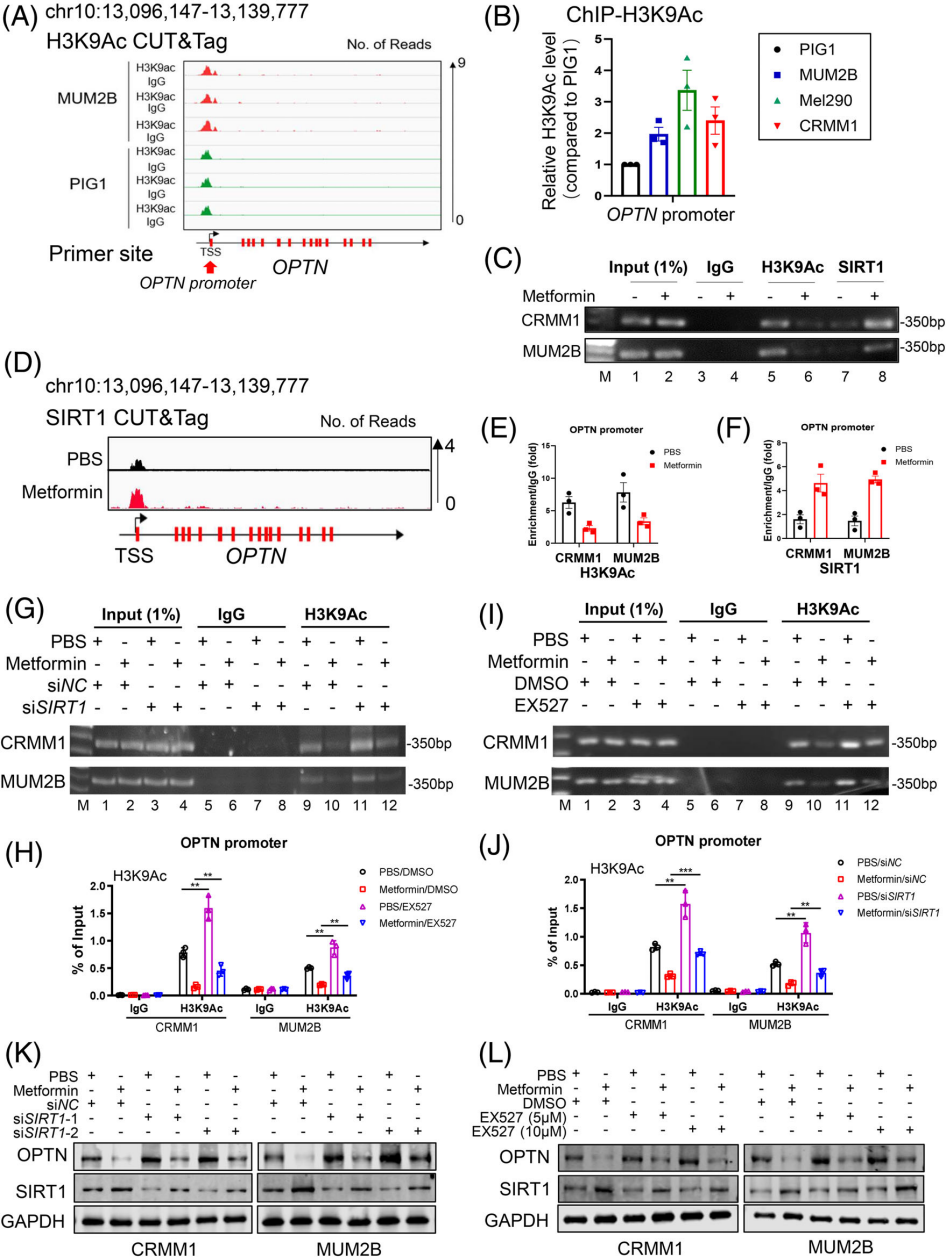
Metformin promotes SIRT1 binding and thus attenuates H3K9 acetylation in the OPTN promoter region. (A) CUT&Tag assay of H3K9Ac status in the OPTN promoter in MUM2B cells and normal PIG1 cells. (B) ChIP‐qPCR assay of H3K9Ac status in the OPTN promoter region in ocular melanoma cells (MUM2B, MEL290, CRMM1) and normal control cells (PIG1). (C) ChIP‐PCR assay of the H3K9Ac and SIRT1 status in the OPTN promoter region after treatment with 1.0‐mM metformin for 24 h in ocular melanoma cells (CRMM1, MUM2B). The representative outcome of agarose gel electrophoresis is shown. (D) CUT&Tag assay of SIRT1 status in the OPTN promoter region of MUM2B cells treated with metformin (1.0 mM, 24 h). (E and F) Statistical analyses of H3K9Ac enrichment (D) and SIRT1 enrichment (E) at the OPTN promotor are shown. *p < .05. (G–J) ChIP assay of H3K9Ac status in the OPTN promoter region after treatment with metformin, siSIRT1, and both in CRMM1 (G and H) and MUM2B cells (I and J). Statistical analyses of H3K9Ac enrichment in CRMM1 (H) and MUM2B cells (J) are presented. **p < .01. (K) Western blot analyses showed the protein levels of OPTN and SIRT1 in CRMM1 and MUM2B cells treated with metformin (1.0 mM) alone, siSIRT1s alone or both. GAPDH was used as a loading control. (L) Western blot analyses showed the protein levels of OPTN and SIRT1 in CRMM1 and MUM2B cells treated with metformin (1.0 mM) alone, EX527 (5 or 10 μM) alone or both. GAPDH was a loading control

**FIGURE 7:**
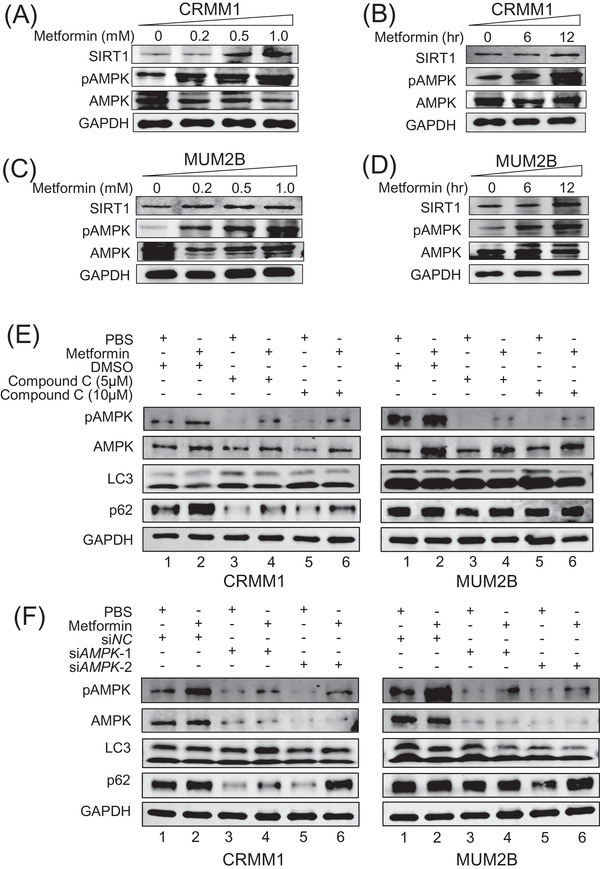
Metformin induces autophagy inhibition in an AMPK‐dependent way. (A) Western blot assay was carried out for p‐AMPK (Thr172), total AMPK and SIRT1 in CM cells (CRMM1) after treatment with gradient concentrations of metformin for 24 h. GAPDH was a loading control. (B) Western blot assay was performed to detect p‐AMPK (Thr172), total AMPK and SIRT1 in CM cells (CRMM1) after treatment with 1.0 mM metformin for different durations. GAPDH was a loading control. (C) Western blot assay was carried out for p‐AMPK (Thr172), total AMPK and SIRT1 in UM cells (MUM2B) after treatment with gradient concentrations of metformin. GAPDH was a loading control. (D) Western blot assay was performed to detect p‐AMPK (Thr172), total AMPK and SIRT1 in UM cells (MUM2B) after treatment with 1.0‐mM metformin for different durations. GAPDH was used as a loading control. (E) Western blot analyses showed the protein levels of p‐AMPK (Thr172), total AMPK, LC3 II/I and p62 in CRMM1 and MUM2B cells treated with metformin (1.0 mM) alone, Compound C (5 or 10 μM) alone or both. GAPDH was a loading control. (F) Western blot analyses showed the protein levels of p‐AMPK (Thr172), total AMPK, LC3 II/I and p62 in CRMM1 and MUM2B cells treated with metformin (1.0 mM) alone, si*AMPK*s alone or both. GAPDH was used as a loading control

OPTN was significantly downregulated after treatment with metformin, so we explored the mechanism underlying metformin‐induced OPTN downregulation. Because histone acetylation is an epigenetic modification involved in the maintenance of gene expression, we hypothesised that OPTN could be downregulated by the loss of histone acetylation. First, through H3K9Ac Cleavage Under Targets and Tagmentation (CUT&Tag) analysis, we found high histone acetylation level in the *OPTN* promoter in ocular melanoma cells (MUM2B) compared to normal pigmented cells (PIG1) (https://www.ncbi.nlm.nih.gov/geo/, accession number: GSE162573; Figure 6A). Similarly, we further observed a significant increase in the histone acetylation level of *OPTN* promoter in metformin‐treated ocular melanoma cells through the chromatin immunoprecipitation (ChIP) assay (Figure 6B). The results demonstrated that the histone acetylation level was significantly upregulated in ocular melanoma cells, which is in accordance with the specific OPTN upregulation in ocular melanomas. Moreover, we found that after metformin treatment, the histone acetylation level of the OPTN promoter was significantly downregulated in ocular melanoma cells (Figure 6C, lanes 5 and 6, Figure 6E). Because SIRT1 has been proven to be modulated by AMPK phosphorylation and is an important histone deacetylase, we were then interested in testing whether metformin attenuated histone acetylation by enhancing SIRT1 docking to the target zone. As expected, through CUT&Tag of SIRT1 in ocular melanomas (https://www.biosino.org/, accession number: OEP002155; Figure S11), we observed a significant promotion of SIRT1 binding to the OPTN promoter in metformin‐treated cells (Figure 6C, lanes 7 and 8, Figure 6D,F and Table S4). To further verify the relationship between OPTN and SIRT1 mediated by H3K9Ac in response to metformin, we performed loss‐of‐function assay by silencing SIRT1 through si*RNA* (Figure 6G,H) and EX527 (SIRT1 inhibitor, Figure 6I,J). First, we found H3K9Ac level was significantly increased after knocking down SIRT1 (Figure 6G, lane 11) and EX527 treatment (Figure 6I, lane 11). More importantly, the H3K9Ac level in *OPTN* promoter was decreased after metformin treatment (Figure 6G–J, lanes 9 and 10); however, H3K9Ac level largely restored after si*SIRT1* (Figure 6F,G, lane 12) and EX527 treatment (Figure 6I,J, lane 12) in ocular melanoma cells. This data indicated metformin‐induced H3K9Ac is dependent on SIRT1. As expected, OPTN protein was also decreased after metformin treatment, and SIRT1 inhibition restored OPTN expression, either by SIRT1 si*RNA* (Figure 6K) or EX527 (Figure 6L). Moreover, we determined that SIRT1 was significantly upregulated along with enhanced AMPK phosphorylation in a dose‐ (Figure 7A,C) and time‐dependent (Figure 7B,D) manner after stimulation with metformin in ocular melanoma cells. In order to further verify autophagic regulation of p‐AMPK by metformin, we added p‐AMPK inhibitors (Compound C) and AMPK si*RNA*s. Importantly, we found metformin‐induced p‐AMPK activation was impaired by Compound C (Figure 7E, lanes 1, 4 and 6) and si*AMPK*s (Figure 7F, lanes 1, 4 and 6). More importantly, metformin‐decreased LC3 II/I ratio was then rescued after Compound C treatment and AMPK silencing (Figure 7E,F, panel 4). Consistently, metformin treatment increased p62 level, while this phenotype was largely compromised by Compound C and si*AMPK*s (Figure 7E,F, panel 5).

Furthermore, to unveil the relationship between SIRT1 and AMPK in autophagy regulation, we overexpressed SIRT1 in AMPK‐deficient ocular melanoma cells. As expected, SIRT1 overexpression significantly inhibited autophagy, with elevated p62 and decreased ratio of LC3 II/I (Figure S12, lanes 1 and 2). Importantly, we found siAMPK‐induced autophagy activation was compromised after reintroducing SIRT1 (Figure S12, lanes 3 and 4).

Taken together, these data confirmed OPTN was regulated by SIRT1 mediated by H3K9Ac in response to metformin, metformin induced autophagic inhibition was dependent on AMPK activation, and SIRT1 functioned as a necessary downstream factor of AMPK induced autophagy regulation.

**FIGURE 8:**
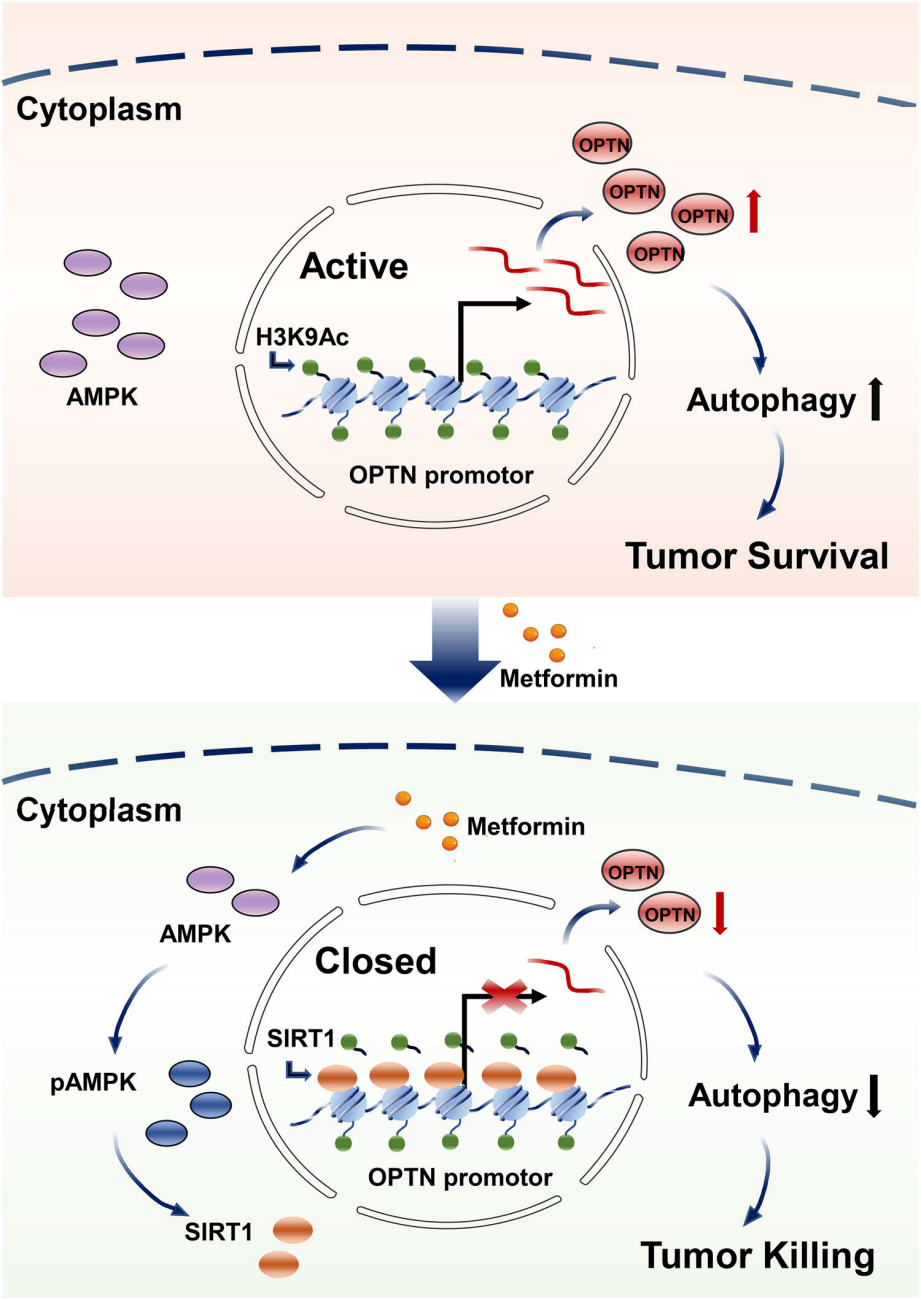
Proposed mechanism of action of metformin in ocular melanoma. Metformin upregulates the phosphorylation of AMPK and SIRT1; SIRT1 binding to OPTN promoter deacetylates H3K9, thus deceasing the transcription of OPTN; the decrease in OPTN inhibits the autophagic flux of ocular melanoma cells, leading to tumour killing

Conclusively, these data illustrated a novel pattern in which metformin enhances SIRT1 binding to the OPTN promoter, promotes histone deacetylation and silences OPTN expression (Figure 8).

## 3 DISCUSSION

In this study, we revealed for the first time that metformin is a promising agent for the treatment of ocular melanomas. The working concentration of 1 mM would be clinically feasible through intravitreal drug delivery, which allows reaching high drug concentrations in the vitreous cavity and avoiding adverse effects of systemic drug administration.^22^ For instance, previous studies have indicated intravitreal drug delivery has achieved a high concentration of amikacin, bevacizumab and 5‐fluorouracil.^23^, ^24^, ^25^ In addition, numerous mechanisms underlying metformin‐induced tumour inhibition have been discovered. For example, metformin downregulates YAP by disturbing IRF‐1 binding to the YAP promoter region in non‐small cell lung cancer.^26^, ^27^ Metformin could directly target the H3K27me3 demethylase KDM6A/UTX, a major epigenetic oncodriver.^28^ However, the role of metformin in ocular melanoma remains unreported. Here, we revealed for the first time that metformin inhibited the tumourigenesis of ocular melanoma by suppressing autophagic flux.

Autophagy is a highly conserved catabolic process involving the formation of autophagosomes that engulf cellular organelles and proteins passing to the lysosome.^29^ Because autophagy plays a dual function in cancer, the modulation of autophagy during tumourigenesis, either by reversing cytoprotective autophagy or by promoting cytotoxic autophagy, could potentially overcome cancer resistance to common chemotherapy.^12^, ^30^ For example, autophagy could protect tumour cells from killing. Thioredoxin domain‐containing 17 (TXNDC17) promotes paclitaxel resistance by inducing autophagy in ovarian cancer.^31^ Inhibition of deacetylase reduced pancreatic neuroendocrine neoplasm cells by restoring autophagic function without the involvement of AMPK.^32^ However, autophagy also facilitates tumour cell survival under stress conditions. For instance, autophagy induced by hypoxia mediates resistance of cisplatin in lung cancer cells.^33^, ^34^ Overall, how autophagy works in cancer treatment remains controversial. In addition, we found metformin played a dual‐edge sword role in the autophagy induction. Metformin induced autophagy in many cancers, such as osteosarcoma, gastric cancer and cutaneous melanoma.^35^, ^36^, ^37^ However, ocular melanoma presented with a distinct genetic pattern and presented with different tumour behaviour compared to cutaneous melanoma. Moreover, recent study has also shown that metformin could alleviate autophagy in ocular pigmented cells, which is the precursor of intraocular melanoma.^13^

AMPK activation serves a necessary inducer for autophagy under most circumstances.^38^ However, in some cases, AMPK activation does not always correlate with increased autophagy level. For example, in neurons, although metformin induced a significant promotion of AMPK phosphorylation, however, autophagic flux was attenuated.^39^ AMPK activation‐induced autophagy requires the mTOR inhibition.^40^ However, in ocular melanoma cells, metformin did not sufficiently inhibit mTOR phosphorylation signal, which is also consistent with the study in neurons. Here, we revealed for the first time that metformin induced autophagic inhibition in ocular melanoma cells, despite AMPK was remarkably activated.

Histone modifications regulate gene transcription and chromatin structure.^41^ Dysregulation of histone modification enzymes is correlated to tumourigenesis and development.^42^ To date, aberrant histone modifications have been revealed to be associated with autophagic regulation. For instance, histone deacetylation of NAT10 promotes the transition from rRNA biogenesis to autophagy upon energy stress.^43^ In addition, the histone deacetylase SIRT1 has been revealed to inhibit corticosterone‐induced autophagy.^44^ Nonetheless, the role of histone modifications in autophagic regulation, especially during tumourigenesis, is not well understood. Here, we revealed that SIRT1 functioned as a necessary downstream factor of AMPK‐induced autophagy regulation in ocular melanoma cells, and histone hyperacetylation of the OPTN promoter is associated with autophagy induction in ocular melanoma, which provides a novel pattern of histone remodelling‐guided autophagic regulation. Besides OPTN, we found that a large number of promoters (1383 genes) presented stronger SIRT1‐binding signal in metformin‐treated cells, which indicated metformin could function as an important epigenetic regulator in UM cells. It should be noted that OPTN is a multifunctional protein involved in the regulation of autophagy and secretion and is reported to be associated with several human diseases.^45^ For example, the phosphorylation of OPTN by TBK1 promotes binding to ubiquitin chains and enhances the selective autophagy of damaged mitochondria.^46^ In addition, the E478G mutation of OPTN promotes inflammation and induces neuronal cell death.^47^ Moreover, ubiquitylation of OPTN activates selective autophagy resulting in tumour suppression.^48^ However, how OPTN functions in the tumourigenesis of ocular melanoma is to be fully addressed. Here, we revealed for the first time that OPTN acts as an oncogene in ocular melanomas in which it is specifically highly expressed, and is associated with unfavourable outcomes in tumour patients.

In conclusion, we revealed that metformin significantly inhibited the tumour progression of ocular melanoma. In addition, we demonstrated that metformin acts as an autophagy inhibitor through histone deacetylation of OPTN, which serves as a novel oncogene in ocular melanoma. These studies provide novel insights into metformin‐guided tumour suppression of malignant ocular melanoma and the potential mechanism underlying the dual role of metformin in autophagy regulation.

## 4 MATERIALS AND METHODS

### 4.1 Cell lines and cell culture

The human CM cells (CRMM1, CRMM2 and CM2005.1) and the human UM cell MEL290 were gifts from Professor Martine J. Jager in Leiden University Medical Center. The UM cell MUM2B was kindly provided by Professor John F. Marshall from Tumour Biology Laboratory, Cancer Research UK Clinical Center. The UM cell 92.1 and normal cell ARPE‐19 were purchased from ATCC. PIG1, the human normal melanocyte, was a gift from the Department of Ophthalmology, Peking University Third Hospital. The culture medium used for UM cell lines and ARPE‐19 cells was Dulbecco's modified Eagle's medium (DMEM; Gibco). The CM cell lines and PIG1 cells were cultured in Ham's F12K medium (Gibco). All the culture media were supplemented with 10% fetal bovine serum (FBS; Gibco) and 1% penicillin/streptomycin. The cells were cultured at 37°C in a humidified 5% CO_2_ atmosphere. All the in vitro cell lines were validated by STR profiling and confirmed to be mycoplasma‐free.

### 4.2 CCK‐8 assay

UM, CM and PIG1 cells were seeded, 2000–3000 cells per well in 96‐well plates. Four hours before detection, 10 μl of Cell Counting Kit‐8 solution (CCK‐8; Dojindo, CK04) was added into each well. After incubation at 37°C for 2–4 h, the absorbance at 450 nm was measured with a microplate reader (Thermo Scientific) and recorded. Each independent experiment was performed three times.

### 4.3 Plate colony formation assay

UM, CM and PIG1 cells were seeded, 1000 cells per well in six‐well plates (Corning) and cultured with 2.0 ml of complete medium. After 1–2 weeks of incubation at 37°C, the plates were fixed with methanol and stained with 1% crystal violet for at least 30 min. Then, extra dye was washed, and the colonies were imaged and counted.

### 4.4 Cell migration assay

To measure the migration ability of tumour cells, we used a 24‐well transwell system (Corning) with 8‐μm pore size polycarbonate filters (Corning). Tumour cells (1.0 × 10^4^–1.0 × 10^5^) were suspended in appropriate culture medium with 2% FBS and added to the upper chambers, while the lower chambers contained culture medium with 10% FBS. After incubation for 24 h, cells in the chambers were fixed with methanol and stained with 1% crystal violet (Amresco, C8470). Cells in the upper chambers were removed, and those that migrated to the lower chambers were imaged.

### 4.5 Orthotopic xenograft assay

All animal experiments were approved by the Animal Ethics Committee of Shanghai Jiao Tong University School of Medicine, and were performed in accordance with institutional and international guidelines for animal care and use. Four‐week‐old BALB/c nude mice were obtained from the Slack Company and raised in the SPF Laboratory Animal room in the Hospital. After deep anaesthesia, 2 μl sterile PBS containing 2 × 10^4^ UM cells (MUM2B cells treated with PBS or metformin; MUM2B cells treated with si*NC* or si*OPTN*) was injected into the vitreous of the right eye with a Hamilton syringe under a surgical microscope. Then the eyes were treated with erythromycin eye ointment. Fourteen days later, bioluminescence was detected by in vivo small animal imaging systems. All mice were subjected to cervical dislocation 30 days after tumour cell implantation.

### 4.6 Western blot assay

Cells were rinsed, harvested, lysed and centrifuged. After revolving by SDS‐PAGE, protein samples were transferred to polyvinylidene fluoride (PVDF) membranes (Millipore, IEVH08100) and incubated in 5% milk TBS‐T for 1 h. The PVDF membranes were first incubated with a primary antibody overnight at 4°C, and then with a secondary antibody conjugating to a fluorescent tag (Invitrogen). The band signals were tested using the Odyssey Infrared Imaging System (LI‐COR, USA). Antibodies against the following antigens were used: OPTN (1:2000, 10837‐1‐AP, Proteintech), LC3 (1:1000, 14600‐1‐AP, Proteintech), Beclin1 (1:5000, 11306‐1‐AP, Proteintech), ATG5 (1:5000, ab108327, Abcam), p62 (1:10,000, ab109012, Abcam), SIRT1 (1:20,000, ab32441, Abcam), AMPK (1:1000, ab207442, Abcam), p‐AMPK (1:1000, #2535T, Cell Signaling Technology) and GAPDH (1:10,000, 60004‐1‐lg, Proteintech). The raw images have been displayed in Figures S13–S17.

### 4.7 Immunofluorescence (IF)

For GFP‐LC3 fluorescence microscopy, cells were cultured on coverslips in 12‐well plates (Corning), fixed with methanol, and blocked with 5%–10% goat serum (Gibco) for 1 h at room temperature. Next, the cells were incubated with LC3 primary antibody (1:200) at 4°C for 8 h, and then with fluorescently conjugated secondary antibody (Alexa Fluor 546, 1:800) for 1 h at room temperature. Cell nuclei were counterstained with diamidino‐2‐phenylindole (DAPI, Invitrogen). For tandem mRFP‐GFP‐LC3 fluorescence microscopy, cells were cultured in 12‐well plates, transfected with mRFP‐GFP‐LC3 lentivirus construct (Hanbio) and imaged alive. Images were taken with a fluorescence microscope (Olympus BX51).

### 4.8 Transmission electron microscopy (TEM)

First, MUM2B and CRMM1 cells were fixed with 2.5% glutaraldehyde solution (Sigma‐Aldrich, G5882) overnight. Then, cells were postfixed with 1% OsO4, and then dehydrated with graded ethanol. Finally, cells were embedded in 812 resin (Ted Pella, 18109). Thin sections were sliced and stained with 2% uranyl acetate. Images were viewed and recorded with a Tecnai 10 TEM (Philips).

### 4.9 Label‐free protein mass spectrometry (MS)

Label‐free MS was carried out as previously described.^21^ Briefly, MUM2B cell samples (three replicates, 50 μg each) were subjected to tryptic digestion. Quantitative label‐free proteomic MS was performed by nano‐UPLC (EASY‐nLC1200) coupled to a Q‐Exactive mass spectrometer (Thermo Finnigan). Raw MS files were processed with MaxQuant (version 1.5.6.0). The protein sequence data (Uniprot_organism_2016_09) were downloaded from UniProt. The differentially expressed proteins were further analysed according to GO database and KEGG pathway database.

### 4.10 RNA extraction and real‐time PCR

Total RNA was extracted with TRIzol reagent (Invitrogen). Reverse transcription was performed with a PrimeScript RT‐PCR kit (Takara Biotechnology). Real‐time PCR was performed with a standard SYBR Green PCR kit (Applied Biosystems, Thermo Fisher). The thermal conditions were set as follows: 60°C for 1 min, 95°C for 15 min, 45 cycles of 95°C for 15 s, and the optimised annealing temperature for 1 min. GAPDH was used as an internal control. All specific primers are listed in Table S1.

### 4.11 Tissue specimens

From 2007 to 2017, 88 human ocular melanoma samples and 28 human normal melanocytic nevi samples in total were collected and pathologically confirmed at Shanghai Ninth People's Hospital. Their clinicopathological characteristics are listed in Tables S2 and S3.

### 4.12 SiRNA

The OPTN silencing was performed using siRNA. MUM2B and CRMM1 cells were seeded in six‐well plates, about 300,000 cells per well and transfected with siRNA using Lipofectamine 2000 in Opti‐MEM (Gibco). Six hours later, the medium was replaced with fresh complete medium. RNA extraction, CCK‐8 assay, cell migration assay and plate colony formation assay were performed after 48 h, and protein extraction was performed after 72 h. All specific primers are listed in Table S1.

### 4.13 Plasmid construction

OPTN overexpression cassette was generated with PCR and cloned into the PGMLV‐CMV vector. The related primer sequences are listed in Table S1.

### 4.14 CUT&Tag, library construction and DNA sequencing

CUT&Tag assay was performed as per literature.^49^ Briefly, 100,000 cells were harvested, washed and mixed with activated concanavalin A‐coated magnetic beads (Bangs Laboratories) at room temperature for 15 min. The mix was resuspended in 100 μl DIG‐wash buffer consisting of 2‐mM EDTA and primary antibody (1:50 dilution) and incubated overnight at 4℃. The secondary antibody was added to the cells at a 1:50 dilution and incubated at room temperature for about 1 h. After washing, 100 μl of pA‐Tn5 adapter complex (∼40 nM) was added into the cells and incubated at room temperature for about 1 h. After washing, the cells were resuspended in Tagmentation buffer (50 μl) and incubated at 37°C for 1 h. Then, proteinase K treatment was used at 55°C for 30 min to stop the tagmentation and then at 70°C for 20 min to inactivate proteinase K. DNA was then extracted with AMPure XP beads (Beckham Counter) and eluted. For PCR, the following thermocycler programme was used: 72°C for 5 min, 98°C for 30 s, 14 cycles of 98°C for 10 s and 63°C for 30 s, final extension at 72°C for 1 min, and hold at 8°C. Pooled libraries were purified with 1.1× AMPure XP beads. Paired‐end Illumina sequencing was carried out on a HiSeq 4000 instrument (Illumina).

### 4.15 Chromatin immunoprecipitation

ChIP assays were performed as previously described with an EZ‐Magna ChIP A/G kit (Millipore).^50^ The experiments were conducted in accordance to the manufacturer's instructions. One hundred million cells were lysed with cell lysis buffer and nuclear lysis buffer. The cell lysates were sonicated for 10 min (10 s on and 15 s off) on ice. Antibodies against H3K9Ac (39038, Active Motif), SIRT1 (07‐131, Millipore) and IgG (ab172730, Abcam) were added into the 10‐fold diluted chromatin fragments. Then, 20 μl Protein A/G magnetic beads were used for each IP and incubated at 4°C with rotation overnight. After washing, crosslinking reversal and proteinase K treatment, the DNA was freed from the protein/DNA complexes. Purified DNA fragments were amplified with PCR at the following conditions: 95°C for 5 min, 34 cycles at 95°C for 30 s, the optimal annealing temperature for 30 s, and 72°C for 30 s of extension. The specific primers used for ChIP‐PCR analysis are listed in Table S1.

### 4.16 The Cancer Genome Atlas (TCGA) database

TCGA (http://www.cbioportal.org) database and GEPIA (http://gepia.cancer‐pku.cn/) were queried to validate the potential role of OPTN in UM. The database provides the transcriptional landscape and follow‐up information of 40 UM patients.

### 4.17 Statistical analysis

GraphPad Prism software (version 8.0) was applied for the statistical analyses. Data are presented as mean ± SD or mean ± SEM as required, and unpaired two‐tailed Student's *t*‐test was used to assess the differences between two groups. Survival plots were depicted with Kaplan–Meier curves, and *p*‐values were calculated with the log‐rank test. *p*‐Value <.05 was considered statistically significant, and denoted with asterisks (*p < .05, **p < .01, ***p < .001, ****p < .0001).

## CONFLICT OF INTEREST

The authors declare that there is no conflict of interest.

## Supporting information

Supporting Information

Supporting Information
