# Supplementary material for: Metformin promotes histone deacetylation of optineurin and suppresses tumour growth through autophagy inhibition in ocular melanoma
Source: Clin Transl Med. 2022 Jan 24;12(1):e660. doi: 10.1002/ctm2.660 (PMC8787022; doi:10.1002/ctm2.660)
Supplement: Supplementary file 1 — Supporting Information [file CTM2-12-e660-s001.docx]

**Metformin promotes histone deacetylation of optineurin and suppresses tumor growth through autophagy inhibition in ocular melanoma**

Ai Zhuang^1,2^; Peiwei Chai^1,2^; Shaoyun Wang^1,2^; Sipeng Zuo^1,2^; Jie Yu^1,2^; Shichong Jia^1,2^; Shengfang Ge^1,2^; Renbing Jia^1,2^; Yixiong Zhou^1,2^; Wodong Shi^1,2^; Xiaofang Xu^1,2^; Jing Ruan^1,2^; Xianqun Fan^1,2^

^1^Department of Ophthalmology, Ninth People’s Hospital, Shanghai Jiao Tong University School of Medicine, Shanghai, China.

^2^Shanghai Key Laboratory of Orbital Diseases and Ocular Oncology, Shanghai, China.

Ai Zhuang, Peiwei Chai and Shaoyun Wang contributed equally to this paper.

Xiaofang Xu, Jing Ruan and Xianqun Fan are the co-corresponding authors.

**Institutional addresses:**

^1^Department of Ophthalmology, Ninth People’s Hospital, Shanghai Jiao Tong University School of Medicine, No 639 Zhizaoju Road, Shanghai, China.

^2^Shanghai Key Laboratory of Orbital Diseases and Ocular Oncology, Shanghai, China.

No 639 Zhizaoju Road, Shanghai, 200011, China.

**E-mails:**

**Xiaofang Xu*:** xuxu0139@hotmail.com

**Jing Ruan*:** drjruan@163.com

**Xianqun Fan***: fanxq@sjtu.edu.cn;

**Funding:**

This work was supported by the National Natural Science Foundation of China (81600766), and the Science and Technology Commission of Shanghai (20DZ2270800).

**Supplementary files include 17 figures and 4 Tables**

**Table legends**

**Table S1.** Primers and siRNAs sequences used in this study.

**Table S2.** The clinical characteristics of ocular melanoma patient cohorts in tissue chip assay.

**Table S3.** The clinical characteristics of melanocytic nevus patient cohorts in tissue chip assay.

**Table S4.** Upregulated SIRT1 binding sites (in promoter, mRNA) after metformin treatment in ocular melanoma MUM2B cell.

**Figure Legends**

**Fig S1.** Flow cytometry was adopted to detect the effect of metformin (1.0mM, 24 h) on apoptosis in ocular melanoma cells (A, B).

**Fig S2.** Western blot analysis showed the protein levels of autophagic flux-related markers (LC3II/I, Beclin1, ATG5, p62) in ocular melanoma cells (MEL290) treated with a gradient concentration of metformin for 24 h (A) and with 1.0 mM metformin for different durations (B). GAPDH was used as a loading control. qPCR was used to test the transcription level of p62 in ocular melanoma cells treated with 1.0mM metformin for 24 h (C).

**Fig S3.** Metformin attenuate autophagic flux in ocular melanomas. (A) Diagram drawing of mRFP-GFP fluorescence microscopy. The yellow puncta represent early autophagosomes while autolysosomes are marked in red. (B) Representative images of mRFP-GFP fluorescence microscopy in CRMM1 and MUM2B cells. Scale Bar: 10μM. (C) Statistical analysis of mRFP-GFP fluorescence microscopy in 50 cells. The number of puncta in PBS/DMSO group has been set to 1. Statistical analysis was performed using two-tailed student’s t test (*/**/*** for autophagosomes and # for autolysosomes). *, # p<0.05; ** p<0.01; ***p<0.001

**Fig S4.** Western blot was applied to detect the protein levels of LC3 II/I and p62 in cervical cancer cell (Hela), prostatic cancer cell (PC-3), and breast cancer cell (MDA-MB-231) after treating with 1.0 mM metformin for 24 h. GAPDH was a loading control.

**Fig S5.** Total Ion Chromatography showed mass spectrum peaks of MUM2B cell samples treated with PBS or metformin (1.0 mM for 24 h) during the identifying process.

**Fig S6.** The differentially expressed proteins between metformin-treated (1.0 mM for 24 h) and PBS-treated MUM2B cells were selected for GO enrichment analysis, and the top ten GO entries were shown.

**Fig S7.** Clustering heatmap showed the significant proteins in comparison of the tumor cells treated with either PBS or metformin (1.0 mM for 24 h).

**Fig S8.** The TCGA database demonstrated prolonged disease-free survival time in patients with low OPTN expression.

**Fig S9.** (A) Real time-PCR was performed to test OPTN expression levels in ocular melanoma cells (MUM2B, MEL290, 92.1, CRMM1, CM2005.1) and normal melanocyte (PIG1). (B) Real time-PCR was used to confirm the OPTN expression levels in MUM2B and CRMM1 cells after OPTN silencing. (C) Western blot was used to analyze the protein levels of OPTN, LC3 II/I, Beclin1, ATG5, p62 in MUM2B cell treating with siOPTNs, metformin (1.0 mM, 24 h), and rapamycin (5 μM, 12 h). GAPDH was used as an internal control. *p<0.05, **p<0.01, ****p<0.0001.

**Fig S10.** CCK-8 assay was performed to test the proliferation abilities of MUM2B cells after treating with metformin (1.0 mM, 24 h) alone, overexpressing OPTN alone, and the combination. The absorbance of OD 450nm was recorded and presented as mean±SD. **p<0.01, ***p<0.001.

**Fig S11.** CUT&Tag of SIRT1 in MUM2B. The genome distribution of SIRT1 was shown. SIRT1 was enriched near TSS region in MUM2B cell. The data (bw file) was deposited in *https://www.biosino.org/*, under the accession number of OEP002155**.**

**Figure S12.** Western blot was used to analyze the protein levels of SIRT1, LC3 II/I, p62 in MUM2B and CRMM1 cells treating with pcDNA3.1-*SIRT1*, si*AMPK*, or both. GAPDH was used as an internal control.

**Fig S13-S17.** Unprocessed figures of western blot assays.

**Table S1. Primers and siRNAs sequences used in this study.**

| Primer name | Sequences (5'-3') | Purpose |
| --- | --- | --- |
| GAPDH-F | TGTTGCCATCAATGACCCCTT | PCR |
| GAPDH-R | CTCCACGACGTACTCAGCG |  |
| OPTN-F | CCGTACGCCTCTGTAAACCC | PCR |
| OPTN-R | GCAGAAGTTCCTGTGGAAAAGT |  |
| OPTN-siRNA1 |  | siRNA |
| Sence | GGAAGUUUACUGUUCUGAU |  |
| Antisense | AUCAGAACAGUAAACUUCC |  |
| OPTN-siRNA2 |  | siRNA |
| Sence | GCGGAAUAUUCCGAUUCAU |  |
| Antisense | AUGAAUCGGAAUAUUCCGC |  |
| OE-OPTN |  |  |
| Sence | CGAATTCGAAGTATACCTCGAGGCCACCATGTCCCATCAACCTCTCAGCTG |  |
| Antisense | CATGGTCTTTGTAGTCGGATCCAATGATGCAATCCATCACGTGAA |  |
| OPTN-F | GCCTTAGAGCAGTCCCTGTT | ChIP-PCR |
| OPTN-R | CCTGGAGAGAACTCCCGAC |  |

**Table S2. The clinical characteristics of ocular melanoma patient cohorts in tissue chip assay.**

| Patient No. | Gender (0=Female, 1=Male) | Age (years old) | Time for recurrence (month, 0=No recurrence) | Relative expression of OPTN |
| --- | --- | --- | --- | --- |
| 1 | 1 | 59 | 2 | 3.8 |
| 2 | 1 | 59 | 2 | 3.7 |
| 3 | 0 | 64 |  | 20.0 |
| 4 | 1 | 45 |  | 8.3 |
| 5 | 1 | 45 |  | 3.0 |
| 6 | 1 | 57 |  | 5.6 |
| 7 | 1 | 54 | 36 | 9.7 |
| 8 | 1 | 54 | 36 | 3.1 |
| 9 | 1 | 76 | 0 | 1.1 |
| 10 | 0 | 54 | 0 | 2.1 |
| 11 | 1 | 46 | 6 | 1.8 |
| 12 | 1 | 57 | 24 | 16.0 |
| 13 | 1 | 57 | 24 | 3.3 |
| 14 | 1 | 67 |  | 18.1 |
| 15 | 1 | 59 | 2 | 9.6 |
| 16 | 1 | 59 | 2 | 5.7 |
| 17 | 0 | 89 | 0 | 2.0 |
| 18 | 0 | 89 | 0 | 0.1 |
| 19 | 1 | 52 |  | 5.3 |
| 20 | 1 | 62 |  | 0.9 |
| 21 | 1 | 37 |  | 10.6 |
| 22 | 1 | 82 | 0 | 0.3 |
| 23 | 1 | 82 | 0 | 1.6 |
| 24 | 1 | 48 | 60 | 4.8 |
| 25 | 0 | 29 | 0 | 0.1 |
| 26 | 0 | 49 | 11 | 5.2 |
| 27 | 0 | 49 | 11 | 3.3 |
| 28 | 1 | 35 | 34 | 1.5 |
| 29 | 1 | 35 | 34 | 4.6 |
| 30 | 1 | 47 | 0 | 1.3 |
| 31 | 0 | 60 |  | 3.2 |
| 32 | 1 | 70 |  | 1.5 |
| 33 | 1 | 64 |  | 0.3 |
| 34 | 1 | 52 | 2 | 7.9 |
| 35 | 0 | 77 | 19 | 0.8 |
| 36 | 1 | 65 |  | 3.6 |
| 37 | 0 | 45 |  | 27.8 |
| 38 | 0 | 42 | 18 | 13.2 |
| 39 | 1 | 55 | 12 | 10.6 |
| 40 | 1 | 65 |  | 5.7 |
| 41 | 1 | 61 | 3 | 7.8 |
| 42 | 0 | 58 |  | 2.9 |
| 43 | 1 | 31 | 13 | 3.7 |
| 44 | 1 | 32 |  | 6.9 |
| 45 | 1 | 21 |  | 3.6 |
| 46 | 0 | 66 |  | 14.3 |
| 47 | 0 | 69 |  | 14.6 |
| 48 | 1 | 64 |  | 0.1 |
| 49 | 0 | 67 | 0 | 1.6 |
| 50 | 1 | 23 |  | 3.2 |
| 51 | 1 | 23 |  | 5.7 |
| 52 | 1 | 12 |  | 1.8 |
| 53 | 1 | 73 | 13 | 0.1 |
| 54 | 0 | 52 |  | 6.1 |
| 55 | 1 | 31 | 13 | 0.9 |
| 56 | 1 | 78 | 0 | 1.0 |
| 57 | 0 | 42 | 18 | 2.4 |
| 58 | 1 | 66 | 20 | 1.7 |
| 59 | 1 | 58 | 0 | 0.3 |
| 60 | 0 | 82 |  | 4.0 |
| 61 | 1 | 80 |  | 1.9 |
| 62 | 1 | 60 | 9 | 3.7 |
| 63 | 0 | 51 | 12 | 1.2 |
| 64 | 0 | 58 |  | 3.6 |
| 65 | 1 | 52 | 2 | 2.2 |
| 66 | 1 | 73 | 13 | 5.3 |
| 67 | 1 | 81 | 0 | 0.7 |
| 68 | 1 | 20 |  | 2.1 |
| 69 | 1 | 47 | 16 | 1.0 |
| 70 | 0 | 77 | 19 | 3.8 |
| 71 | 1 | 54 | 2 | 10.6 |
| 72 | 1 | 63 | 32 | 3.9 |
| 73 | 1 | 38 | 8 | 1.4 |
| 74 | 1 | 70 | 23 | 5.4 |
| 75 | 1 | 18 | 6 | 4.3 |
| 76 | 1 | 76 | 0 | 0.8 |
| 77 | 1 | 75 | 0 | 0.4 |
| 78 | 0 | 53 | 5 | 0.1 |
| 79 | 1 | 74 | 0 | 1.5 |
| 80 | 1 | 49 | 0 | 1.1 |
| 81 | 1 | 67 | 0 | 0.8 |

**Table S3. The clinical characteristics of melanocytic nevus patient cohorts in tissue chip assay.**

| Patient No. | Gender (0=Female, 1=Male) | Age (years old) | Relative expression of OPTN |
| --- | --- | --- | --- |
| 1 | 0 | 48 | 4.5 |
| 2 | 0 | 29 | 0.8 |
| 3 | 1 | 31 | 0.4 |
| 4 | 0 | 25 | 0.4 |
| 5 | 1 | 32 | 0.3 |
| 6 | 0 | 65 | 0.6 |
| 7 | 0 | 61 | 3.0 |
| 8 | 1 | 55 | 1.0 |
| 9 | 0 | 30 | 0.8 |
| 10 | 1 | 50 | 0.5 |
| 11 | 1 | 67 | 4.8 |
| 12 | 1 | 40 | 1.8 |
| 13 | 0 | 31 | 7.2 |
| 14 | 0 | 54 | 5.5 |
| 15 | 0 | 60 | 0.6 |
| 16 | 1 | 62 | 1.5 |
| 17 | 0 | 45 | 6.1 |
| 18 | 0 | 62 | 1.8 |
| 19 | 0 | 63 | 0.7 |
| 20 | 0 | 1 | 8.0 |
| 21 | 0 | 8 | 3.3 |
| 22 | 0 | 29 | 1.6 |
| 23 | 0 | 29 | 1.3 |
| 24 | 1 | 3 | 4.5 |
| 25 | 0 | 26 | 0.4 |
| 26 | 1 | 14 | 1.0 |

**Table S4. Upregulated SIRT1 binding sites (in promoter, mRNA) after metformin treatment in ocular melanoma MUM2B cell.**

| Chrom | Start | End | Annotation | geneID | distanceToTSS |
| --- | --- | --- | --- | --- | --- |
| chr1 | 20141542 | 20141715 | Promoter (2-3kb) | PLA2G2F | 2216 |
| chr1 | 20485252 | 20485538 | Promoter (<=1kb) | CAMK2N1 | 682 |
| chr1 | 23424271 | 23424559 | Promoter (<=1kb) | TCEA3 | 148 |
| chr1 | 24930472 | 24930741 | Promoter (<=1kb) | RUNX3 | -595 |
| chr1 | 24932080 | 24932272 | Promoter (2-3kb) | RUNX3 | -2203 |
| chr1 | 26045525 | 26045696 | Promoter (<=1kb) | SLC30A2 | 417 |
| chr1 | 26178372 | 26178549 | Promoter (<=1kb) | CNKSR1 | 863 |
| chr1 | 26188564 | 26188991 | Promoter (1-2kb) | CATSPER4 | -1570 |
| chr1 | 26282562 | 26282769 | Promoter (2-3kb) | SH3BGRL3 | 2440 |
| chr1 | 26410950 | 26411594 | Promoter (<=1kb) | LIN28A | 133 |
| chr1 | 26959769 | 26960074 | Promoter (<=1kb) | KDF1 | 311 |
| chr1 | 27341728 | 27342234 | Promoter (<=1kb) | SYTL1 | 0 |
| chr1 | 27382387 | 27382682 | Promoter (<=1kb) | CD164L2 | 621 |
| chr1 | 1598564 | 1598735 | Promoter (1-2kb) | FNDC10 | 1361 |
| chr1 | 30905970 | 30906315 | Promoter (2-3kb) | SDC3 | 2446 |
| chr1 | 30907380 | 30907774 | Promoter (<=1kb) | SDC3 | 987 |
| chr1 | 30908981 | 30909208 | Promoter (<=1kb) | SDC3 | -220 |
| chr1 | 31619242 | 31619487 | Promoter (<=1kb) | HCRTR1 | 49 |
| chr1 | 31798756 | 31799024 | Promoter (<=1kb) | SPOCD1 | 0 |
| chr1 | 32275238 | 32275561 | Promoter (1-2kb) | LCK | 1106 |
| chr1 | 32276343 | 32276779 | Promoter (2-3kb) | LCK | 2211 |
| chr1 | 32361413 | 32362040 | Promoter (<=1kb) | TSSK3 | -157 |
| chr1 | 32742486 | 32742748 | Promoter (<=1kb) | KIAA1522 | 601 |
| chr1 | 32868865 | 32869393 | Promoter (1-2kb) | FNDC5 | 1353 |
| chr1 | 1780916 | 1781129 | Promoter (<=1kb) | NADK | -847 |
| chr1 | 1781678 | 1781900 | Promoter (1-2kb) | NADK | -1609 |
| chr1 | 36099222 | 36099447 | Promoter (<=1kb) | COL8A2 | 802 |
| chr1 | 36126040 | 36126385 | Promoter (<=1kb) | COL8A2 | -820 |
| chr1 | 37753831 | 37754244 | Promoter (<=1kb) | EPHA10 | -862 |
| chr1 | 37930946 | 37931207 | Promoter (<=1kb) | INPP5B | 542 |
| chr1 | 39081707 | 39082089 | Promoter (<=1kb) | MACF1 | 290 |
| chr1 | 39671167 | 39671359 | Promoter (<=1kb) | NT5C1A | 679 |
| chr1 | 40783999 | 40784232 | Promoter (<=1kb) | KCNQ4 | 0 |
| chr1 | 40861960 | 40862603 | Promoter (<=1kb) | CITED4 | 0 |
| chr1 | 42166509 | 42166776 | Promoter (1-2kb) | GUCA2A | -1791 |
| chr1 | 42784993 | 42785264 | Promoter (<=1kb) | TMEM269 | 0 |
| chr1 | 43285348 | 43285593 | Promoter (<=1kb) | C1orf210 | 0 |
| chr1 | 43338449 | 43338845 | Promoter (<=1kb) | MPL | 600 |
| chr1 | 44406993 | 44407387 | Promoter (1-2kb) | RNF220 | 1705 |
| chr1 | 44843030 | 44843447 | Promoter (<=1kb) | PTCH2 | 0 |
| chr1 | 45622801 | 45623004 | Promoter (<=1kb) | CCDC17 | 963 |
| chr1 | 46173971 | 46174139 | Promoter (<=1kb) | TSPAN1 | -948 |
| chr1 | 47192824 | 47193310 | Promoter (1-2kb) | PDZK1IP1 | -1780 |
| chr1 | 47416813 | 47417276 | Promoter (<=1kb) | FOXE3 | 741 |
| chr1 | 48776716 | 48776921 | Promoter (<=1kb) | BEND5 | 0 |
| chr1 | 51345147 | 51345406 | Promoter (<=1kb) | TTC39A | -31 |
| chr1 | 54624359 | 54624616 | Promoter (<=1kb) | FAM151A | -803 |
| chr1 | 57422364 | 57422733 | Promoter (1-2kb) | DAB1 | 1324 |
| chr1 | 62194794 | 62195111 | Promoter (<=1kb) | L1TD1 | 0 |
| chr1 | 63771977 | 63772420 | Promoter (1-2kb) | ROR1 | -1602 |
| chr1 | 64471206 | 64471465 | Promoter (<=1kb) | CACHD1 | 414 |
| chr1 | 65148483 | 65149146 | Promoter (<=1kb) | AK4 | 280 |
| chr1 | 77281990 | 77282163 | Promoter (<=1kb) | AK5 | 0 |
| chr1 | 77531695 | 77532070 | Promoter (<=1kb) | AK5 | -37 |
| chr1 | 92480792 | 92481001 | Promoter (2-3kb) | GFI1 | 2953 |
| chr1 | 2473848 | 2474133 | Promoter (2-3kb) | PLCH2 | -2182 |
| chr1 | 109467418 | 109467588 | Promoter (<=1kb) | SYPL2 | 790 |
| chr1 | 110606141 | 110606336 | Promoter (<=1kb) | KCNA2 | -122 |
| chr1 | 111202813 | 111203011 | Promoter (1-2kb) | DENND2D | 1398 |
| chr1 | 111203971 | 111204216 | Promoter (<=1kb) | DENND2D | 193 |
| chr1 | 111347183 | 111347377 | Promoter (<=1kb) | PIFO | 563 |
| chr1 | 112465126 | 112465531 | Promoter (1-2kb) | WNT2B | -1010 |
| chr1 | 2800374 | 2800683 | Promoter (1-2kb) | TTC34 | 1034 |
| chr1 | 3066356 | 3066558 | Promoter (2-3kb) | PRDM16 | -2610 |
| chr1 | 149917702 | 149917967 | Promoter (<=1kb) | SV2A | 0 |
| chr1 | 150981353 | 150981572 | Promoter (<=1kb) | ANXA9 | -445 |
| chr1 | 151852525 | 151852910 | Promoter (<=1kb) | THEM5 | 787 |
| chr1 | 153459234 | 153459477 | Promoter (<=1kb) | S100A7 | 397 |
| chr1 | 153549105 | 153549293 | Promoter (<=1kb) | S100A3 | 0 |
| chr1 | 153681579 | 153681906 | Promoter (2-3kb) | NPR1 | 2942 |
| chr1 | 154501943 | 154502458 | Promoter (<=1kb) | SHE | 0 |
| chr1 | 154567986 | 154568164 | Promoter (<=1kb) | CHRNB2 | 151 |
| chr1 | 155185127 | 155185537 | Promoter (1-2kb) | AL713999.1 | 1735 |
| chr1 | 155300887 | 155301079 | Promoter (<=1kb) | PKLR | 0 |
| chr1 | 156211256 | 156211446 | Promoter (1-2kb) | PMF1-BGLAP | -1536 |
| chr1 | 156367691 | 156367896 | Promoter (1-2kb) | RHBG | -1316 |
| chr1 | 156893475 | 156893818 | Promoter (<=1kb) | PEAR1 | 0 |
| chr1 | 159862174 | 159862349 | Promoter (<=1kb) | VSIG8 | 308 |
| chr1 | 3533199 | 3533561 | Promoter (1-2kb) | MEGF6 | -1751 |
| chr1 | 161236098 | 161236271 | Promoter (<=1kb) | NR1I3 | 294 |
| chr1 | 161258879 | 161259228 | Promoter (<=1kb) | PCP4L1 | 152 |
| chr1 | 165699512 | 165699831 | Promoter (<=1kb) | ALDH9A1 | -649 |
| chr1 | 3652764 | 3653194 | Promoter (<=1kb) | TP73 | 199 |
| chr1 | 167087867 | 167088052 | Promoter (2-3kb) | GPA33 | 2579 |
| chr1 | 177171160 | 177171341 | Promoter (<=1kb) | BRINP2 | -156 |
| chr1 | 181035979 | 181036160 | Promoter (1-2kb) | MR1 | 1976 |
| chr1 | 181482712 | 181482917 | Promoter (<=1kb) | CACNA1E | -663 |
| chr1 | 196608809 | 196609018 | Promoter (<=1kb) | KCNT2 | 207 |
| chr1 | 197911079 | 197911559 | Promoter (<=1kb) | LHX9 | -932 |
| chr1 | 200872479 | 200872748 | Promoter (<=1kb) | GPR25 | -207 |
| chr1 | 200872942 | 200873764 | Promoter (<=1kb) | GPR25 | 0 |
| chr1 | 201283242 | 201283571 | Promoter (<=1kb) | PKP1 | 0 |
| chr1 | 202163563 | 202163795 | Promoter (1-2kb) | PTPN7 | -1982 |
| chr1 | 202195423 | 202195649 | Promoter (1-2kb) | LGR6 | 1522 |
| chr1 | 202644324 | 202644733 | Promoter (<=1kb) | SYT2 | -871 |
| chr1 | 202646104 | 202646462 | Promoter (2-3kb) | SYT2 | -2651 |
| chr1 | 202708561 | 202708804 | Promoter (1-2kb) | SYT2 | 1613 |
| chr1 | 203129050 | 203129448 | Promoter (<=1kb) | ADORA1 | 677 |
| chr1 | 6244630 | 6244844 | Promoter (<=1kb) | HES3 | 438 |
| chr1 | 6261964 | 6262287 | Promoter (<=1kb) | GPR153 | -989 |
| chr1 | 204359596 | 204359836 | Promoter (<=1kb) | PLEKHA6 | 80 |
| chr1 | 205259882 | 205260290 | Promoter (2-3kb) | TMCC2 | 2693 |
| chr1 | 205935726 | 205935961 | Promoter (<=1kb) | SLC26A9 | 0 |
| chr1 | 209760026 | 209760215 | Promoter (1-2kb) | TRAF3IP3 | 1528 |
| chr1 | 209805892 | 209806157 | Promoter (<=1kb) | IRF6 | 0 |
| chr1 | 210329643 | 210329890 | Promoter (<=1kb) | HHAT | 343 |
| chr1 | 6468577 | 6468951 | Promoter (2-3kb) | TNFRSF25 | -2382 |
| chr1 | 6485201 | 6485466 | Promoter (<=1kb) | PLEKHG5 | 3 |
| chr1 | 6486595 | 6486878 | Promoter (<=1kb) | PLEKHG5 | -641 |
| chr1 | 212566625 | 212566910 | Promoter (1-2kb) | ATF3 | 1291 |
| chr1 | 212700197 | 212700548 | Promoter (<=1kb) | BATF3 | -212 |
| chr1 | 213989580 | 213989826 | Promoter (1-2kb) | PROX1 | 1063 |
| chr1 | 217135217 | 217135394 | Promoter (2-3kb) | ESRRG | 2360 |
| chr1 | 223134851 | 223135026 | Promoter (2-3kb) | TLR5 | 2261 |
| chr1 | 226900403 | 226900720 | Promoter (2-3kb) | COQ8A | 2867 |
| chr1 | 227815457 | 227815753 | Promoter (<=1kb) | PRSS38 | 0 |
| chr1 | 228414477 | 228414734 | Promoter (1-2kb) | TRIM17 | 1295 |
| chr1 | 229433961 | 229434457 | Promoter (<=1kb) | ACTA1 | 0 |
| chr1 | 233905187 | 233905431 | Promoter (<=1kb) | SLC35F3 | 254 |
| chr1 | 8318120 | 8318359 | Promoter (<=1kb) | SLC45A1 | 294 |
| chr1 | 236064810 | 236065418 | Promoter (<=1kb) | NID1 | 0 |
| chr1 | 240093314 | 240093549 | Promoter (1-2kb) | FMN2 | 1434 |
| chr1 | 246724283 | 246724560 | Promoter (<=1kb) | SCCPDH | 236 |
| chr1 | 247169286 | 247169537 | Promoter (2-3kb) | ZNF124 | 2430 |
| chr1 | 247518226 | 247518806 | Promoter (<=1kb) | GCSAML | 0 |
| chr1 | 247530906 | 247531094 | Promoter (1-2kb) | GCSAML-AS1 | 1519 |
| chr1 | 11045309 | 11045528 | Promoter (1-2kb) | MASP2 | 1695 |
| chr1 | 11692719 | 11692972 | Promoter (<=1kb) | DRAXIN | 990 |
| chr1 | 11761735 | 11761911 | Promoter (<=1kb) | C1orf167 | 0 |
| chr1 | 14923207 | 14923521 | Promoter (<=1kb) | KAZN | -609 |
| chr1 | 1246454 | 1246720 | Promoter (<=1kb) | C1QTNF12 | 2 |
| chr1 | 17375041 | 17375218 | Promoter (2-3kb) | PADI6 | 2845 |
| chr10 | 3067160 | 3067395 | Promoter (<=1kb) | PFKP | -125 |
| chr10 | 11018132 | 11018572 | Promoter (<=1kb) | CELF2 | 166 |
| chr10 | 12349266 | 12349624 | Promoter (<=1kb) | CAMK1D | 0 |
| chr10 | 13082275 | 13082497 | Promoter (<=1kb) | OPTN | -674 |
| chr10 | 21174436 | 21174637 | Promoter (<=1kb) | NEBL | -249 |
| chr10 | 22593023 | 22593200 | Promoter (1-2kb) | PIP4K2A | -1310 |
| chr10 | 26217503 | 26218056 | Promoter (<=1kb) | GAD2 | 358 |
| chr10 | 27998853 | 27999051 | Promoter (<=1kb) | ARMC4 | 0 |
| chr10 | 30981209 | 30981583 | Promoter (2-3kb) | ZNF438 | 2843 |
| chr10 | 32938535 | 32939002 | Promoter (2-3kb) | ITGB1 | -2977 |
| chr10 | 43206677 | 43207086 | Promoter (2-3kb) | RASGEF1A | 2098 |
| chr10 | 44386034 | 44386359 | Promoter (<=1kb) | CXCL12 | -937 |
| chr10 | 45373427 | 45373809 | Promoter (<=1kb) | ALOX5 | -367 |
| chr10 | 45847188 | 45847554 | Promoter (<=1kb) | AGAP4 | 0 |
| chr10 | 48493445 | 48493707 | Promoter (<=1kb) | ARHGAP22 | 0 |
| chr10 | 49615218 | 49615393 | Promoter (1-2kb) | CHAT | 1181 |
| chr10 | 51074200 | 51074377 | Promoter (<=1kb) | PRKG1 | 0 |
| chr10 | 69313005 | 69313261 | Promoter (2-3kb) | HK1 | -2524 |
| chr10 | 70380852 | 70381045 | Promoter (<=1kb) | LRRC20 | 869 |
| chr10 | 70441291 | 70441684 | Promoter (<=1kb) | NODAL | 0 |
| chr10 | 70672611 | 70672887 | Promoter (<=1kb) | ADAMTS14 | 0 |
| chr10 | 71396534 | 71396742 | Promoter (<=1kb) | CDH23 | -192 |
| chr10 | 71735858 | 71736389 | Promoter (1-2kb) | C10orf105 | 1435 |
| chr10 | 73911036 | 73911327 | Promoter (<=1kb) | PLAU | 0 |
| chr10 | 77637096 | 77637312 | Promoter (<=1kb) | KCNMA1 | 330 |
| chr10 | 77987218 | 77987466 | Promoter (1-2kb) | POLR3A | -1898 |
| chr10 | 80409038 | 80409243 | Promoter (<=1kb) | PRXL2A | 544 |
| chr10 | 87658669 | 87658859 | Promoter (<=1kb) | PAPSS2 | -754 |
| chr10 | 95758591 | 95758821 | Promoter (2-3kb) | ENTPD1 | 2634 |
| chr10 | 95920863 | 95921072 | Promoter (<=1kb) | CC2D2B | -932 |
| chr10 | 96982195 | 96982382 | Promoter (<=1kb) | LCOR | 911 |
| chr10 | 97582341 | 97582789 | Promoter (1-2kb) | HOGA1 | -1554 |
| chr10 | 98266478 | 98266736 | Promoter (1-2kb) | LOXL4 | 1514 |
| chr10 | 98444933 | 98445192 | Promoter (1-2kb) | HPS1 | 1718 |
| chr10 | 98445521 | 98445737 | Promoter (1-2kb) | HPS1 | 1173 |
| chr10 | 100519319 | 100519782 | Promoter (<=1kb) | SEC31B | 52 |
| chr10 | 100983360 | 100983546 | Promoter (<=1kb) | SEMA4G | -765 |
| chr10 | 101843505 | 101843770 | Promoter (<=1kb) | KCNIP2 | 0 |
| chr10 | 102141179 | 102141413 | Promoter (<=1kb) | PPRC1 | -79 |
| chr10 | 102231320 | 102231657 | Promoter (<=1kb) | PITX3 | 426 |
| chr10 | 102834953 | 102835367 | Promoter (2-3kb) | CYP17A1 | 2055 |
| chr10 | 103457405 | 103457652 | Promoter (1-2kb) | CALHM1 | 1236 |
| chr10 | 103490842 | 103491269 | Promoter (2-3kb) | NEURL1 | -2710 |
| chr10 | 113709354 | 113709640 | Promoter (<=1kb) | CASP7 | 9 |
| chr10 | 114239674 | 114240096 | Promoter (<=1kb) | VWA2 | 318 |
| chr10 | 114404260 | 114404466 | Promoter (<=1kb) | AFAP1L2 | 19 |
| chr10 | 116850310 | 116850531 | Promoter (<=1kb) | HSPA12A | -569 |
| chr10 | 117136565 | 117136989 | Promoter (1-2kb) | VAX1 | 1067 |
| chr10 | 117140031 | 117140500 | Promoter (1-2kb) | VAX1 | -1730 |
| chr10 | 119818386 | 119818612 | Promoter (<=1kb) | INPP5F | -104 |
| chr10 | 121531309 | 121531850 | Promoter (<=1kb) | FGFR2 | 0 |
| chr10 | 121597572 | 121598043 | Promoter (<=1kb) | FGFR2 | 41 |
| chr10 | 123148365 | 123148893 | Promoter (<=1kb) | HMX2 | 243 |
| chr10 | 126463065 | 126463391 | Promoter (<=1kb) | C10orf90 | 0 |
| chr10 | 132101959 | 132102262 | Promoter (2-3kb) | JAKMIP3 | -2409 |
| chr10 | 132399812 | 132400112 | Promoter (2-3kb) | PWWP2B | 2644 |
| chr10 | 132787945 | 132788127 | Promoter (1-2kb) | NKX6-2 | -1893 |
| chr10 | 133102521 | 133103058 | Promoter (<=1kb) | ADGRA1 | 275 |
| chr10 | 133237386 | 133237574 | Promoter (<=1kb) | VENTX | 0 |
| chr10 | 133288108 | 133288284 | Promoter (2-3kb) | TUBGCP2 | 2750 |
| chr10 | 133338316 | 133338660 | Promoter (1-2kb) | CALY | -1381 |
| chr10 | 133422005 | 133422229 | Promoter (2-3kb) | SPRN | 2343 |
| chr10 | 133456210 | 133456513 | Promoter (2-3kb) | SCART1 | 2264 |
| chr10 | 133566382 | 133566626 | Promoter (<=1kb) | SYCE1 | -798 |
| chr11 | 49208015 | 49208447 | Promoter (<=1kb) | FOLH1 | 0 |
| chr11 | 57183767 | 57183970 | Promoter (2-3kb) | LRRC55 | 2020 |
| chr11 | 57425365 | 57425590 | Promoter (1-2kb) | SLC43A3 | 1162 |
| chr11 | 60951217 | 60951475 | Promoter (<=1kb) | SLC15A3 | 310 |
| chr11 | 60952118 | 60952288 | Promoter (<=1kb) | SLC15A3 | -333 |
| chr11 | 61294115 | 61294320 | Promoter (1-2kb) | VWCE | 1104 |
| chr11 | 62242177 | 62242390 | Promoter (<=1kb) | SCGB1D2 | 0 |
| chr11 | 62402848 | 62403160 | Promoter (1-2kb) | SCGB1A1 | -1943 |
| chr11 | 62418649 | 62418910 | Promoter (<=1kb) | SCGB1A1 | -125 |
| chr11 | 64300175 | 64300457 | Promoter (<=1kb) | CATSPERZ | 0 |
| chr11 | 64558578 | 64558753 | Promoter (2-3kb) | SLC22A11 | 2622 |
| chr11 | 64711529 | 64711829 | Promoter (2-3kb) | NRXN2 | 2082 |
| chr11 | 65058665 | 65058850 | Promoter (<=1kb) | NAALADL1 | -116 |
| chr11 | 65088088 | 65088461 | Promoter (<=1kb) | TMEM262 | 630 |
| chr11 | 65169904 | 65170353 | Promoter (<=1kb) | SPDYC | 0 |
| chr11 | 65171145 | 65171529 | Promoter (<=1kb) | SPDYC | 991 |
| chr11 | 65872594 | 65872940 | Promoter (<=1kb) | EFEMP2 | 0 |
| chr11 | 65899942 | 65900132 | Promoter (<=1kb) | FOSL1 | 287 |
| chr11 | 66042544 | 66043005 | Promoter (2-3kb) | GAL3ST3 | 2570 |
| chr11 | 66230696 | 66230882 | Promoter (<=1kb) | PACS1 | 316 |
| chr11 | 66232405 | 66233234 | Promoter (2-3kb) | PACS1 | 2025 |
| chr11 | 66294291 | 66294465 | Promoter (2-3kb) | TMEM151A | 2421 |
| chr11 | 66851043 | 66851312 | Promoter (<=1kb) | PC | -702 |
| chr11 | 66870921 | 66871798 | Promoter (<=1kb) | PC | 241 |
| chr11 | 66906477 | 66906654 | Promoter (1-2kb) | PC | 1247 |
| chr11 | 67406677 | 67406931 | Promoter (2-3kb) | TBC1D10C | 2488 |
| chr11 | 67441867 | 67442037 | Promoter (1-2kb) | CORO1B | 1475 |
| chr11 | 67451347 | 67451889 | Promoter (<=1kb) | CABP4 | -526 |
| chr11 | 67523772 | 67523965 | Promoter (<=1kb) | CABP2 | -376 |
| chr11 | 68798386 | 68798821 | Promoter (<=1kb) | CPT1A | 447 |
| chr11 | 69703289 | 69703723 | Promoter (<=1kb) | FGF19 | 919 |
| chr11 | 72243283 | 72243481 | Promoter (<=1kb) | PHOX2A | 695 |
| chr11 | 73218127 | 73218529 | Promoter (<=1kb) | P2RY2 | 0 |
| chr11 | 73264387 | 73264685 | Promoter (<=1kb) | P2RY6 | 0 |
| chr11 | 73343604 | 73343918 | Promoter (<=1kb) | ARHGEF17 | 680 |
| chr11 | 74467078 | 74467531 | Promoter (<=1kb) | KCNE3 | 97 |
| chr11 | 74731143 | 74731758 | Promoter (<=1kb) | CHRDL2 | 0 |
| chr11 | 75098224 | 75098512 | Promoter (1-2kb) | OR2AT4 | -1348 |
| chr11 | 75159430 | 75159666 | Promoter (<=1kb) | SLCO2B1 | -122 |
| chr11 | 75449423 | 75450101 | Promoter (<=1kb) | GDPD5 | 0 |
| chr11 | 75492097 | 75492336 | Promoter (1-2kb) | GDPD5 | -1346 |
| chr11 | 78023127 | 78023297 | Promoter (<=1kb) | KCTD14 | 0 |
| chr11 | 78339535 | 78339733 | Promoter (2-3kb) | GAB2 | 2147 |
| chr11 | 111937592 | 111937975 | Promoter (<=1kb) | DIXDC1 | 389 |
| chr11 | 117796829 | 117797166 | Promoter (<=1kb) | DSCAML1 | 0 |
| chr11 | 117870161 | 117870420 | Promoter (<=1kb) | FXYD6 | -971 |
| chr11 | 118531285 | 118531487 | Promoter (<=1kb) | TMEM25 | 0 |
| chr11 | 119149773 | 119150366 | Promoter (<=1kb) | ABCG4 | 0 |
| chr11 | 119341924 | 119342092 | Promoter (1-2kb) | C1QTNF5 | -1041 |
| chr11 | 121452221 | 121452719 | Promoter (<=1kb) | SORL1 | 18 |
| chr11 | 122984494 | 122984672 | Promoter (2-3kb) | BSX | -2774 |
| chr11 | 128905303 | 128905510 | Promoter (<=1kb) | C11orf45 | 187 |
| chr11 | 198825 | 199063 | Promoter (1-2kb) | ODF3 | 1522 |
| chr11 | 298153 | 298341 | Promoter (1-2kb) | IFITM5 | 1185 |
| chr11 | 315144 | 315685 | Promoter (1-2kb) | IFITM1 | 1291 |
| chr11 | 416427 | 416883 | Promoter (<=1kb) | SIGIRR | 442 |
| chr11 | 626372 | 627017 | Promoter (<=1kb) | SCT | 126 |
| chr11 | 787385 | 787761 | Promoter (2-3kb) | CEND1 | 2362 |
| chr11 | 800095 | 800458 | Promoter (1-2kb) | SLC25A22 | -1762 |
| chr11 | 1099209 | 1099617 | Promoter (2-3kb) | MUC2 | -2838 |
| chr11 | 1411409 | 1411677 | Promoter (<=1kb) | BRSK2 | 257 |
| chr11 | 1694994 | 1695388 | Promoter (1-2kb) | KRTAP5-6 | -1807 |
| chr11 | 1841235 | 1841404 | Promoter (1-2kb) | TNNI2 | 1041 |
| chr11 | 1855970 | 1856387 | Promoter (2-3kb) | LSP1 | 3000 |
| chr11 | 1872695 | 1872946 | Promoter (2-3kb) | LSP1 | 2443 |
| chr11 | 1916940 | 1917226 | Promoter (2-3kb) | TNNT3 | -2336 |
| chr11 | 2460714 | 2461033 | Promoter (<=1kb) | KCNQ1 | -403 |
| chr11 | 3218860 | 3219129 | Promoter (<=1kb) | MRGPRG | -47 |
| chr11 | 7673224 | 7673562 | Promoter (<=1kb) | CYB5R2 | 0 |
| chr11 | 8710703 | 8710893 | Promoter (2-3kb) | ST5 | -2622 |
| chr11 | 9003618 | 9003827 | Promoter (<=1kb) | NRIP3 | 222 |
| chr11 | 9091683 | 9091851 | Promoter (<=1kb) | SCUBE2 | -82 |
| chr11 | 10463683 | 10463949 | Promoter (2-3kb) | AMPD3 | 2543 |
| chr11 | 20363778 | 20363958 | Promoter (<=1kb) | HTATIP2 | 0 |
| chr11 | 20601306 | 20601596 | Promoter (1-2kb) | SLC6A5 | 1906 |
| chr11 | 20669748 | 20670023 | Promoter (<=1kb) | NELL1 | 87 |
| chr11 | 27700656 | 27701055 | Promoter (<=1kb) | BDNF | 0 |
| chr11 | 30016994 | 30017242 | Promoter (<=1kb) | KCNA4 | 0 |
| chr11 | 31805557 | 31805737 | Promoter (<=1kb) | PAX6 | 674 |
| chr11 | 32829646 | 32830016 | Promoter (<=1kb) | PRRG4 | 0 |
| chr11 | 35619560 | 35619768 | Promoter (1-2kb) | FJX1 | 1141 |
| chr11 | 46277847 | 46278174 | Promoter (<=1kb) | CREB3L1 | 186 |
| chr12 | 213795 | 214268 | Promoter (<=1kb) | SLC6A12 | 0 |
| chr12 | 1573405 | 1573606 | Promoter (1-2kb) | WNT5B | -1051 |
| chr12 | 1915276 | 1915816 | Promoter (<=1kb) | CACNA2D4 | -49 |
| chr12 | 6792338 | 6792531 | Promoter (2-3kb) | CD4 | 2802 |
| chr12 | 12047865 | 12048213 | Promoter (1-2kb) | BCL2L14 | -1631 |
| chr12 | 12812636 | 12812848 | Promoter (<=1kb) | DDX47 | -468 |
| chr12 | 13101292 | 13101546 | Promoter (2-3kb) | GSG1 | 2099 |
| chr12 | 21527559 | 21527926 | Promoter (<=1kb) | SPX | 515 |
| chr12 | 22334567 | 22334905 | Promoter (<=1kb) | ST8SIA1 | 0 |
| chr12 | 24901962 | 24902163 | Promoter (<=1kb) | BCAT1 | 133 |
| chr12 | 47080127 | 47080437 | Promoter (<=1kb) | AMIGO2 | -176 |
| chr12 | 47942652 | 47942845 | Promoter (<=1kb) | VDR | 203 |
| chr12 | 48350157 | 48350335 | Promoter (<=1kb) | ZNF641 | 436 |
| chr12 | 48864609 | 48865023 | Promoter (<=1kb) | RND1 | 875 |
| chr12 | 48904434 | 48904667 | Promoter (<=1kb) | CCDC65 | 285 |
| chr12 | 49294375 | 49294774 | Promoter (<=1kb) | PRPH | 673 |
| chr12 | 49630897 | 49631188 | Promoter (<=1kb) | PRPF40B | 353 |
| chr12 | 49975542 | 49975766 | Promoter (2-3kb) | AQP6 | 2368 |
| chr12 | 51270864 | 51271071 | Promoter (<=1kb) | SMAGP | 0 |
| chr12 | 51323947 | 51324247 | Promoter (<=1kb) | BIN2 | 0 |
| chr12 | 51391303 | 51391711 | Promoter (<=1kb) | GALNT6 | 0 |
| chr12 | 51907545 | 51907867 | Promoter (<=1kb) | ACVRL1 | 127 |
| chr12 | 52905072 | 52905287 | Promoter (1-2kb) | KRT8 | 1331 |
| chr12 | 53104871 | 53105195 | Promoter (1-2kb) | SOAT2 | 1353 |
| chr12 | 53241861 | 53242213 | Promoter (<=1kb) | AC021072.1 | 0 |
| chr12 | 53424981 | 53425481 | Promoter (1-2kb) | AMHR2 | 1046 |
| chr12 | 54053387 | 54053673 | Promoter (<=1kb) | HOXC4 | -204 |
| chr12 | 55707733 | 55708148 | Promoter (<=1kb) | ITGA7 | 0 |
| chr12 | 56020824 | 56021032 | Promoter (<=1kb) | IKZF4 | -48 |
| chr12 | 56995438 | 56995648 | Promoter (<=1kb) | GPR182 | 992 |
| chr12 | 57129489 | 57129671 | Promoter (<=1kb) | STAT6 | -389 |
| chr12 | 57549999 | 57550210 | Promoter (<=1kb) | KIF5A | 0 |
| chr12 | 57631911 | 57632156 | Promoter (<=1kb) | B4GALNT1 | 491 |
| chr12 | 70367151 | 70367438 | Promoter (<=1kb) | KCNMB4 | 875 |
| chr12 | 75209572 | 75209743 | Promoter (<=1kb) | KCNC2 | 0 |
| chr12 | 90954778 | 90954992 | Promoter (<=1kb) | CCER1 | 184 |
| chr12 | 95790301 | 95790877 | Promoter (<=1kb) | NTN4 | 0 |
| chr12 | 108623089 | 108623258 | Promoter (1-2kb) | SELPLG | 1072 |
| chr12 | 108879541 | 108879918 | Promoter (<=1kb) | DAO | -112 |
| chr12 | 109309881 | 109310182 | Promoter (<=1kb) | FOXN4 | -661 |
| chr12 | 109833639 | 109833845 | Promoter (<=1kb) | TRPV4 | -238 |
| chr12 | 110687641 | 110687863 | Promoter (<=1kb) | HVCN1 | 846 |
| chr12 | 112576085 | 112576262 | Promoter (<=1kb) | RPH3A | 988 |
| chr12 | 113135671 | 113136032 | Promoter (<=1kb) | RASAL1 | 0 |
| chr12 | 113186745 | 113186947 | Promoter (1-2kb) | RITA1 | 1203 |
| chr12 | 120654921 | 120655328 | Promoter (<=1kb) | CABP1 | 0 |
| chr12 | 120711592 | 120711810 | Promoter (1-2kb) | UNC119B | 1157 |
| chr12 | 120978773 | 120979205 | Promoter (<=1kb) | HNF1A | 24 |
| chr12 | 121273763 | 121274339 | Promoter (<=1kb) | CAMKK2 | 192 |
| chr12 | 121535827 | 121536027 | Promoter (1-2kb) | KDM2B | 1589 |
| chr12 | 121578388 | 121578776 | Promoter (1-2kb) | KDM2B | 1683 |
| chr12 | 121750509 | 121751368 | Promoter (<=1kb) | TMEM120B | -761 |
| chr12 | 121860451 | 121860731 | Promoter (1-2kb) | HPD | -1592 |
| chr12 | 121916878 | 121917141 | Promoter (<=1kb) | WDR66 | -721 |
| chr12 | 121918465 | 121918797 | Promoter (<=1kb) | WDR66 | 0 |
| chr12 | 122131177 | 122131400 | Promoter (1-2kb) | MLXIP | -1125 |
| chr12 | 122133574 | 122133749 | Promoter (<=1kb) | MLXIP | 276 |
| chr12 | 122980709 | 122980903 | Promoter (<=1kb) | ARL6IP4 | 0 |
| chr12 | 123384666 | 123384884 | Promoter (<=1kb) | KMT5A | 893 |
| chr12 | 124294914 | 124295152 | Promoter (<=1kb) | RFLNA | -74 |
| chr12 | 124314488 | 124315183 | Promoter (2-3kb) | RFLNA | 2780 |
| chr12 | 124422361 | 124422656 | Promoter (<=1kb) | NCOR2 | 0 |
| chr12 | 124820415 | 124820663 | Promoter (2-3kb) | SCARB1 | -2708 |
| chr12 | 124980217 | 124980584 | Promoter (<=1kb) | DHX37 | 114 |
| chr12 | 130715456 | 130715711 | Promoter (<=1kb) | RIMBP2 | 570 |
| chr12 | 131844864 | 131845170 | Promoter (<=1kb) | MMP17 | -125 |
| chr12 | 131960221 | 131960730 | Promoter (<=1kb) | EP400 | 0 |
| chr12 | 132620569 | 132620808 | Promoter (1-2kb) | P2RX2 | 1752 |
| chr12 | 132716831 | 132717022 | Promoter (2-3kb) | PGAM5 | 2078 |
| chr12 | 132717620 | 132717973 | Promoter (2-3kb) | PGAM5 | 2867 |
| chr12 | 133235690 | 133236240 | Promoter (<=1kb) | ANHX | 0 |
| chr13 | 20232072 | 20232679 | Promoter (<=1kb) | GJB6 | 0 |
| chr13 | 20526879 | 20527063 | Promoter (1-2kb) | CRYL1 | -1006 |
| chr13 | 25169963 | 25170426 | Promoter (1-2kb) | AMER2 | 1861 |
| chr13 | 25171583 | 25172109 | Promoter (<=1kb) | AMER2 | 178 |
| chr13 | 26758534 | 26758987 | Promoter (1-2kb) | GPR12 | 1303 |
| chr13 | 26759419 | 26759596 | Promoter (<=1kb) | GPR12 | 694 |
| chr13 | 26760544 | 26760907 | Promoter (<=1kb) | GPR12 | 0 |
| chr13 | 27968363 | 27968763 | Promoter (2-3kb) | CDX2 | 2376 |
| chr13 | 28309484 | 28309682 | Promoter (1-2kb) | FLT1 | -1050 |
| chr13 | 28494108 | 28494415 | Promoter (<=1kb) | FLT1 | 664 |
| chr13 | 30906586 | 30906794 | Promoter (<=1kb) | MEDAG | 395 |
| chr13 | 33350511 | 33350714 | Promoter (<=1kb) | STARD13 | 0 |
| chr13 | 44373257 | 44374308 | Promoter (<=1kb) | SERP2 | 0 |
| chr13 | 46386972 | 46387230 | Promoter (<=1kb) | RUBCNL | 3 |
| chr13 | 49496095 | 49496645 | Promoter (<=1kb) | PHF11 | 0 |
| chr13 | 49630512 | 49630986 | Promoter (2-3kb) | ARL11 | 2213 |
| chr13 | 52739582 | 52739805 | Promoter (<=1kb) | CNMD | 0 |
| chr13 | 75617654 | 75617918 | Promoter (2-3kb) | LMO7 | -2516 |
| chr13 | 77698682 | 77698945 | Promoter (<=1kb) | SLAIN1 | 0 |
| chr13 | 94478953 | 94479162 | Promoter (<=1kb) | DCT | 507 |
| chr13 | 98752036 | 98752553 | Promoter (<=1kb) | SLC15A1 | 101 |
| chr13 | 98978119 | 98978299 | Promoter (<=1kb) | DOCK9 | -35 |
| chr13 | 100532303 | 100532594 | Promoter (1-2kb) | GGACT | 1208 |
| chr13 | 111114660 | 111114862 | Promoter (<=1kb) | ARHGEF7 | 41 |
| chr13 | 113103552 | 113103920 | Promoter (1-2kb) | F7 | -1868 |
| chr13 | 113657976 | 113658182 | Promoter (<=1kb) | ATP4B | 4 |
| chr14 | 20506033 | 20506453 | Promoter (<=1kb) | RNASE10 | 496 |
| chr14 | 23378340 | 23378535 | Promoter (1-2kb) | CMTM5 | 1088 |
| chr14 | 23988731 | 23988899 | Promoter (<=1kb) | DHRS4L2 | 0 |
| chr14 | 24207037 | 24207225 | Promoter (1-2kb) | TSSK4 | 1179 |
| chr14 | 24311320 | 24311530 | Promoter (<=1kb) | CIDEB | 0 |
| chr14 | 24574186 | 24574584 | Promoter (1-2kb) | CTSG | 1676 |
| chr14 | 28768109 | 28768284 | Promoter (2-3kb) | FOXG1 | 2721 |
| chr14 | 47674516 | 47674775 | Promoter (<=1kb) | MDGA2 | 0 |
| chr14 | 50822046 | 50822282 | Promoter (<=1kb) | NIN | 0 |
| chr14 | 50944016 | 50944240 | Promoter (<=1kb) | PYGL | 216 |
| chr14 | 50947679 | 50947954 | Promoter (2-3kb) | PYGL | -2943 |
| chr14 | 51093772 | 51093952 | Promoter (1-2kb) | TRIM9 | 1124 |
| chr14 | 54443138 | 54443683 | Promoter (1-2kb) | CNIH1 | -1707 |
| chr14 | 59464168 | 59464641 | Promoter (<=1kb) | GPR135 | 701 |
| chr14 | 59465201 | 59465453 | Promoter (<=1kb) | GPR135 | 0 |
| chr14 | 61280279 | 61280722 | Promoter (1-2kb) | TMEM30B | 1118 |
| chr14 | 61281229 | 61281490 | Promoter (<=1kb) | TMEM30B | 350 |
| chr14 | 64059588 | 64060174 | Promoter (2-3kb) | SYNE2 | -2608 |
| chr14 | 64294191 | 64294425 | Promoter (<=1kb) | ESR2 | 0 |
| chr14 | 64880126 | 64880870 | Promoter (<=1kb) | SPTB | -219 |
| chr14 | 69573019 | 69573257 | Promoter (1-2kb) | CCDC177 | 1580 |
| chr14 | 73237400 | 73237598 | Promoter (<=1kb) | PAPLN | 0 |
| chr14 | 73237851 | 73238182 | Promoter (<=1kb) | PAPLN | 354 |
| chr14 | 74349159 | 74349458 | Promoter (<=1kb) | VRTN | 746 |
| chr14 | 74611414 | 74611746 | Promoter (<=1kb) | LTBP2 | 326 |
| chr14 | 76373926 | 76374096 | Promoter (2-3kb) | ESRRB | -2041 |
| chr14 | 91254060 | 91254330 | Promoter (<=1kb) | GPR68 | -135 |
| chr14 | 93788322 | 93789054 | Promoter (<=1kb) | PRIMA1 | 0 |
| chr14 | 94099059 | 94099243 | Promoter (2-3kb) | IFI27L1 | 2152 |
| chr14 | 94767640 | 94768016 | Promoter (2-3kb) | GSC | 2214 |
| chr14 | 96254696 | 96254864 | Promoter (<=1kb) | BDKRB1 | -960 |
| chr14 | 96261932 | 96262138 | Promoter (1-2kb) | BDKRB1 | -1532 |
| chr14 | 99272956 | 99273248 | Promoter (1-2kb) | BCL11B | -1432 |
| chr14 | 99645860 | 99646066 | Promoter (<=1kb) | HHIPL1 | 717 |
| chr14 | 104576865 | 104577360 | Promoter (2-3kb) | C14orf180 | -2324 |
| chr14 | 104593110 | 104593296 | Promoter (<=1kb) | TMEM179 | -601 |
| chr14 | 104604635 | 104604893 | Promoter (<=1kb) | TMEM179 | 0 |
| chr14 | 104934027 | 104934211 | Promoter (1-2kb) | PLD4 | 1867 |
| chr14 | 104956587 | 104956768 | Promoter (2-3kb) | AHNAK2 | -2683 |
| chr14 | 105247805 | 105248016 | Promoter (<=1kb) | BRF1 | 0 |
| chr14 | 105312991 | 105313269 | Promoter (1-2kb) | PACS2 | -1146 |
| chr15 | 22917883 | 22918209 | Promoter (<=1kb) | CYFIP1 | -570 |
| chr15 | 23446453 | 23446861 | Promoter (<=1kb) | GOLGA6L2 | 373 |
| chr15 | 23647185 | 23647679 | Promoter (<=1kb) | MAGEL2 | 167 |
| chr15 | 26772341 | 26773071 | Promoter (<=1kb) | GABRB3 | 3 |
| chr15 | 26866828 | 26867417 | Promoter (<=1kb) | GABRA5 | 0 |
| chr15 | 26970959 | 26971705 | Promoter (<=1kb) | GABRG3 | 0 |
| chr15 | 28849564 | 28849791 | Promoter (<=1kb) | GOLGA6L7 | -889 |
| chr15 | 29570245 | 29570630 | Promoter (<=1kb) | FAM189A1 | 93 |
| chr15 | 29571157 | 29571465 | Promoter (<=1kb) | FAM189A1 | -434 |
| chr15 | 31161155 | 31161360 | Promoter (<=1kb) | TRPM1 | 0 |
| chr15 | 31163320 | 31163646 | Promoter (2-3kb) | TRPM1 | -2047 |
| chr15 | 40283204 | 40283433 | Promoter (<=1kb) | ANKRD63 | -618 |
| chr15 | 40307780 | 40308093 | Promoter (<=1kb) | PLCB2 | 0 |
| chr15 | 40926410 | 40926965 | Promoter (2-3kb) | DLL4 | -2368 |
| chr15 | 41511287 | 41511964 | Promoter (1-2kb) | LTK | 1832 |
| chr15 | 41660320 | 41660882 | Promoter (<=1kb) | MGA | 0 |
| chr15 | 41836754 | 41836930 | Promoter (<=1kb) | PLA2G4B | -845 |
| chr15 | 43133877 | 43134050 | Promoter (<=1kb) | TMEM62 | 353 |
| chr15 | 43218950 | 43219163 | Promoter (1-2kb) | EPB42 | 1846 |
| chr15 | 43648823 | 43649361 | Promoter (<=1kb) | CATSPER2 | 0 |
| chr15 | 52789085 | 52789359 | Promoter (<=1kb) | ONECUT1 | 653 |
| chr15 | 56243060 | 56243248 | Promoter (<=1kb) | RFX7 | 18 |
| chr15 | 57376642 | 57376904 | Promoter (<=1kb) | CGNL1 | 137 |
| chr15 | 62068104 | 62068507 | Promoter (1-2kb) | C2CD4A | 1127 |
| chr15 | 64152150 | 64152482 | Promoter (<=1kb) | SNX22 | 433 |
| chr15 | 64912352 | 64912569 | Promoter (<=1kb) | ANKDD1A | 434 |
| chr15 | 65067693 | 65068087 | Promoter (<=1kb) | RASL12 | 0 |
| chr15 | 65077871 | 65078119 | Promoter (1-2kb) | KBTBD13 | 1055 |
| chr15 | 66128924 | 66129262 | Promoter (<=1kb) | MEGF11 | -521 |
| chr15 | 67066074 | 67066262 | Promoter (<=1kb) | SMAD3 | 229 |
| chr15 | 74365689 | 74366038 | Promoter (<=1kb) | CYP11A1 | 168 |
| chr15 | 75359557 | 75359839 | Promoter (<=1kb) | MAN2C1 | 0 |
| chr15 | 75360831 | 75361042 | Promoter (1-2kb) | MAN2C1 | -1151 |
| chr15 | 75712854 | 75713128 | Promoter (<=1kb) | CSPG4 | -6 |
| chr15 | 76995253 | 76995626 | Promoter (<=1kb) | PSTPIP1 | 0 |
| chr15 | 77696822 | 77697108 | Promoter (<=1kb) | LINGO1 | -674 |
| chr15 | 78264781 | 78265067 | Promoter (<=1kb) | DNAJA4 | 207 |
| chr15 | 78944705 | 78945330 | Promoter (<=1kb) | CTSH | 0 |
| chr15 | 79091901 | 79092282 | Promoter (1-2kb) | RASGRF1 | -1128 |
| chr15 | 81299758 | 81300202 | Promoter (2-3kb) | IL16 | 2845 |
| chr15 | 82432694 | 82432929 | Promoter (2-3kb) | GOLGA6L9 | 2674 |
| chr15 | 82698745 | 82699159 | Promoter (<=1kb) | AP3B2 | 716 |
| chr15 | 83108582 | 83108882 | Promoter (<=1kb) | TM6SF1 | 901 |
| chr15 | 88894908 | 88895076 | Promoter (<=1kb) | HAPLN3 | 521 |
| chr15 | 100544277 | 100544908 | Promoter (<=1kb) | CERS3 | 0 |
| chr15 | 100879900 | 100880261 | Promoter (<=1kb) | ALDH1A3 | 25 |
| chr15 | 101490210 | 101490462 | Promoter (<=1kb) | PCSK6 | -226 |
| chr16 | 152436 | 152634 | Promoter (<=1kb) | HBZ | -53 |
| chr16 | 166208 | 166612 | Promoter (<=1kb) | HBM | 230 |
| chr16 | 371461 | 371641 | Promoter (<=1kb) | MRPL28 | -172 |
| chr16 | 558732 | 558958 | Promoter (1-2kb) | PRR35 | -1464 |
| chr16 | 630483 | 630773 | Promoter (<=1kb) | WFIKKN1 | -159 |
| chr16 | 762035 | 762669 | Promoter (<=1kb) | MSLN | 946 |
| chr16 | 982691 | 982885 | Promoter (<=1kb) | SOX8 | 883 |
| chr16 | 1080260 | 1080797 | Promoter (1-2kb) | SSTR5 | 1479 |
| chr16 | 1254967 | 1255199 | Promoter (<=1kb) | TPSD1 | -860 |
| chr16 | 1348747 | 1349060 | Promoter (2-3kb) | TSR3 | 2851 |
| chr16 | 1487567 | 1488240 | Promoter (<=1kb) | PTX4 | 227 |
| chr16 | 1632353 | 1632789 | Promoter (<=1kb) | AL031708.1 | 94 |
| chr16 | 1792339 | 1792546 | Promoter (1-2kb) | IGFALS | 1186 |
| chr16 | 1794548 | 1794765 | Promoter (<=1kb) | IGFALS | 206 |
| chr16 | 1918439 | 1918631 | Promoter (<=1kb) | HS3ST6 | 0 |
| chr16 | 1958494 | 1958711 | Promoter (<=1kb) | NDUFB10 | -797 |
| chr16 | 2019987 | 2020171 | Promoter (<=1kb) | NPW | 85 |
| chr16 | 2472172 | 2472684 | Promoter (<=1kb) | NTN3 | 673 |
| chr16 | 2818299 | 2818469 | Promoter (1-2kb) | PRSS21 | 1072 |
| chr16 | 2859832 | 2860175 | Promoter (1-2kb) | PRSS22 | -1662 |
| chr16 | 3065125 | 3065338 | Promoter (<=1kb) | IL32 | 0 |
| chr16 | 3493701 | 3494067 | Promoter (1-2kb) | C16orf90 | 1357 |
| chr16 | 3495275 | 3495489 | Promoter (<=1kb) | C16orf90 | 0 |
| chr16 | 3641321 | 3641491 | Promoter (1-2kb) | DNASE1 | -1447 |
| chr16 | 11281095 | 11281289 | Promoter (<=1kb) | PRM1 | 61 |
| chr16 | 11670043 | 11670367 | Promoter (1-2kb) | SNN | 1629 |
| chr16 | 14935347 | 14935530 | Promoter (1-2kb) | NPIPA1 | -1913 |
| chr16 | 15949086 | 15949825 | Promoter (<=1kb) | ABCC1 | 0 |
| chr16 | 21159429 | 21159665 | Promoter (<=1kb) | DNAH3 | 0 |
| chr16 | 21705634 | 21705810 | Promoter (<=1kb) | OTOA | 671 |
| chr16 | 22814709 | 22815103 | Promoter (<=1kb) | HS3ST2 | 532 |
| chr16 | 28609741 | 28609946 | Promoter (<=1kb) | SULT1A1 | 61 |
| chr16 | 29485415 | 29485604 | Promoter (<=1kb) | NPIPB12 | 255 |
| chr16 | 29799406 | 29799588 | Promoter (2-3kb) | KIF22 | 2520 |
| chr16 | 30245118 | 30245298 | Promoter (<=1kb) | NPIPB13 | 503 |
| chr16 | 30473891 | 30474467 | Promoter (1-2kb) | ITGAL | 1140 |
| chr16 | 30738000 | 30738187 | Promoter (2-3kb) | TMEM265 | -2194 |
| chr16 | 31215047 | 31215294 | Promoter (<=1kb) | TRIM72 | 956 |
| chr16 | 31355143 | 31355360 | Promoter (<=1kb) | ITGAX | 0 |
| chr16 | 48231952 | 48232181 | Promoter (<=1kb) | ABCC11 | 90 |
| chr16 | 49780475 | 49780732 | Promoter (<=1kb) | ZNF423 | 0 |
| chr16 | 49858378 | 49858716 | Promoter (<=1kb) | ZNF423 | -459 |
| chr16 | 56589373 | 56589654 | Promoter (<=1kb) | MT3 | 0 |
| chr16 | 56626141 | 56626336 | Promoter (<=1kb) | MT1E | 343 |
| chr16 | 56651911 | 56652318 | Promoter (<=1kb) | MT1B | 0 |
| chr16 | 56657639 | 56657893 | Promoter (<=1kb) | MT1F | 0 |
| chr16 | 57283543 | 57283776 | Promoter (<=1kb) | PLLP | 885 |
| chr16 | 57622721 | 57622931 | Promoter (2-3kb) | ADGRG1 | 2644 |
| chr16 | 57667321 | 57667607 | Promoter (<=1kb) | ADGRG3 | -580 |
| chr16 | 57781298 | 57781587 | Promoter (<=1kb) | KIFC3 | 710 |
| chr16 | 65122660 | 65122863 | Promoter (<=1kb) | CDH11 | -462 |
| chr16 | 66426190 | 66426476 | Promoter (<=1kb) | BEAN1 | -821 |
| chr16 | 67150740 | 67150920 | Promoter (<=1kb) | B3GNT9 | 294 |
| chr16 | 67163264 | 67163559 | Promoter (<=1kb) | AC074143.1 | -149 |
| chr16 | 67394826 | 67394995 | Promoter (1-2kb) | TPPP3 | -1291 |
| chr16 | 67646789 | 67647242 | Promoter (1-2kb) | CARMIL2 | 1542 |
| chr16 | 67943488 | 67943876 | Promoter (<=1kb) | LCAT | 255 |
| chr16 | 67961780 | 67962015 | Promoter (2-3kb) | SLC12A4 | 2050 |
| chr16 | 68737517 | 68737777 | Promoter (<=1kb) | CDH1 | 165 |
| chr16 | 69119716 | 69120069 | Promoter (<=1kb) | CHTF8 | 501 |
| chr16 | 70654309 | 70654537 | Promoter (<=1kb) | IL34 | 210 |
| chr16 | 71625576 | 71626132 | Promoter (<=1kb) | MARVELD3 | -29 |
| chr16 | 72125299 | 72125579 | Promoter (<=1kb) | PMFBP1 | -446 |
| chr16 | 75206973 | 75207291 | Promoter (<=1kb) | CTRB2 | 0 |
| chr16 | 83969914 | 83970177 | Promoter (1-2kb) | NECAB2 | -1274 |
| chr16 | 84368488 | 84368833 | Promoter (<=1kb) | ATP2C2 | 0 |
| chr16 | 84819659 | 84820342 | Promoter (<=1kb) | CRISPLD2 | 0 |
| chr16 | 85708686 | 85708885 | Promoter (1-2kb) | C16orf74 | 1965 |
| chr16 | 87861455 | 87861691 | Promoter (1-2kb) | SLC7A5 | 1948 |
| chr16 | 87936701 | 87936870 | Promoter (<=1kb) | CA5A | -121 |
| chr16 | 87959462 | 87959807 | Promoter (<=1kb) | BANP | -64 |
| chr16 | 88425083 | 88425431 | Promoter (2-3kb) | ZNF469 | -2040 |
| chr16 | 88427903 | 88428077 | Promoter (<=1kb) | ZNF469 | 432 |
| chr16 | 88865723 | 88866134 | Promoter (<=1kb) | PABPN1L | 484 |
| chr16 | 88941057 | 88941230 | Promoter (<=1kb) | CBFA2T3 | 0 |
| chr16 | 89949513 | 89949906 | Promoter (<=1kb) | DEF8 | 710 |
| chr17 | 447714 | 448176 | Promoter (1-2kb) | RFLNB | -1775 |
| chr17 | 1279610 | 1279820 | Promoter (<=1kb) | TRARG1 | 0 |
| chr17 | 1564306 | 1564532 | Promoter (1-2kb) | PITPNA | -1490 |
| chr17 | 2755999 | 2756422 | Promoter (<=1kb) | RAP1GAP2 | 294 |
| chr17 | 3557818 | 3558052 | Promoter (<=1kb) | TRPV3 | 0 |
| chr17 | 3609528 | 3609762 | Promoter (<=1kb) | TRPV1 | -117 |
| chr17 | 3918748 | 3919118 | Promoter (2-3kb) | P2RX1 | -2248 |
| chr17 | 3964250 | 3964533 | Promoter (<=1kb) | ATP2A3 | 0 |
| chr17 | 4807874 | 4808071 | Promoter (<=1kb) | PLD2 | 705 |
| chr17 | 5123494 | 5123697 | Promoter (<=1kb) | ZNF232 | -392 |
| chr17 | 6555466 | 6555675 | Promoter (<=1kb) | PITPNM3 | 785 |
| chr17 | 7178133 | 7178309 | Promoter (<=1kb) | ASGR1 | -17 |
| chr17 | 7294530 | 7294944 | Promoter (<=1kb) | YBX2 | 0 |
| chr17 | 7676526 | 7676717 | Promoter (<=1kb) | TP53 | 0 |
| chr17 | 8003453 | 8004041 | Promoter (<=1kb) | GUCY2D | 859 |
| chr17 | 8744981 | 8745264 | Promoter (<=1kb) | CCDC42 | -145 |
| chr17 | 8865326 | 8865717 | Promoter (1-2kb) | PIK3R6 | 1960 |
| chr17 | 8912620 | 8912937 | Promoter (<=1kb) | PIK3R5 | -103 |
| chr17 | 17584825 | 17585011 | Promoter (2-3kb) | PEMT | -2394 |
| chr17 | 18362760 | 18362978 | Promoter (<=1kb) | SHMT1 | 473 |
| chr17 | 19385021 | 19385247 | Promoter (1-2kb) | MFAP4 | 1685 |
| chr17 | 29004861 | 29005236 | Promoter (<=1kb) | SEZ6 | 677 |
| chr17 | 29079304 | 29079681 | Promoter (<=1kb) | TIAF1 | -447 |
| chr17 | 29761191 | 29761581 | Promoter (<=1kb) | SSH2 | 0 |
| chr17 | 36388318 | 36388635 | Promoter (<=1kb) | TBC1D3H | 0 |
| chr17 | 38343507 | 38343707 | Promoter (<=1kb) | GPR179 | 103 |
| chr17 | 39223091 | 39224074 | Promoter (1-2kb) | STAC2 | 1798 |
| chr17 | 39696835 | 39697038 | Promoter (1-2kb) | ERBB2 | 1904 |
| chr17 | 39744532 | 39744785 | Promoter (2-3kb) | GRB7 | 2280 |
| chr17 | 39868268 | 39868492 | Promoter (<=1kb) | ZPBP2 | 52 |
| chr17 | 39962687 | 39963069 | Promoter (<=1kb) | GSDMA | 0 |
| chr17 | 40363615 | 40363941 | Promoter (<=1kb) | GJD3 | 0 |
| chr17 | 40501717 | 40502078 | Promoter (<=1kb) | TNS4 | -120 |
| chr17 | 41527609 | 41527985 | Promoter (<=1kb) | KRT19 | 323 |
| chr17 | 42039668 | 42039894 | Promoter (1-2kb) | ZNF385C | -1642 |
| chr17 | 44172040 | 44172225 | Promoter (1-2kb) | ASB16 | 1334 |
| chr17 | 44656456 | 44657056 | Promoter (<=1kb) | MEIOC | 0 |
| chr17 | 47696928 | 47697112 | Promoter (1-2kb) | TBKBP1 | 1243 |
| chr17 | 47734740 | 47735158 | Promoter (1-2kb) | TBX21 | 1496 |
| chr17 | 48429506 | 48429746 | Promoter (<=1kb) | SKAP1 | 444 |
| chr17 | 48430197 | 48430554 | Promoter (<=1kb) | SKAP1 | 0 |
| chr17 | 49133003 | 49133226 | Promoter (<=1kb) | B4GALNT2 | 36 |
| chr17 | 50469268 | 50469596 | Promoter (<=1kb) | CHAD | -302 |
| chr17 | 50765845 | 50766052 | Promoter (<=1kb) | ANKRD40CL | 861 |
| chr17 | 55265926 | 55266112 | Promoter (<=1kb) | HLF | -110 |
| chr17 | 57096633 | 57096836 | Promoter (<=1kb) | AKAP1 | 0 |
| chr17 | 58195080 | 58195350 | Promoter (2-3kb) | EPX | 2343 |
| chr17 | 58279246 | 58279566 | Promoter (1-2kb) | MPO | 1369 |
| chr17 | 58279861 | 58280120 | Promoter (<=1kb) | MPO | 815 |
| chr17 | 58487301 | 58487538 | Promoter (<=1kb) | HSF5 | 846 |
| chr17 | 58487801 | 58488025 | Promoter (<=1kb) | HSF5 | 359 |
| chr17 | 62421253 | 62421545 | Promoter (2-3kb) | METTL2A | -2322 |
| chr17 | 62629292 | 62629860 | Promoter (1-2kb) | AC080038.1 | -1702 |
| chr17 | 63486993 | 63487163 | Promoter (2-3kb) | ACE | 2147 |
| chr17 | 63931007 | 63931194 | Promoter (<=1kb) | AC127029.3 | 191 |
| chr17 | 63971677 | 63971898 | Promoter (1-2kb) | SCN4A | 1020 |
| chr17 | 63974151 | 63974683 | Promoter (1-2kb) | SCN4A | -1233 |
| chr17 | 64005534 | 64005745 | Promoter (1-2kb) | ICAM2 | 1187 |
| chr17 | 64920214 | 64921040 | Promoter (<=1kb) | LRRC37A3 | -746 |
| chr17 | 73645275 | 73645498 | Promoter (1-2kb) | SDK2 | -1186 |
| chr17 | 74325693 | 74325961 | Promoter (<=1kb) | KIF19 | -251 |
| chr17 | 74431325 | 74431612 | Promoter (<=1kb) | GPRC5C | 0 |
| chr17 | 74529275 | 74529466 | Promoter (1-2kb) | CD300LB | 2000 |
| chr17 | 74971669 | 74972142 | Promoter (<=1kb) | HID1 | 592 |
| chr17 | 75225431 | 75225832 | Promoter (2-3kb) | NUP85 | -2329 |
| chr17 | 75475199 | 75475517 | Promoter (<=1kb) | TMEM94 | -981 |
| chr17 | 75475707 | 75476004 | Promoter (<=1kb) | TMEM94 | -494 |
| chr17 | 75506864 | 75507178 | Promoter (2-3kb) | CASKIN2 | 2710 |
| chr17 | 75509165 | 75509440 | Promoter (<=1kb) | CASKIN2 | 448 |
| chr17 | 75535255 | 75535448 | Promoter (2-3kb) | LLGL2 | 2762 |
| chr17 | 75589975 | 75590166 | Promoter (1-2kb) | MYO15B | 1917 |
| chr17 | 75724243 | 75724618 | Promoter (<=1kb) | ITGB4 | -78 |
| chr17 | 75842207 | 75842715 | Promoter (1-2kb) | UNC13D | 1635 |
| chr17 | 76074483 | 76074928 | Promoter (<=1kb) | GALR2 | 0 |
| chr17 | 76307528 | 76308064 | Promoter (<=1kb) | QRICH2 | 0 |
| chr17 | 76376617 | 76376933 | Promoter (<=1kb) | SPHK1 | 0 |
| chr17 | 76688056 | 76688301 | Promoter (<=1kb) | MXRA7 | 404 |
| chr17 | 77374819 | 77375003 | Promoter (1-2kb) | 9-Sep | -1080 |
| chr17 | 77476052 | 77476317 | Promoter (<=1kb) | 9-Sep | 809 |
| chr17 | 78121536 | 78121724 | Promoter (1-2kb) | TMC6 | 1024 |
| chr17 | 78146158 | 78146423 | Promoter (<=1kb) | C17orf99 | 0 |
| chr17 | 78232275 | 78232630 | Promoter (<=1kb) | TMEM235 | 251 |
| chr17 | 78855723 | 78856112 | Promoter (<=1kb) | AC022966.1 | 245 |
| chr17 | 79515226 | 79515418 | Promoter (<=1kb) | RBFOX3 | 730 |
| chr17 | 80313472 | 80313677 | Promoter (1-2kb) | AC124319.1 | -1974 |
| chr17 | 81129199 | 81129377 | Promoter (2-3kb) | AATK | 2571 |
| chr17 | 81527275 | 81527740 | Promoter (<=1kb) | FSCN2 | -656 |
| chr17 | 81823497 | 81823693 | Promoter (<=1kb) | MCRIP1 | 794 |
| chr17 | 82317560 | 82318140 | Promoter (<=1kb) | CD7 | 0 |
| chr18 | 2846974 | 2847937 | Promoter (<=1kb) | EMILIN2 | 0 |
| chr18 | 5196997 | 5197281 | Promoter (<=1kb) | AKAIN1 | 0 |
| chr18 | 5894663 | 5894860 | Promoter (1-2kb) | TMEM200C | 1095 |
| chr18 | 6414496 | 6415162 | Promoter (<=1kb) | L3MBTL4 | 0 |
| chr18 | 6967881 | 6968222 | Promoter (1-2kb) | LAMA1 | -1667 |
| chr18 | 9707867 | 9708445 | Promoter (<=1kb) | RAB31 | 0 |
| chr18 | 10453789 | 10454208 | Promoter (<=1kb) | APCDD1 | -420 |
| chr18 | 11146526 | 11146755 | Promoter (1-2kb) | PIEZO2 | 1833 |
| chr18 | 12254200 | 12254679 | Promoter (<=1kb) | CIDEA | 0 |
| chr18 | 21706438 | 21706771 | Promoter (1-2kb) | ABHD3 | -1633 |
| chr18 | 27184456 | 27184765 | Promoter (<=1kb) | CHST9 | 533 |
| chr18 | 37568264 | 37568450 | Promoter (2-3kb) | CELF4 | -2227 |
| chr18 | 46756574 | 46756777 | Promoter (<=1kb) | ST8SIA5 | 32 |
| chr18 | 47247879 | 47248462 | Promoter (<=1kb) | SKOR2 | 721 |
| chr18 | 49851778 | 49852056 | Promoter (1-2kb) | MYO5B | -1932 |
| chr18 | 54828575 | 54828764 | Promoter (<=1kb) | RAB27B | 376 |
| chr18 | 57803012 | 57803485 | Promoter (<=1kb) | ATP8B1 | 0 |
| chr18 | 69950378 | 69950602 | Promoter (2-3kb) | CD226 | -2525 |
| chr18 | 76495195 | 76495462 | Promoter (<=1kb) | ZNF516 | -5 |
| chr18 | 77252876 | 77253047 | Promoter (2-3kb) | GALR1 | 2327 |
| chr18 | 78993703 | 78993897 | Promoter (2-3kb) | SALL3 | 2021 |
| chr19 | 291742 | 291930 | Promoter (<=1kb) | PLPP2 | -238 |
| chr19 | 378289 | 378473 | Promoter (1-2kb) | THEG | -1619 |
| chr19 | 411938 | 412188 | Promoter (2-3kb) | C2CD4C | -2768 |
| chr19 | 640754 | 641125 | Promoter (<=1kb) | FGF22 | 859 |
| chr19 | 709452 | 710062 | Promoter (<=1kb) | PALM | 351 |
| chr19 | 821326 | 821915 | Promoter (<=1kb) | PLPPR3 | 37 |
| chr19 | 911769 | 911950 | Promoter (1-2kb) | R3HDM4 | 1207 |
| chr19 | 1584903 | 1585411 | Promoter (<=1kb) | MBD3 | 119 |
| chr19 | 1881864 | 1882296 | Promoter (<=1kb) | ABHD17A | -279 |
| chr19 | 1907786 | 1908136 | Promoter (2-3kb) | SCAMP4 | 2382 |
| chr19 | 2079566 | 2080217 | Promoter (<=1kb) | MOB3A | -847 |
| chr19 | 2721619 | 2722005 | Promoter (<=1kb) | DIRAS1 | -201 |
| chr19 | 2906623 | 2906985 | Promoter (<=1kb) | ZNF57 | -123 |
| chr19 | 3046433 | 3046860 | Promoter (<=1kb) | TLE2 | -645 |
| chr19 | 3314672 | 3314869 | Promoter (<=1kb) | NFIC | 269 |
| chr19 | 3710442 | 3710625 | Promoter (1-2kb) | TJP3 | 1939 |
| chr19 | 3771922 | 3772133 | Promoter (<=1kb) | RAX2 | 78 |
| chr19 | 3784962 | 3785256 | Promoter (1-2kb) | MATK | 1026 |
| chr19 | 3868584 | 3869190 | Promoter (<=1kb) | ZFR2 | 0 |
| chr19 | 4543044 | 4544295 | Promoter (2-3kb) | LRG1 | -2570 |
| chr19 | 4713354 | 4713853 | Promoter (<=1kb) | DPP9 | 453 |
| chr19 | 5013335 | 5013722 | Promoter (2-3kb) | KDM4B | -2603 |
| chr19 | 5018721 | 5019020 | Promoter (2-3kb) | KDM4B | 2396 |
| chr19 | 5567722 | 5567911 | Promoter (<=1kb) | TINCR | 42 |
| chr19 | 5841340 | 5841560 | Promoter (1-2kb) | FUT6 | -1609 |
| chr19 | 6380858 | 6381528 | Promoter (1-2kb) | PSPN | -1800 |
| chr19 | 6465539 | 6465933 | Promoter (1-2kb) | CRB3 | 1008 |
| chr19 | 7350309 | 7350512 | Promoter (1-2kb) | AC119396.1 | 1366 |
| chr19 | 7745204 | 7745511 | Promoter (1-2kb) | CD209 | 2000 |
| chr19 | 7843136 | 7843778 | Promoter (2-3kb) | EVI5L | -2765 |
| chr19 | 8148285 | 8148715 | Promoter (<=1kb) | FBN3 | 154 |
| chr19 | 8148997 | 8149262 | Promoter (<=1kb) | FBN3 | -128 |
| chr19 | 8824046 | 8824254 | Promoter (1-2kb) | ZNF558 | -1157 |
| chr19 | 10010145 | 10010338 | Promoter (<=1kb) | COL5A3 | 133 |
| chr19 | 10378308 | 10378610 | Promoter (<=1kb) | TYK2 | 0 |
| chr19 | 10430696 | 10430901 | Promoter (<=1kb) | PDE4A | 0 |
| chr19 | 10625454 | 10625867 | Promoter (<=1kb) | SLC44A2 | 0 |
| chr19 | 10927500 | 10928083 | Promoter (<=1kb) | YIPF2 | 502 |
| chr19 | 11196767 | 11196971 | Promoter (<=1kb) | KANK2 | 550 |
| chr19 | 12052092 | 12052287 | Promoter (<=1kb) | ZNF878 | 652 |
| chr19 | 13025040 | 13025282 | Promoter (<=1kb) | NFIX | 101 |
| chr19 | 13504124 | 13504361 | Promoter (1-2kb) | CACNA1A | 1863 |
| chr19 | 13763162 | 13763444 | Promoter (1-2kb) | MRI1 | -1088 |
| chr19 | 14582968 | 14583148 | Promoter (<=1kb) | CLEC17A | 0 |
| chr19 | 15010405 | 15011130 | Promoter (<=1kb) | CCDC105 | 0 |
| chr19 | 15508041 | 15508479 | Promoter (<=1kb) | CYP4F22 | -14 |
| chr19 | 17824510 | 17824940 | Promoter (2-3kb) | INSL3 | -2936 |
| chr19 | 17973743 | 17973987 | Promoter (<=1kb) | KCNN1 | 0 |
| chr19 | 18397996 | 18398389 | Promoter (<=1kb) | LRRC25 | -379 |
| chr19 | 18476431 | 18476628 | Promoter (<=1kb) | ELL | -498 |
| chr19 | 18735929 | 18736104 | Promoter (<=1kb) | CRTC1 | 320 |
| chr19 | 18788515 | 18788818 | Promoter (2-3kb) | COMP | 2487 |
| chr19 | 19527098 | 19527328 | Promoter (1-2kb) | YJEFN3 | -1533 |
| chr19 | 29664049 | 29664508 | Promoter (<=1kb) | PLEKHF1 | -548 |
| chr19 | 33134720 | 33134940 | Promoter (2-3kb) | WDR88 | 2626 |
| chr19 | 33195420 | 33195704 | Promoter (<=1kb) | LRP3 | 836 |
| chr19 | 35115518 | 35115760 | Promoter (<=1kb) | FXYD3 | -119 |
| chr19 | 35156777 | 35156990 | Promoter (1-2kb) | FXYD5 | 1835 |
| chr19 | 35755743 | 35756047 | Promoter (<=1kb) | HSPB6 | 962 |
| chr19 | 36370358 | 36370649 | Promoter (2-3kb) | ZFP14 | -2385 |
| chr19 | 38256094 | 38256317 | Promoter (<=1kb) | PPP1R14A | 55 |
| chr19 | 38309139 | 38309334 | Promoter (<=1kb) | YIF1B | 309 |
| chr19 | 38815292 | 38815462 | Promoter (1-2kb) | LGALS4 | -1928 |
| chr19 | 38879340 | 38879605 | Promoter (1-2kb) | RINL | -1061 |
| chr19 | 39515274 | 39515539 | Promoter (<=1kb) | SELENOV | 161 |
| chr19 | 39515989 | 39516308 | Promoter (<=1kb) | SELENOV | 876 |
| chr19 | 40466669 | 40466957 | Promoter (<=1kb) | SPTBN4 | -262 |
| chr19 | 40991246 | 40991440 | Promoter (<=1kb) | CYP2B6 | 0 |
| chr19 | 41085946 | 41086137 | Promoter (2-3kb) | CYP2A13 | -2335 |
| chr19 | 41878915 | 41879134 | Promoter (1-2kb) | CD79A | 1610 |
| chr19 | 41990969 | 41991212 | Promoter (2-3kb) | ATP1A3 | 2255 |
| chr19 | 41992160 | 41992375 | Promoter (1-2kb) | ATP1A3 | 1092 |
| chr19 | 42131126 | 42131487 | Promoter (<=1kb) | POU2F2 | 932 |
| chr19 | 42593210 | 42593403 | Promoter (1-2kb) | CEACAM8 | 1527 |
| chr19 | 42741504 | 42741813 | Promoter (<=1kb) | PSG3 | -935 |
| chr19 | 42766801 | 42766979 | Promoter (1-2kb) | PSG8 | -1122 |
| chr19 | 42933946 | 42934194 | Promoter (2-3kb) | PSG7 | 2904 |
| chr19 | 42935312 | 42935480 | Promoter (1-2kb) | PSG7 | 1618 |
| chr19 | 43639905 | 43640227 | Promoter (<=1kb) | CADM4 | -66 |
| chr19 | 43782457 | 43783116 | Promoter (1-2kb) | KCNN4 | -1196 |
| chr19 | 43820283 | 43820570 | Promoter (<=1kb) | LYPD5 | 64 |
| chr19 | 44669615 | 44669841 | Promoter (1-2kb) | CEACAM19 | -1611 |
| chr19 | 44750960 | 44751132 | Promoter (2-3kb) | BCL3 | 2413 |
| chr19 | 44777825 | 44778056 | Promoter (<=1kb) | CBLC | 0 |
| chr19 | 45075972 | 45076294 | Promoter (<=1kb) | ZNF296 | 293 |
| chr19 | 45601482 | 45602354 | Promoter (<=1kb) | GPR4 | 0 |
| chr19 | 46000617 | 46001000 | Promoter (2-3kb) | CCDC61 | -2006 |
| chr19 | 46022484 | 46022919 | Promoter (<=1kb) | PGLYRP1 | 146 |
| chr19 | 46427844 | 46428183 | Promoter (<=1kb) | PNMA8C | 768 |
| chr19 | 47680235 | 47680694 | Promoter (1-2kb) | BICRA | 1273 |
| chr19 | 47804897 | 47805268 | Promoter (1-2kb) | TPRX1 | -1208 |
| chr19 | 48443761 | 48444087 | Promoter (1-2kb) | GRWD1 | -1686 |
| chr19 | 48453073 | 48453246 | Promoter (2-3kb) | KCNJ14 | -2263 |
| chr19 | 48508930 | 48509208 | Promoter (2-3kb) | LMTK3 | 2596 |
| chr19 | 48752827 | 48753170 | Promoter (<=1kb) | FUT1 | 490 |
| chr19 | 49006909 | 49007084 | Promoter (2-3kb) | RUVBL2 | 2620 |
| chr19 | 49057892 | 49058109 | Promoter (<=1kb) | CGB7 | -141 |
| chr19 | 49324915 | 49325320 | Promoter (<=1kb) | SLC6A16 | 0 |
| chr19 | 49423314 | 49423484 | Promoter (<=1kb) | PTH2 | 0 |
| chr19 | 49463994 | 49464530 | Promoter (1-2kb) | AC010619.1 | 1242 |
| chr19 | 49464874 | 49465168 | Promoter (2-3kb) | AC010619.1 | 2122 |
| chr19 | 49595700 | 49596205 | Promoter (<=1kb) | PRR12 | 178 |
| chr19 | 49745152 | 49745493 | Promoter (1-2kb) | TSKS | 1885 |
| chr19 | 50517530 | 50517724 | Promoter (2-3kb) | ASPDH | -2840 |
| chr19 | 50687427 | 50687636 | Promoter (1-2kb) | SHANK1 | 1768 |
| chr19 | 51019184 | 51019380 | Promoter (<=1kb) | KLK10 | 384 |
| chr19 | 51417294 | 51417473 | Promoter (<=1kb) | SIGLEC10 | 108 |
| chr19 | 51531589 | 51531837 | Promoter (<=1kb) | SIGLEC6 | 0 |
| chr19 | 51726395 | 51726569 | Promoter (2-3kb) | HAS1 | -2401 |
| chr19 | 52725672 | 52726130 | Promoter (2-3kb) | ZNF611 | 2656 |
| chr19 | 52811767 | 52811936 | Promoter (1-2kb) | ZNF28 | -1320 |
| chr19 | 54074172 | 54074659 | Promoter (1-2kb) | TARM1 | 1886 |
| chr19 | 54173167 | 54173467 | Promoter (<=1kb) | TMC4 | 0 |
| chr19 | 54222623 | 54223059 | Promoter (<=1kb) | LILRB3 | 0 |
| chr19 | 54256624 | 54257097 | Promoter (<=1kb) | LILRB5 | 165 |
| chr19 | 54279751 | 54280023 | Promoter (<=1kb) | LILRB2 | 475 |
| chr19 | 55304944 | 55305313 | Promoter (2-3kb) | BRSK1 | 2710 |
| chr19 | 55535970 | 55536343 | Promoter (<=1kb) | SBK2 | 0 |
| chr19 | 55578241 | 55579131 | Promoter (1-2kb) | ZNF579 | 1690 |
| chr19 | 55614627 | 55615015 | Promoter (<=1kb) | ZNF865 | -957 |
| chr19 | 55615509 | 55615984 | Promoter (<=1kb) | ZNF865 | 0 |
| chr19 | 55661059 | 55661437 | Promoter (1-2kb) | U2AF2 | -1062 |
| chr19 | 57231231 | 57231547 | Promoter (<=1kb) | AURKC | 0 |
| chr19 | 57241427 | 57241621 | Promoter (<=1kb) | ZNF805 | 535 |
| chr19 | 57584171 | 57584545 | Promoter (<=1kb) | ZIK1 | 0 |
| chr19 | 58229828 | 58230018 | Promoter (<=1kb) | ZNF544 | 360 |
| chr19 | 58246647 | 58246828 | Promoter (2-3kb) | ZNF544 | 2684 |
| chr2 | 46246 | 46760 | Promoter (<=1kb) | FAM110C | 0 |
| chr2 | 6865388 | 6865588 | Promoter (<=1kb) | CMPK2 | 108 |
| chr2 | 20665367 | 20665669 | Promoter (<=1kb) | GDF7 | -995 |
| chr2 | 20824060 | 20824270 | Promoter (<=1kb) | LDAH | -930 |
| chr2 | 24010029 | 24011119 | Promoter (<=1kb) | MFSD2B | 0 |
| chr2 | 25114041 | 25114244 | Promoter (1-2kb) | EFR3B | -1744 |
| chr2 | 25251549 | 25252028 | Promoter (<=1kb) | DNMT3A | 283 |
| chr2 | 25341040 | 25341273 | Promoter (<=1kb) | DNMT3A | 612 |
| chr2 | 26172913 | 26173090 | Promoter (<=1kb) | GAREM2 | -1 |
| chr2 | 26474421 | 26474953 | Promoter (2-3kb) | OTOF | 2940 |
| chr2 | 26480190 | 26480463 | Promoter (2-3kb) | OTOF | -2148 |
| chr2 | 26562123 | 26562292 | Promoter (<=1kb) | C2orf70 | -290 |
| chr2 | 26692687 | 26693655 | Promoter (<=1kb) | KCNK3 | 0 |
| chr2 | 27149720 | 27150274 | Promoter (<=1kb) | TCF23 | 716 |
| chr2 | 27342805 | 27342990 | Promoter (<=1kb) | GTF3C2 | 642 |
| chr2 | 27539759 | 27540740 | Promoter (2-3kb) | C2orf16 | 2373 |
| chr2 | 46298924 | 46299094 | Promoter (1-2kb) | EPAS1 | 1522 |
| chr2 | 50346508 | 50346952 | Promoter (<=1kb) | NRXN1 | 302 |
| chr2 | 50658972 | 50659153 | Promoter (2-3kb) | NRXN1 | -2562 |
| chr2 | 68643057 | 68643347 | Promoter (<=1kb) | PROKR1 | -242 |
| chr2 | 70767257 | 70767467 | Promoter (<=1kb) | ADD2 | 248 |
| chr2 | 72916415 | 72917141 | Promoter (<=1kb) | EMX1 | 155 |
| chr2 | 73284023 | 73284289 | Promoter (<=1kb) | FBXO41 | 21 |
| chr2 | 73284480 | 73285136 | Promoter (<=1kb) | FBXO41 | -49 |
| chr2 | 74330231 | 74330665 | Promoter (2-3kb) | SLC4A5 | -2065 |
| chr2 | 74415345 | 74415583 | Promoter (<=1kb) | C2orf81 | 1000 |
| chr2 | 74514183 | 74515374 | Promoter (<=1kb) | TLX2 | 0 |
| chr2 | 85888101 | 85888278 | Promoter (<=1kb) | ST3GAL5 | 123 |
| chr2 | 95275933 | 95276156 | Promoter (1-2kb) | PROM2 | 1480 |
| chr2 | 96860082 | 96860305 | Promoter (1-2kb) | ANKRD39 | -1987 |
| chr2 | 96895601 | 96895928 | Promoter (2-3kb) | FAM178B | 2205 |
| chr2 | 97734300 | 97734690 | Promoter (<=1kb) | ZAP70 | 0 |
| chr2 | 98518357 | 98518622 | Promoter (1-2kb) | INPP4A | -1427 |
| chr2 | 105328773 | 105329078 | Promoter (<=1kb) | TGFBRAP1 | 956 |
| chr2 | 110115423 | 110115649 | Promoter (<=1kb) | MALL | 373 |
| chr2 | 110116373 | 110116659 | Promoter (<=1kb) | MALL | 0 |
| chr2 | 110732487 | 110732783 | Promoter (<=1kb) | ACOXL | 0 |
| chr2 | 112720881 | 112721061 | Promoter (<=1kb) | NT5DC4 | -425 |
| chr2 | 118847857 | 118848233 | Promoter (<=1kb) | EN1 | -179 |
| chr2 | 118941744 | 118941927 | Promoter (<=1kb) | MARCO | -239 |
| chr2 | 127416558 | 127416818 | Promoter (1-2kb) | PROC | -1609 |
| chr2 | 127420022 | 127420384 | Promoter (<=1kb) | PROC | 140 |
| chr2 | 149038514 | 149039039 | Promoter (<=1kb) | LYPD6B | 0 |
| chr2 | 150485406 | 150486329 | Promoter (<=1kb) | RND3 | -24 |
| chr2 | 161416844 | 161417320 | Promoter (<=1kb) | TBR1 | -575 |
| chr2 | 169575144 | 169575450 | Promoter (1-2kb) | FASTKD1 | -1271 |
| chr2 | 170716782 | 170717109 | Promoter (1-2kb) | SP5 | 1431 |
| chr2 | 172735814 | 172736084 | Promoter (<=1kb) | RAPGEF4 | 0 |
| chr2 | 176100037 | 176100325 | Promoter (<=1kb) | HOXD12 | 235 |
| chr2 | 216081584 | 216082023 | Promoter (<=1kb) | PECR | 0 |
| chr2 | 217905448 | 217905695 | Promoter (1-2kb) | TNS1 | -1635 |
| chr2 | 218738215 | 218738478 | Promoter (<=1kb) | TTLL4 | 641 |
| chr2 | 218834808 | 218835045 | Promoter (2-3kb) | PRKAG3 | -2722 |
| chr2 | 218858177 | 218858420 | Promoter (1-2kb) | WNT6 | -1401 |
| chr2 | 218880290 | 218880496 | Promoter (<=1kb) | WNT10A | 0 |
| chr2 | 219308315 | 219308629 | Promoter (<=1kb) | PTPRN | 355 |
| chr2 | 219436080 | 219436395 | Promoter (1-2kb) | SPEG | 1234 |
| chr2 | 219629523 | 219629742 | Promoter (1-2kb) | SLC4A3 | 1862 |
| chr2 | 231592907 | 231593102 | Promoter (<=1kb) | TEX44 | 6 |
| chr2 | 232406145 | 232406388 | Promoter (<=1kb) | ALPG | -455 |
| chr2 | 233060566 | 233060750 | Promoter (<=1kb) | INPP5D | 168 |
| chr2 | 235707409 | 235707894 | Promoter (1-2kb) | AGAP1 | -1283 |
| chr2 | 237627105 | 237627468 | Promoter (<=1kb) | LRRFIP1 | -108 |
| chr2 | 238097417 | 238097944 | Promoter (2-3kb) | ESPNL | -2213 |
| chr2 | 240030793 | 240031023 | Promoter (<=1kb) | OR6B2 | -337 |
| chr2 | 240433282 | 240433554 | Promoter (2-3kb) | GPC1 | -2117 |
| chr2 | 240870527 | 240870773 | Promoter (2-3kb) | AGXT | 2048 |
| chr2 | 241619865 | 241620212 | Promoter (2-3kb) | THAP4 | -2364 |
| chr2 | 241858539 | 241858927 | Promoter (<=1kb) | PDCD1 | 0 |
| chr20 | 774023 | 774347 | Promoter (1-2kb) | SLC52A3 | 1668 |
| chr20 | 2525275 | 2525454 | Promoter (<=1kb) | ZNF343 | -753 |
| chr20 | 2536685 | 2536914 | Promoter (<=1kb) | TMC2 | 78 |
| chr20 | 2798260 | 2798638 | Promoter (1-2kb) | CPXM1 | 1999 |
| chr20 | 2871884 | 2872062 | Promoter (1-2kb) | PTPRA | -1427 |
| chr20 | 3166888 | 3167122 | Promoter (1-2kb) | LZTS3 | 1439 |
| chr20 | 3730020 | 3730193 | Promoter (2-3kb) | HSPA12B | -2474 |
| chr20 | 4248628 | 4248839 | Promoter (<=1kb) | ADRA1D | 235 |
| chr20 | 5316827 | 5317310 | Promoter (2-3kb) | PROKR2 | -2458 |
| chr20 | 9838620 | 9839100 | Promoter (<=1kb) | PAK5 | 0 |
| chr20 | 13220424 | 13220901 | Promoter (<=1kb) | ISM1 | -870 |
| chr20 | 23826029 | 23826198 | Promoter (<=1kb) | CST2 | 533 |
| chr20 | 31470281 | 31470975 | Promoter (2-3kb) | DEFB124 | 2038 |
| chr20 | 31471947 | 31472121 | Promoter (<=1kb) | DEFB124 | 892 |
| chr20 | 31870717 | 31870960 | Promoter (<=1kb) | DUSP15 | 0 |
| chr20 | 32649540 | 32649737 | Promoter (2-3kb) | C20orf203 | 2022 |
| chr20 | 34559676 | 34560515 | Promoter (<=1kb) | MAP1LC3A | 958 |
| chr20 | 35433927 | 35434310 | Promoter (<=1kb) | GDF5OS | 898 |
| chr20 | 35437214 | 35437389 | Promoter (<=1kb) | GDF5 | 854 |
| chr20 | 35438864 | 35439145 | Promoter (<=1kb) | GDF5 | -621 |
| chr20 | 36305789 | 36306162 | Promoter (<=1kb) | DLGAP4 | -174 |
| chr20 | 36307761 | 36308041 | Promoter (1-2kb) | DLGAP4 | 1380 |
| chr20 | 36541229 | 36542510 | Promoter (<=1kb) | MYL9 | 0 |
| chr20 | 36878946 | 36879313 | Promoter (2-3kb) | TLDC2 | 2815 |
| chr20 | 43538014 | 43538194 | Promoter (1-2kb) | Z98752.3 | -1977 |
| chr20 | 43540068 | 43540274 | Promoter (<=1kb) | Z98752.3 | 0 |
| chr20 | 44186457 | 44186830 | Promoter (<=1kb) | JPH2 | 263 |
| chr20 | 45469672 | 45470115 | Promoter (<=1kb) | WFDC2 | 0 |
| chr20 | 46308765 | 46308938 | Promoter (<=1kb) | CDH22 | -267 |
| chr20 | 47785064 | 47785237 | Promoter (1-2kb) | SULF2 | 1317 |
| chr20 | 48920130 | 48920372 | Promoter (1-2kb) | ARFGEF2 | -1518 |
| chr20 | 57265234 | 57265406 | Promoter (<=1kb) | BMP7 | 936 |
| chr20 | 57524396 | 57524904 | Promoter (<=1kb) | CTCFL | 0 |
| chr20 | 57616731 | 57617421 | Promoter (2-3kb) | ZBP1 | 2973 |
| chr20 | 59603612 | 59603937 | Promoter (<=1kb) | PHACTR3 | -611 |
| chr20 | 62159145 | 62159414 | Promoter (2-3kb) | SS18L1 | -2004 |
| chr20 | 62657298 | 62657544 | Promoter (<=1kb) | SLCO4A1 | 939 |
| chr20 | 63257034 | 63257232 | Promoter (2-3kb) | NKAIN4 | -2494 |
| chr20 | 63290285 | 63290505 | Promoter (2-3kb) | COL20A1 | -2681 |
| chr20 | 63472429 | 63472629 | Promoter (<=1kb) | KCNQ2 | 0 |
| chr20 | 63567237 | 63567504 | Promoter (<=1kb) | HELZ2 | 570 |
| chr20 | 63977411 | 63977740 | Promoter (1-2kb) | SAMD10 | 1902 |
| chr20 | 64052187 | 64052373 | Promoter (2-3kb) | SOX18 | -2546 |
| chr20 | 64153453 | 64153646 | Promoter (1-2kb) | MYT1 | 1662 |
| chr21 | 10520994 | 10521422 | Promoter (<=1kb) | TPTE | -131 |
| chr21 | 26141987 | 26142244 | Promoter (1-2kb) | APP | -1527 |
| chr21 | 30486420 | 30486652 | Promoter (<=1kb) | KRTAP19-2 | 784 |
| chr21 | 32412413 | 32412976 | Promoter (<=1kb) | EVA1C | 0 |
| chr21 | 32586876 | 32587141 | Promoter (1-2kb) | TCP10L | -1343 |
| chr21 | 34670331 | 34670561 | Promoter (<=1kb) | CLIC6 | 942 |
| chr21 | 36479417 | 36479620 | Promoter (<=1kb) | CLDN14 | 470 |
| chr21 | 36539648 | 36540061 | Promoter (2-3kb) | CLDN14 | 2539 |
| chr21 | 36699244 | 36699487 | Promoter (<=1kb) | SIM2 | 110 |
| chr21 | 37006700 | 37006930 | Promoter (<=1kb) | RIPPLY3 | 137 |
| chr21 | 41360364 | 41360663 | Promoter (1-2kb) | MX2 | -1280 |
| chr21 | 41507108 | 41507622 | Promoter (<=1kb) | TMPRSS2 | 360 |
| chr21 | 41508127 | 41508411 | Promoter (<=1kb) | TMPRSS2 | 0 |
| chr21 | 42312452 | 42312723 | Promoter (2-3kb) | TFF3 | 2630 |
| chr21 | 42513653 | 42513889 | Promoter (<=1kb) | SLC37A1 | -25 |
| chr21 | 43786630 | 43786816 | Promoter (2-3kb) | RRP1 | -2697 |
| chr21 | 43866790 | 43866999 | Promoter (1-2kb) | AGPAT3 | 1604 |
| chr21 | 43952782 | 43952990 | Promoter (1-2kb) | AGPAT3 | -1558 |
| chr21 | 44579097 | 44579333 | Promoter (1-2kb) | KRTAP10-5 | 1271 |
| chr21 | 44913156 | 44913397 | Promoter (1-2kb) | ITGB2 | -1696 |
| chr21 | 44919250 | 44919434 | Promoter (1-2kb) | ITGB2 | 1421 |
| chr21 | 45543219 | 45543419 | Promoter (<=1kb) | SLC19A1 | -748 |
| chr21 | 46111114 | 46111293 | Promoter (<=1kb) | COL6A2 | -121 |
| chr21 | 46153778 | 46154284 | Promoter (1-2kb) | FTCD | 1283 |
| chr21 | 46318272 | 46318463 | Promoter (<=1kb) | C21orf58 | 674 |
| chr21 | 46606325 | 46606646 | Promoter (1-2kb) | S100B | -1117 |
| chr22 | 19524077 | 19524302 | Promoter (<=1kb) | CLDN5 | 98 |
| chr22 | 19758528 | 19758759 | Promoter (1-2kb) | TBX1 | 1825 |
| chr22 | 19933950 | 19934193 | Promoter (<=1kb) | TXNRD2 | -424 |
| chr22 | 20150723 | 20151086 | Promoter (<=1kb) | CCDC188 | 0 |
| chr22 | 20267996 | 20268484 | Promoter (<=1kb) | RTN4R | 47 |
| chr22 | 20392499 | 20392748 | Promoter (1-2kb) | ZNF74 | -1367 |
| chr22 | 20440404 | 20440679 | Promoter (2-3kb) | SCARF2 | -2578 |
| chr22 | 20965461 | 20966289 | Promoter (<=1kb) | AIFM3 | 298 |
| chr22 | 20967030 | 20967293 | Promoter (<=1kb) | AIFM3 | 0 |
| chr22 | 21941334 | 21941737 | Promoter (2-3kb) | PPM1F | -2750 |
| chr22 | 23753090 | 23753380 | Promoter (1-2kb) | VPREB3 | 1029 |
| chr22 | 23763756 | 23764061 | Promoter (<=1kb) | C22orf15 | 533 |
| chr22 | 23765990 | 23766213 | Promoter (1-2kb) | CHCHD10 | 1663 |
| chr22 | 23767311 | 23767523 | Promoter (<=1kb) | CHCHD10 | 353 |
| chr22 | 23772691 | 23773401 | Promoter (<=1kb) | MMP11 | 0 |
| chr22 | 24433361 | 24433940 | Promoter (1-2kb) | ADORA2A | 1242 |
| chr22 | 24495327 | 24495607 | Promoter (<=1kb) | UPB1 | 85 |
| chr22 | 24585991 | 24586325 | Promoter (2-3kb) | GGT1 | 2241 |
| chr22 | 24594132 | 24594532 | Promoter (1-2kb) | LRRC75B | -1069 |
| chr22 | 24628318 | 24628668 | Promoter (<=1kb) | GGT1 | 874 |
| chr22 | 24942499 | 24942823 | Promoter (2-3kb) | TMEM211 | -2964 |
| chr22 | 25028193 | 25028667 | Promoter (<=1kb) | KIAA1671 | 219 |
| chr22 | 25029469 | 25029744 | Promoter (1-2kb) | KIAA1671 | 1495 |
| chr22 | 29061218 | 29061417 | Promoter (<=1kb) | C22orf31 | 427 |
| chr22 | 30694978 | 30695153 | Promoter (<=1kb) | OSBP2 | 89 |
| chr22 | 30822130 | 30822487 | Promoter (<=1kb) | OSBP2 | -29 |
| chr22 | 31126670 | 31127199 | Promoter (<=1kb) | INPP5J | -561 |
| chr22 | 31141237 | 31141543 | Promoter (<=1kb) | PLA2G3 | -630 |
| chr22 | 31629415 | 31629690 | Promoter (1-2kb) | PISD | 1134 |
| chr22 | 36253170 | 36253353 | Promoter (<=1kb) | APOL1 | 29 |
| chr22 | 36367612 | 36367803 | Promoter (2-3kb) | MYH9 | -2500 |
| chr22 | 36818723 | 36819098 | Promoter (<=1kb) | PVALB | 381 |
| chr22 | 37022443 | 37022927 | Promoter (<=1kb) | MPST | -110 |
| chr22 | 37147283 | 37147548 | Promoter (2-3kb) | IL2RB | 2442 |
| chr22 | 37241287 | 37241504 | Promoter (2-3kb) | RAC2 | 2744 |
| chr22 | 37824468 | 37824884 | Promoter (1-2kb) | GALR3 | 1086 |
| chr22 | 38082558 | 38082823 | Promoter (<=1kb) | SLC16A8 | 319 |
| chr22 | 38112245 | 38112599 | Promoter (1-2kb) | BAIAP2L2 | -1575 |
| chr22 | 38955482 | 38955695 | Promoter (1-2kb) | APOBEC3A | -1827 |
| chr22 | 38956046 | 38956394 | Promoter (1-2kb) | APOBEC3A | -1128 |
| chr22 | 39102173 | 39102391 | Promoter (1-2kb) | APOBEC3H | 1894 |
| chr22 | 39947586 | 39947927 | Promoter (<=1kb) | GRAP2 | 769 |
| chr22 | 39994680 | 39995120 | Promoter (<=1kb) | FAM83F | 0 |
| chr22 | 40553746 | 40553916 | Promoter (1-2kb) | MRTFA | -1435 |
| chr22 | 41951998 | 41952353 | Promoter (<=1kb) | LINC00634 | 0 |
| chr22 | 41970162 | 41970403 | Promoter (<=1kb) | 3-Sep | 687 |
| chr22 | 42017172 | 42017375 | Promoter (2-3kb) | WBP2NL | -2338 |
| chr22 | 42129173 | 42129355 | Promoter (1-2kb) | CYP2D6 | 1450 |
| chr22 | 42214807 | 42215001 | Promoter (<=1kb) | TCF20 | 439 |
| chr22 | 42434360 | 42434591 | Promoter (1-2kb) | NFAM1 | -1965 |
| chr22 | 42550575 | 42551021 | Promoter (2-3kb) | SERHL2 | -2841 |
| chr22 | 42637594 | 42638051 | Promoter (<=1kb) | CYB5R3 | -704 |
| chr22 | 42720307 | 42720896 | Promoter (<=1kb) | A4GALT | 0 |
| chr22 | 42962638 | 42963122 | Promoter (2-3kb) | PACSIN2 | -2757 |
| chr22 | 43111160 | 43111413 | Promoter (<=1kb) | BIK | 412 |
| chr22 | 43891953 | 43892142 | Promoter (<=1kb) | PNPLA5 | 0 |
| chr22 | 44676905 | 44677398 | Promoter (<=1kb) | PRR5 | 0 |
| chr22 | 45209953 | 45210208 | Promoter (2-3kb) | KIAA0930 | 2209 |
| chr22 | 46626231 | 46626466 | Promoter (<=1kb) | GRAMD4 | -295 |
| chr22 | 46626838 | 46627012 | Promoter (<=1kb) | GRAMD4 | 77 |
| chr22 | 50010754 | 50010958 | Promoter (1-2kb) | IL17REL | 1701 |
| chr3 | 8769329 | 8769585 | Promoter (<=1kb) | OXTR | 32 |
| chr3 | 12758766 | 12758971 | Promoter (<=1kb) | TMEM40 | 288 |
| chr3 | 13280976 | 13281177 | Promoter (2-3kb) | IQSEC1 | 2104 |
| chr3 | 13879466 | 13879746 | Promoter (<=1kb) | WNT7A | 375 |
| chr3 | 14672166 | 14672338 | Promoter (2-3kb) | C3orf20 | -2761 |
| chr3 | 25455849 | 25456059 | Promoter (2-3kb) | RARB | -2251 |
| chr3 | 36380437 | 36380907 | Promoter (<=1kb) | STAC | 0 |
| chr3 | 36944212 | 36944521 | Promoter (<=1kb) | TRANK1 | 536 |
| chr3 | 38308551 | 38308757 | Promoter (2-3kb) | SLC22A14 | 2615 |
| chr3 | 42685919 | 42686113 | Promoter (<=1kb) | KLHL40 | 400 |
| chr3 | 45147055 | 45147397 | Promoter (<=1kb) | CDCP1 | -633 |
| chr3 | 46463397 | 46463684 | Promoter (<=1kb) | LTF | 0 |
| chr3 | 46878771 | 46879039 | Promoter (1-2kb) | PTH1R | 1025 |
| chr3 | 48425627 | 48426140 | Promoter (1-2kb) | PLXNB1 | -1016 |
| chr3 | 48500785 | 48501693 | Promoter (2-3kb) | SHISA5 | 2357 |
| chr3 | 48621113 | 48621791 | Promoter (<=1kb) | TMEM89 | 64 |
| chr3 | 49013139 | 49013352 | Promoter (1-2kb) | WDR6 | 1505 |
| chr3 | 49199085 | 49199842 | Promoter (<=1kb) | CCDC36 | 0 |
| chr3 | 49722613 | 49722905 | Promoter (1-2kb) | GMPPB | 1044 |
| chr3 | 49803863 | 49804073 | Promoter (<=1kb) | INKA1 | 609 |
| chr3 | 50311559 | 50311996 | Promoter (<=1kb) | HYAL1 | 385 |
| chr3 | 51966187 | 51966368 | Promoter (1-2kb) | PCBP4 | 1077 |
| chr3 | 52795328 | 52795807 | Promoter (<=1kb) | ITIH3 | 530 |
| chr3 | 54121786 | 54122081 | Promoter (<=1kb) | CACNA2D3 | -466 |
| chr3 | 58009108 | 58009411 | Promoter (<=1kb) | FLNB | 708 |
| chr3 | 58588070 | 58588238 | Promoter (<=1kb) | FAM107A | -957 |
| chr3 | 69936155 | 69936429 | Promoter (<=1kb) | MITF | -158 |
| chr3 | 71582570 | 71582754 | Promoter (<=1kb) | FOXP1 | -622 |
| chr3 | 84959476 | 84959645 | Promoter (<=1kb) | CADM2 | 0 |
| chr3 | 97822390 | 97822630 | Promoter (<=1kb) | CRYBG3 | 350 |
| chr3 | 101677939 | 101678170 | Promoter (<=1kb) | ZBTB11 | -444 |
| chr3 | 119810237 | 119810438 | Promoter (2-3kb) | NR1I2 | 2986 |
| chr3 | 126525144 | 126525315 | Promoter (<=1kb) | CHST13 | 811 |
| chr3 | 126557520 | 126557842 | Promoter (1-2kb) | C3orf22 | 1123 |
| chr3 | 127580101 | 127580425 | Promoter (<=1kb) | TPRA1 | 12 |
| chr3 | 127671273 | 127671476 | Promoter (1-2kb) | ABTB1 | -1464 |
| chr3 | 127822821 | 127823063 | Promoter (<=1kb) | MGLL | 0 |
| chr3 | 127823434 | 127823925 | Promoter (<=1kb) | MGLL | -184 |
| chr3 | 127915194 | 127915605 | Promoter (<=1kb) | KBTBD12 | 0 |
| chr3 | 129530808 | 129531183 | Promoter (2-3kb) | RHO | 2168 |
| chr3 | 133399891 | 133400283 | Promoter (<=1kb) | BFSP2 | 0 |
| chr3 | 139019932 | 139020130 | Promoter (<=1kb) | PRR23B | 796 |
| chr3 | 142001206 | 142001445 | Promoter (<=1kb) | TFDP2 | -853 |
| chr3 | 142724763 | 142724944 | Promoter (<=1kb) | TRPC1 | 328 |
| chr3 | 142962663 | 142962878 | Promoter (<=1kb) | PAQR9 | 804 |
| chr3 | 157499442 | 157499887 | Promoter (<=1kb) | VEPH1 | 0 |
| chr3 | 184563699 | 184563907 | Promoter (1-2kb) | EPHB3 | 1915 |
| chr3 | 186717700 | 186717968 | Promoter (<=1kb) | KNG1 | 341 |
| chr3 | 195544651 | 195544820 | Promoter (1-2kb) | PPP1R2 | -1265 |
| chr4 | 304956 | 305326 | Promoter (<=1kb) | ZNF732 | 0 |
| chr4 | 1239331 | 1239630 | Promoter (1-2kb) | CTBP1 | -1809 |
| chr4 | 1309717 | 1309947 | Promoter (<=1kb) | MAEA | 0 |
| chr4 | 1368879 | 1369114 | Promoter (2-3kb) | UVSSA | 2802 |
| chr4 | 1376624 | 1376871 | Promoter (1-2kb) | UVSSA | 1717 |
| chr4 | 1952398 | 1952814 | Promoter (<=1kb) | NSD2 | 293 |
| chr4 | 2058475 | 2058853 | Promoter (<=1kb) | NAT8L | -659 |
| chr4 | 2286032 | 2286422 | Promoter (1-2kb) | ZFYVE28 | 1293 |
| chr4 | 2290425 | 2290745 | Promoter (2-3kb) | ZFYVE28 | -2710 |
| chr4 | 2425413 | 2425877 | Promoter (<=1kb) | CFAP99 | -599 |
| chr4 | 2755258 | 2755618 | Promoter (<=1kb) | TNIP2 | 713 |
| chr4 | 3317397 | 3317576 | Promoter (1-2kb) | RGS12 | 1329 |
| chr4 | 3486217 | 3486854 | Promoter (1-2kb) | DOK7 | 1673 |
| chr4 | 6563282 | 6563604 | Promoter (<=1kb) | PPP2R2C | 0 |
| chr4 | 7193478 | 7193765 | Promoter (<=1kb) | SORCS2 | 940 |
| chr4 | 37889965 | 37890575 | Promoter (<=1kb) | TBC1D1 | -512 |
| chr4 | 38807542 | 38808117 | Promoter (2-3kb) | TLR1 | -2751 |
| chr4 | 38867647 | 38868300 | Promoter (<=1kb) | FAM114A1 | 0 |
| chr4 | 41257253 | 41257427 | Promoter (<=1kb) | UCHL1 | 308 |
| chr4 | 42397668 | 42398543 | Promoter (<=1kb) | SHISA3 | 0 |
| chr4 | 48490772 | 48491483 | Promoter (<=1kb) | ZAR1 | 520 |
| chr4 | 48985891 | 48986376 | Promoter (<=1kb) | CWH43 | 0 |
| chr4 | 54100169 | 54100481 | Promoter (<=1kb) | GSX2 | 0 |
| chr4 | 55125395 | 55125581 | Promoter (<=1kb) | KDR | 14 |
| chr4 | 55636031 | 55636420 | Promoter (<=1kb) | NMU | 0 |
| chr4 | 56655678 | 56656317 | Promoter (<=1kb) | HOPX | 0 |
| chr4 | 76895714 | 76895992 | Promoter (2-3kb) | SOWAHB | 2155 |
| chr4 | 76897161 | 76898115 | Promoter (<=1kb) | SOWAHB | 32 |
| chr4 | 76948374 | 76948885 | Promoter (<=1kb) | 11-Sep | -818 |
| chr4 | 78551990 | 78552220 | Promoter (<=1kb) | ANXA3 | 232 |
| chr4 | 82451650 | 82451870 | Promoter (<=1kb) | ENOPH1 | 580 |
| chr4 | 84497569 | 84497745 | Promoter (<=1kb) | NKX6-1 | -619 |
| chr4 | 99652973 | 99653197 | Promoter (1-2kb) | C4orf54 | 1451 |
| chr4 | 109557534 | 109557875 | Promoter (2-3kb) | MCUB | -2330 |
| chr4 | 137531441 | 137531653 | Promoter (<=1kb) | PCDH18 | 806 |
| chr4 | 140497790 | 140498059 | Promoter (<=1kb) | MGAT4D | 163 |
| chr4 | 151325100 | 151325414 | Promoter (<=1kb) | SH3D19 | 218 |
| chr4 | 153759811 | 153760017 | Promoter (<=1kb) | RNF175 | 218 |
| chr4 | 153791860 | 153792127 | Promoter (2-3kb) | SFRP2 | -2740 |
| chr4 | 154490634 | 154490844 | Promoter (<=1kb) | DCHS2 | 872 |
| chr4 | 157222238 | 157222476 | Promoter (1-2kb) | GRIA2 | 1474 |
| chr4 | 163331862 | 163332080 | Promoter (<=1kb) | NPY1R | 516 |
| chr4 | 170091248 | 170091483 | Promoter (<=1kb) | AADAT | -861 |
| chr4 | 183798179 | 183798390 | Promoter (<=1kb) | STOX2 | 487 |
| chr5 | 193395 | 193866 | Promoter (1-2kb) | LRRC14B | 1884 |
| chr5 | 301777 | 302106 | Promoter (2-3kb) | AHRR | -2070 |
| chr5 | 302460 | 302838 | Promoter (1-2kb) | AHRR | -1338 |
| chr5 | 843934 | 844131 | Promoter (<=1kb) | ZDHHC11 | -233 |
| chr5 | 1346398 | 1346925 | Promoter (1-2kb) | CLPTM1L | -1299 |
| chr5 | 1881775 | 1882288 | Promoter (<=1kb) | IRX4 | 478 |
| chr5 | 6447539 | 6447828 | Promoter (<=1kb) | UBE2QL1 | -795 |
| chr5 | 7396364 | 7396534 | Promoter (<=1kb) | ADCY2 | 156 |
| chr5 | 32712115 | 32712303 | Promoter (<=1kb) | NPR3 | 392 |
| chr5 | 32713474 | 32713929 | Promoter (1-2kb) | NPR3 | 1751 |
| chr5 | 33938107 | 33938301 | Promoter (1-2kb) | RXFP3 | 1721 |
| chr5 | 37834842 | 37835266 | Promoter (<=1kb) | GDNF | -20 |
| chr5 | 42424804 | 42425093 | Promoter (<=1kb) | GHR | 352 |
| chr5 | 53478551 | 53478790 | Promoter (1-2kb) | FST | -1619 |
| chr5 | 59893611 | 59894148 | Promoter (<=1kb) | PDE4D | 0 |
| chr5 | 63961687 | 63961913 | Promoter (<=1kb) | HTR1A | 220 |
| chr5 | 64690506 | 64691048 | Promoter (<=1kb) | SHISAL2B | 198 |
| chr5 | 73120558 | 73120761 | Promoter (<=1kb) | TMEM171 | 0 |
| chr5 | 76953618 | 76953979 | Promoter (<=1kb) | CRHBP | 569 |
| chr5 | 77209712 | 77209905 | Promoter (<=1kb) | PDE8B | -976 |
| chr5 | 77210693 | 77211140 | Promoter (<=1kb) | PDE8B | 0 |
| chr5 | 79070123 | 79070361 | Promoter (<=1kb) | BHMT2 | 356 |
| chr5 | 80960353 | 80960531 | Promoter (<=1kb) | RASGRF2 | -141 |
| chr5 | 81232967 | 81233339 | Promoter (<=1kb) | CKMT2 | 0 |
| chr5 | 111072595 | 111072806 | Promoter (<=1kb) | TSLP | -508 |
| chr5 | 115179532 | 115179916 | Promoter (<=1kb) | TRIM36 | 121 |
| chr5 | 132813927 | 132814615 | Promoter (<=1kb) | SOWAHA | 340 |
| chr5 | 136193316 | 136193596 | Promoter (<=1kb) | SMIM32 | -182 |
| chr5 | 138274273 | 138274615 | Promoter (<=1kb) | GFRA3 | 0 |
| chr5 | 139392067 | 139392543 | Promoter (2-3kb) | MZB1 | -2153 |
| chr5 | 140631875 | 140632514 | Promoter (1-2kb) | CD14 | 1187 |
| chr5 | 141179379 | 141179956 | Promoter (1-2kb) | PCDHB16 | -1443 |
| chr5 | 141672567 | 141672741 | Promoter (<=1kb) | ARAP3 | 419 |
| chr5 | 146338238 | 146338583 | Promoter (<=1kb) | POU4F3 | -256 |
| chr5 | 149773571 | 149774220 | Promoter (1-2kb) | PPARGC1B | 1631 |
| chr5 | 150302252 | 150302423 | Promoter (<=1kb) | ARSI | 530 |
| chr5 | 150508009 | 150508321 | Promoter (<=1kb) | NDST1 | 0 |
| chr5 | 151087051 | 151087262 | Promoter (<=1kb) | TNIP1 | 0 |
| chr5 | 160230852 | 160231165 | Promoter (1-2kb) | FABP6 | 1422 |
| chr5 | 172185668 | 172185923 | Promoter (2-3kb) | STK10 | 2463 |
| chr5 | 176565305 | 176565588 | Promoter (<=1kb) | CDHR2 | 0 |
| chr5 | 176608945 | 176609163 | Promoter (<=1kb) | GPRIN1 | 970 |
| chr5 | 176812363 | 176812590 | Promoter (1-2kb) | UNC5A | 1844 |
| chr5 | 176899970 | 176900319 | Promoter (<=1kb) | HK3 | -638 |
| chr5 | 178188861 | 178189055 | Promoter (1-2kb) | GMCL2 | -1429 |
| chr5 | 179792417 | 179792684 | Promoter (1-2kb) | LTC4S | -1302 |
| chr5 | 179868624 | 179868836 | Promoter (2-3kb) | TBC1D9B | 2969 |
| chr5 | 180590808 | 180591118 | Promoter (<=1kb) | SCGB3A1 | 422 |
| chr5 | 180649245 | 180649572 | Promoter (<=1kb) | FLT4 | 28 |
| chr5 | 181130846 | 181131133 | Promoter (<=1kb) | OR2V1 | 36 |
| chr6 | 391838 | 392125 | Promoter (<=1kb) | IRF4 | 99 |
| chr6 | 656501 | 656780 | Promoter (<=1kb) | HUS1B | 183 |
| chr6 | 2903183 | 2903431 | Promoter (<=1kb) | SERPINB9 | 0 |
| chr6 | 3005849 | 3006232 | Promoter (1-2kb) | NQO2 | 1682 |
| chr6 | 4079032 | 4079666 | Promoter (<=1kb) | C6orf201 | 0 |
| chr6 | 5084550 | 5084938 | Promoter (<=1kb) | PPP1R3G | 0 |
| chr6 | 6320093 | 6320416 | Promoter (<=1kb) | F13A1 | 418 |
| chr6 | 7229221 | 7229405 | Promoter (1-2kb) | RREB1 | -1237 |
| chr6 | 7230265 | 7230602 | Promoter (<=1kb) | RREB1 | -40 |
| chr6 | 7542347 | 7542674 | Promoter (<=1kb) | DSP | 730 |
| chr6 | 10416818 | 10417106 | Promoter (<=1kb) | TFAP2A | -628 |
| chr6 | 10419492 | 10419984 | Promoter (<=1kb) | TFAP2A | 0 |
| chr6 | 26188787 | 26189099 | Promoter (<=1kb) | HIST1H4D | 0 |
| chr6 | 27139193 | 27139598 | Promoter (<=1kb) | HIST1H4I | 605 |
| chr6 | 28261840 | 28262228 | Promoter (2-3kb) | NKAPL | 2520 |
| chr6 | 28399359 | 28399808 | Promoter (<=1kb) | ZSCAN12 | 0 |
| chr6 | 29553230 | 29553991 | Promoter (2-3kb) | OR2I1P | 2823 |
| chr6 | 29588060 | 29588685 | Promoter (<=1kb) | OR2H2 | 605 |
| chr6 | 29680933 | 29681111 | Promoter (<=1kb) | ZFP57 | 0 |
| chr6 | 31114582 | 31114754 | Promoter (<=1kb) | PSORS1C1 | -77 |
| chr6 | 31171219 | 31171447 | Promoter (<=1kb) | POU5F1 | -526 |
| chr6 | 31355580 | 31355869 | Promoter (<=1kb) | HLA-B | -176 |
| chr6 | 31572302 | 31572676 | Promoter (<=1kb) | LTA | 19 |
| chr6 | 31671062 | 31671285 | Promoter (<=1kb) | LY6G5B | 683 |
| chr6 | 31928068 | 31928248 | Promoter (<=1kb) | AL645922.1 | 344 |
| chr6 | 31946365 | 31946675 | Promoter (<=1kb) | CFB | 656 |
| chr6 | 32046787 | 32047071 | Promoter (<=1kb) | TNXB | -659 |
| chr6 | 32166751 | 32166955 | Promoter (2-3kb) | EGFL8 | 2146 |
| chr6 | 32167272 | 32167441 | Promoter (2-3kb) | EGFL8 | 2667 |
| chr6 | 32761430 | 32761635 | Promoter (1-2kb) | HLA-DQB2 | 1855 |
| chr6 | 32761996 | 32762193 | Promoter (1-2kb) | HLA-DQB2 | 1297 |
| chr6 | 34099192 | 34099725 | Promoter (2-3kb) | GRM4 | 2426 |
| chr6 | 34515773 | 34516140 | Promoter (<=1kb) | PACSIN1 | 901 |
| chr6 | 35737483 | 35737685 | Promoter (<=1kb) | ARMC12 | 396 |
| chr6 | 35797083 | 35797356 | Promoter (<=1kb) | CLPS | 0 |
| chr6 | 36839431 | 36839644 | Promoter (<=1kb) | CPNE5 | 358 |
| chr6 | 36947819 | 36948281 | Promoter (<=1kb) | PI16 | 0 |
| chr6 | 39315127 | 39315307 | Promoter (<=1kb) | KCNK17 | -574 |
| chr6 | 40585799 | 40586391 | Promoter (1-2kb) | LRFN2 | 1074 |
| chr6 | 41161109 | 41161470 | Promoter (1-2kb) | TREM2 | 1633 |
| chr6 | 41200125 | 41200550 | Promoter (<=1kb) | TREML2 | 644 |
| chr6 | 41201070 | 41201305 | Promoter (<=1kb) | TREML2 | 0 |
| chr6 | 41336053 | 41336235 | Promoter (<=1kb) | NCR2 | 202 |
| chr6 | 41888303 | 41888506 | Promoter (<=1kb) | USP49 | -974 |
| chr6 | 42142379 | 42142794 | Promoter (<=1kb) | C6orf132 | 0 |
| chr6 | 43246793 | 43247068 | Promoter (<=1kb) | TTBK1 | -921 |
| chr6 | 45422666 | 45423850 | Promoter (<=1kb) | RUNX2 | 89 |
| chr6 | 52087873 | 52088221 | Promoter (<=1kb) | PKHD1 | -248 |
| chr6 | 54846972 | 54847146 | Promoter (<=1kb) | FAM83B | 201 |
| chr6 | 55579115 | 55579369 | Promoter (<=1kb) | HMGCLL1 | 0 |
| chr6 | 71888119 | 71888316 | Promoter (1-2kb) | RIMS1 | 1095 |
| chr6 | 73451517 | 73451972 | Promoter (<=1kb) | CGAS | 287 |
| chr6 | 90296333 | 90296578 | Promoter (<=1kb) | BACH2 | 164 |
| chr6 | 107489653 | 107489977 | Promoter (<=1kb) | SOBP | 0 |
| chr6 | 109713626 | 109713936 | Promoter (2-3kb) | FIG4 | -2570 |
| chr6 | 113859480 | 113859895 | Promoter (2-3kb) | MARCKS | 2118 |
| chr6 | 132401045 | 132401625 | Promoter (<=1kb) | MOXD1 | 0 |
| chr6 | 149919909 | 149920086 | Promoter (2-3kb) | RAET1G | 2992 |
| chr6 | 149923007 | 149923176 | Promoter (<=1kb) | RAET1G | 0 |
| chr6 | 150143167 | 150143375 | Promoter (<=1kb) | PPP1R14C | 91 |
| chr6 | 151690982 | 151691260 | Promoter (<=1kb) | ESR1 | 486 |
| chr6 | 158536903 | 158537894 | Promoter (<=1kb) | TMEM181 | 467 |
| chr6 | 158642315 | 158642586 | Promoter (2-3kb) | DYNLT1 | 2153 |
| chr6 | 160348224 | 160348434 | Promoter (<=1kb) | SLC22A3 | 0 |
| chr6 | 165662846 | 165663158 | Promoter (<=1kb) | PDE10A | 87 |
| chr6 | 166307981 | 166308485 | Promoter (<=1kb) | PRR18 | 0 |
| chr6 | 166955625 | 166955875 | Promoter (<=1kb) | AL159163.1 | 249 |
| chr6 | 168655630 | 168655961 | Promoter (2-3kb) | SMOC2 | 2671 |
| chr6 | 170295650 | 170295875 | Promoter (<=1kb) | FAM120B | 329 |
| chr7 | 712134 | 712538 | Promoter (<=1kb) | PRKAR1B | 34 |
| chr7 | 1052230 | 1052468 | Promoter (2-3kb) | GPR146 | -2817 |
| chr7 | 1092718 | 1092931 | Promoter (<=1kb) | GPER1 | -7 |
| chr7 | 1707346 | 1707528 | Promoter (1-2kb) | ELFN1 | -1634 |
| chr7 | 1711790 | 1711978 | Promoter (2-3kb) | ELFN1 | 2628 |
| chr7 | 2516323 | 2516537 | Promoter (1-2kb) | LFNG | -1326 |
| chr7 | 6271958 | 6272182 | Promoter (<=1kb) | CYTH3 | 462 |
| chr7 | 18086429 | 18086876 | Promoter (<=1kb) | HDAC9 | -73 |
| chr7 | 20646985 | 20647218 | Promoter (<=1kb) | ABCB5 | -125 |
| chr7 | 21945616 | 21946214 | Promoter (<=1kb) | CDCA7L | 0 |
| chr7 | 27173983 | 27174151 | Promoter (<=1kb) | HOXA10 | 104 |
| chr7 | 30921939 | 30922285 | Promoter (<=1kb) | AQP1 | 449 |
| chr7 | 44540959 | 44541318 | Promoter (<=1kb) | NPC1L1 | 0 |
| chr7 | 44755619 | 44756516 | Promoter (<=1kb) | ZMIZ2 | 0 |
| chr7 | 47581524 | 47581912 | Promoter (<=1kb) | TNS3 | 232 |
| chr7 | 70695235 | 70695430 | Promoter (1-2kb) | AUTS2 | 1177 |
| chr7 | 72335982 | 72336270 | Promoter (<=1kb) | CALN1 | 725 |
| chr7 | 73306914 | 73307136 | Promoter (1-2kb) | NSUN5 | 1668 |
| chr7 | 73769882 | 73770235 | Promoter (<=1kb) | CLDN3 | 35 |
| chr7 | 74287083 | 74287270 | Promoter (2-3kb) | CLIP2 | -2205 |
| chr7 | 74290245 | 74290550 | Promoter (<=1kb) | CLIP2 | 770 |
| chr7 | 75813494 | 75813724 | Promoter (<=1kb) | CCL24 | 82 |
| chr7 | 75980929 | 75981101 | Promoter (<=1kb) | POR | -810 |
| chr7 | 76423346 | 76423709 | Promoter (1-2kb) | ZP3 | -1198 |
| chr7 | 76430800 | 76431542 | Promoter (2-3kb) | ZP3 | 2162 |
| chr7 | 87475564 | 87475967 | Promoter (<=1kb) | ABCB4 | 0 |
| chr7 | 98869319 | 98869892 | Promoter (<=1kb) | TMEM130 | 0 |
| chr7 | 100012499 | 100012785 | Promoter (2-3kb) | ZKSCAN1 | -2787 |
| chr7 | 100166771 | 100166966 | Promoter (<=1kb) | GAL3ST4 | 264 |
| chr7 | 100221430 | 100222047 | Promoter (2-3kb) | PVRIG | 2194 |
| chr7 | 100304768 | 100304967 | Promoter (2-3kb) | SPDYE3 | -2735 |
| chr7 | 100372498 | 100373055 | Promoter (<=1kb) | PILRA | -390 |
| chr7 | 100488960 | 100489162 | Promoter (2-3kb) | NYAP1 | 2036 |
| chr7 | 100599937 | 100600337 | Promoter (1-2kb) | PCOLCE | -1840 |
| chr7 | 100641508 | 100642218 | Promoter (<=1kb) | TFR2 | 0 |
| chr7 | 100951757 | 100951981 | Promoter (2-3kb) | MUC3A | 2132 |
| chr7 | 101180078 | 101180478 | Promoter (<=1kb) | NAT16 | 0 |
| chr7 | 101202098 | 101202411 | Promoter (1-2kb) | MOGAT3 | -1077 |
| chr7 | 102287710 | 102287885 | Promoter (2-3kb) | SH2B2 | 2555 |
| chr7 | 102910640 | 102910811 | Promoter (2-3kb) | LRRC17 | -2180 |
| chr7 | 104328898 | 104329229 | Promoter (<=1kb) | LHFPL3 | 198 |
| chr7 | 111561489 | 111561774 | Promoter (<=1kb) | IMMP2L | 150 |
| chr7 | 116525228 | 116525430 | Promoter (<=1kb) | CAV1 | 219 |
| chr7 | 120273838 | 120274162 | Promoter (<=1kb) | KCND2 | 170 |
| chr7 | 127593707 | 127593937 | Promoter (<=1kb) | FSCN3 | 72 |
| chr7 | 128241150 | 128241436 | Promoter (<=1kb) | LEP | 0 |
| chr7 | 128715576 | 128715822 | Promoter (<=1kb) | FAM71F1 | 187 |
| chr7 | 130486497 | 130486704 | Promoter (<=1kb) | MEST | 155 |
| chr7 | 132647344 | 132647602 | Promoter (1-2kb) | PLXNA4 | 1086 |
| chr7 | 138776479 | 138776656 | Promoter (2-3kb) | ATP6V0A4 | -2442 |
| chr7 | 139482683 | 139483957 | Promoter (<=1kb) | KLRG2 | 0 |
| chr7 | 139524193 | 139524436 | Promoter (<=1kb) | CLEC2L | 337 |
| chr7 | 141073894 | 141074331 | Promoter (<=1kb) | TMEM178B | 0 |
| chr7 | 151167476 | 151167738 | Promoter (<=1kb) | GBX1 | 0 |
| chr7 | 151187096 | 151187509 | Promoter (<=1kb) | ASB10 | 0 |
| chr7 | 151273960 | 151274172 | Promoter (2-3kb) | SMARCD3 | 2562 |
| chr7 | 151275988 | 151276251 | Promoter (<=1kb) | SMARCD3 | 483 |
| chr7 | 151409786 | 151410035 | Promoter (<=1kb) | WDR86 | 0 |
| chr7 | 151410387 | 151410580 | Promoter (<=1kb) | WDR86 | 125 |
| chr7 | 151440015 | 151440834 | Promoter (<=1kb) | CRYGN | 0 |
| chr7 | 153886144 | 153886464 | Promoter (<=1kb) | DPP6 | -633 |
| chr8 | 1860330 | 1860630 | Promoter (1-2kb) | ARHGEF10 | 1575 |
| chr8 | 6877252 | 6877544 | Promoter (<=1kb) | DEFB1 | 478 |
| chr8 | 8702868 | 8703070 | Promoter (<=1kb) | CLDN23 | 930 |
| chr8 | 8891658 | 8891838 | Promoter (1-2kb) | MFHAS1 | 1807 |
| chr8 | 10061947 | 10062169 | Promoter (2-3kb) | MSRA | 2977 |
| chr8 | 10524043 | 10524275 | Promoter (1-2kb) | PRSS55 | -1271 |
| chr8 | 11794630 | 11795151 | Promoter (<=1kb) | FDFT1 | -422 |
| chr8 | 17412967 | 17413494 | Promoter (<=1kb) | MTMR7 | 0 |
| chr8 | 17674565 | 17674807 | Promoter (1-2kb) | MTUS1 | 1686 |
| chr8 | 19939580 | 19940302 | Promoter (<=1kb) | LPL | 327 |
| chr8 | 22045315 | 22045484 | Promoter (2-3kb) | FGF17 | 2889 |
| chr8 | 22927681 | 22928493 | Promoter (<=1kb) | PEBP4 | 0 |
| chr8 | 23161069 | 23161405 | Promoter (2-3kb) | TNFRSF10D | 2625 |
| chr8 | 23403320 | 23403988 | Promoter (<=1kb) | LOXL2 | 252 |
| chr8 | 24913691 | 24914234 | Promoter (<=1kb) | NEFM | 0 |
| chr8 | 26864387 | 26864743 | Promoter (<=1kb) | ADRA1A | 233 |
| chr8 | 27989733 | 27990040 | Promoter (2-3kb) | SCARA5 | -2118 |
| chr8 | 30641453 | 30641829 | Promoter (1-2kb) | SMIM18 | -1651 |
| chr8 | 30886345 | 30886566 | Promoter (2-3kb) | TEX15 | 2140 |
| chr8 | 37797813 | 37798079 | Promoter (<=1kb) | ADGRA2 | 557 |
| chr8 | 38821866 | 38822058 | Promoter (1-2kb) | TACC1 | 1319 |
| chr8 | 38900854 | 38901564 | Promoter (<=1kb) | PLEKHA2 | 0 |
| chr8 | 41664922 | 41665269 | Promoter (<=1kb) | ANK1 | 0 |
| chr8 | 41797418 | 41797657 | Promoter (<=1kb) | ANK1 | 0 |
| chr8 | 41798722 | 41799015 | Promoter (1-2kb) | ANK1 | -1100 |
| chr8 | 52565340 | 52565842 | Promoter (<=1kb) | ALKAL1 | 0 |
| chr8 | 53251502 | 53251735 | Promoter (<=1kb) | OPRK1 | 0 |
| chr8 | 56319996 | 56320281 | Promoter (<=1kb) | SDR16C5 | 0 |
| chr8 | 56321342 | 56321544 | Promoter (<=1kb) | SDR16C5 | -566 |
| chr8 | 61648742 | 61649240 | Promoter (1-2kb) | ASPH | -1935 |
| chr8 | 63087593 | 63087834 | Promoter (1-2kb) | TTPA | -1540 |
| chr8 | 67951826 | 67952352 | Promoter (<=1kb) | PREX2 | 0 |
| chr8 | 70069766 | 70069969 | Promoter (1-2kb) | PRDM14 | 1358 |
| chr8 | 70403899 | 70404212 | Promoter (<=1kb) | NCOA2 | -94 |
| chr8 | 73092788 | 73093425 | Promoter (<=1kb) | SBSPON | 0 |
| chr8 | 74823466 | 74823668 | Promoter (<=1kb) | PI15 | -866 |
| chr8 | 76678057 | 76678233 | Promoter (2-3kb) | ZFHX4 | -2986 |
| chr8 | 85438746 | 85438993 | Promoter (<=1kb) | CA3 | 0 |
| chr8 | 94640361 | 94640584 | Promoter (<=1kb) | ESRP1 | -490 |
| chr8 | 94641660 | 94641838 | Promoter (<=1kb) | ESRP1 | 456 |
| chr8 | 96159703 | 96159872 | Promoter (<=1kb) | GDF6 | 844 |
| chr8 | 96494920 | 96495300 | Promoter (<=1kb) | SDC2 | 912 |
| chr8 | 97277503 | 97277977 | Promoter (<=1kb) | TSPYL5 | 0 |
| chr8 | 98426256 | 98426427 | Promoter (<=1kb) | KCNS2 | -595 |
| chr8 | 98427956 | 98428158 | Promoter (<=1kb) | KCNS2 | 425 |
| chr8 | 100157953 | 100158289 | Promoter (<=1kb) | SPAG1 | 0 |
| chr8 | 102123917 | 102124701 | Promoter (<=1kb) | NCALD | 0 |
| chr8 | 116949835 | 116950116 | Promoter (<=1kb) | SLC30A8 | -157 |
| chr8 | 120124944 | 120125143 | Promoter (<=1kb) | COL14A1 | 0 |
| chr8 | 123540285 | 123540794 | Promoter (<=1kb) | FBXO32 | 412 |
| chr8 | 132480855 | 132481105 | Promoter (<=1kb) | KCNQ3 | 0 |
| chr8 | 132760604 | 132760777 | Promoter (<=1kb) | TMEM71 | -22 |
| chr8 | 142750288 | 142750520 | Promoter (2-3kb) | LYPD2 | 2014 |
| chr8 | 142772785 | 142773385 | Promoter (2-3kb) | LYNX1-SLURP2 | 2636 |
| chr8 | 142774072 | 142774403 | Promoter (1-2kb) | LYNX1-SLURP2 | 1618 |
| chr8 | 143080646 | 143081339 | Promoter (<=1kb) | LY6L | 189 |
| chr8 | 144239651 | 144240198 | Promoter (<=1kb) | MROH1 | 539 |
| chr8 | 144353862 | 144354059 | Promoter (<=1kb) | TMEM249 | 855 |
| chr8 | 144474597 | 144474965 | Promoter (1-2kb) | FOXH1 | 1370 |
| chr8 | 144605892 | 144606256 | Promoter (<=1kb) | ARHGAP39 | -76 |
| chr9 | 504459 | 504773 | Promoter (<=1kb) | KANK1 | 0 |
| chr9 | 976470 | 976844 | Promoter (<=1kb) | DMRT3 | -120 |
| chr9 | 32782920 | 32783257 | Promoter (<=1kb) | TMEM215 | -242 |
| chr9 | 34522196 | 34522458 | Promoter (<=1kb) | ENHO | 583 |
| chr9 | 34654542 | 34654790 | Promoter (<=1kb) | IL11RA | -428 |
| chr9 | 35563189 | 35563498 | Promoter (<=1kb) | FAM166B | 330 |
| chr9 | 35615807 | 35616149 | Promoter (2-3kb) | CD72 | 2221 |
| chr9 | 35618561 | 35618761 | Promoter (<=1kb) | CD72 | 50 |
| chr9 | 37032922 | 37033110 | Promoter (<=1kb) | PAX5 | 921 |
| chr9 | 69325744 | 69325923 | Promoter (<=1kb) | FAM189A2 | 522 |
| chr9 | 69671353 | 69671568 | Promoter (<=1kb) | APBA1 | 738 |
| chr9 | 86259207 | 86259465 | Promoter (<=1kb) | C9orf153 | 139 |
| chr9 | 89177709 | 89178007 | Promoter (<=1kb) | SHC3 | 760 |
| chr9 | 89362070 | 89362296 | Promoter (2-3kb) | SEMA4D | 2355 |
| chr9 | 92949422 | 92949653 | Promoter (1-2kb) | FGD3 | 1971 |
| chr9 | 93058782 | 93059074 | Promoter (<=1kb) | SUSD3 | 75 |
| chr9 | 93071829 | 93072344 | Promoter (2-3kb) | SUSD3 | 2729 |
| chr9 | 93345615 | 93346081 | Promoter (<=1kb) | C9orf129 | 333 |
| chr9 | 94639273 | 94639604 | Promoter (<=1kb) | FBP1 | 0 |
| chr9 | 98171610 | 98171827 | Promoter (1-2kb) | CORO2A | 1057 |
| chr9 | 99828831 | 99829017 | Promoter (2-3kb) | NR4A3 | 2104 |
| chr9 | 111038577 | 111038987 | Promoter (<=1kb) | LPAR1 | -492 |
| chr9 | 113463501 | 113463979 | Promoter (<=1kb) | RGS3 | 0 |
| chr9 | 114157132 | 114157350 | Promoter (1-2kb) | COL27A1 | 1572 |
| chr9 | 114348143 | 114348347 | Promoter (<=1kb) | AKNA | 652 |
| chr9 | 117414228 | 117414417 | Promoter (<=1kb) | ASTN2 | 622 |
| chr9 | 120876154 | 120876343 | Promoter (<=1kb) | PHF19 | 845 |
| chr9 | 121299525 | 121299873 | Promoter (<=1kb) | GSN | 0 |
| chr9 | 122261601 | 122261805 | Promoter (2-3kb) | MRRF | -2798 |
| chr9 | 122800480 | 122800935 | Promoter (<=1kb) | OR1K1 | 357 |
| chr9 | 123357606 | 123357834 | Promoter (1-2kb) | CRB2 | 1346 |
| chr9 | 124802508 | 124802737 | Promoter (1-2kb) | OLFML2A | 1634 |
| chr9 | 127579443 | 127579622 | Promoter (<=1kb) | STXBP1 | 73 |
| chr9 | 127705836 | 127706132 | Promoter (<=1kb) | CFAP157 | -860 |
| chr9 | 127706925 | 127707270 | Promoter (<=1kb) | CFAP157 | 0 |
| chr9 | 127853633 | 127853811 | Promoter (<=1kb) | ENG | 825 |
| chr9 | 128146248 | 128146765 | Promoter (2-3kb) | LCN2 | -2306 |
| chr9 | 128260140 | 128260434 | Promoter (<=1kb) | GOLGA2 | 126 |
| chr9 | 128941154 | 128941518 | Promoter (<=1kb) | AL672142.1 | 0 |
| chr9 | 129665303 | 129665542 | Promoter (<=1kb) | PRRX2 | -99 |
| chr9 | 129665739 | 129665957 | Promoter (<=1kb) | PRRX2 | 98 |
| chr9 | 130173586 | 130173757 | Promoter (1-2kb) | NCS1 | 1008 |
| chr9 | 130304361 | 130304787 | Promoter (2-3kb) | HMCN2 | -2732 |
| chr9 | 130896370 | 130896901 | Promoter (<=1kb) | QRFP | 0 |
| chr9 | 130898081 | 130898566 | Promoter (1-2kb) | QRFP | -1269 |
| chr9 | 131277121 | 131277482 | Promoter (<=1kb) | FAM78A | -574 |
| chr9 | 131290397 | 131290685 | Promoter (<=1kb) | PLPP7 | 585 |
| chr9 | 132582520 | 132582729 | Promoter (<=1kb) | BARHL1 | 0 |
| chr9 | 133274846 | 133275360 | Promoter (<=1kb) | ABO | 0 |
| chr9 | 133454480 | 133454702 | Promoter (<=1kb) | ADAMTS13 | -946 |
| chr9 | 135548161 | 135548486 | Promoter (2-3kb) | OBP2A | 2006 |
| chr9 | 135548797 | 135549035 | Promoter (2-3kb) | OBP2A | 2642 |
| chr9 | 135561425 | 135561807 | Promoter (<=1kb) | PAEP | 0 |
| chr9 | 136476602 | 136476984 | Promoter (<=1kb) | SEC16A | 97 |
| chr9 | 136994356 | 136994795 | Promoter (1-2kb) | PAXX | 1938 |
| chr9 | 137139020 | 137139477 | Promoter (<=1kb) | GRIN1 | -4 |
| chrX | 2829623 | 2829862 | Promoter (<=1kb) | GYG2 | -327 |
| chrX | 23766140 | 23766439 | Promoter (<=1kb) | ACOT9 | 36 |
| chrX | 30308441 | 30308659 | Promoter (<=1kb) | NR0B1 | 0 |
| chrX | 47209897 | 47210231 | Promoter (1-2kb) | UBA1 | 1335 |
| chrX | 48786227 | 48786467 | Promoter (<=1kb) | GATA1 | -87 |
| chrX | 53048762 | 53048937 | Promoter (<=1kb) | GPR173 | -154 |
| chrX | 71911015 | 71911214 | Promoter (<=1kb) | NHSL2 | 197 |
| chrX | 72178847 | 72179062 | Promoter (2-3kb) | PIN4 | -2614 |
| chrX | 75274581 | 75274792 | Promoter (<=1kb) | UPRT | 0 |
| chrX | 76429063 | 76429285 | Promoter (1-2kb) | MAGEE1 | 1339 |
| chrX | 102651137 | 102651472 | Promoter (<=1kb) | GPRASP1 | 0 |
| chrX | 118976083 | 118976259 | Promoter (<=1kb) | LONRF3 | -73 |
| chrX | 129654795 | 129655084 | Promoter (<=1kb) | APLN | 0 |
| chrX | 140505598 | 140505845 | Promoter (<=1kb) | SOX3 | -482 |
| chrX | 153400297 | 153400622 | Promoter (<=1kb) | PNMA6E | 770 |
| chrX | 153449505 | 153449716 | Promoter (1-2kb) | TREX2 | -1642 |
| chrX | 153946707 | 153946981 | Promoter (2-3kb) | RENBP | -2016 |
| chrX | 154459507 | 154459920 | Promoter (1-2kb) | PLXNA3 | 1226 |
| chrX | 154750477 | 154750957 | Promoter (<=1kb) | GAB3 | 100 |
