# Supplementary material for: Metformin promotes histone deacetylation of optineurin and suppresses tumour growth through autophagy inhibition in ocular melanoma
Source: Clin Transl Med. 2022 Jan 24;12(1):e660. doi: 10.1002/ctm2.660 (PMC8787022; doi:10.1002/ctm2.660)

Fig S1

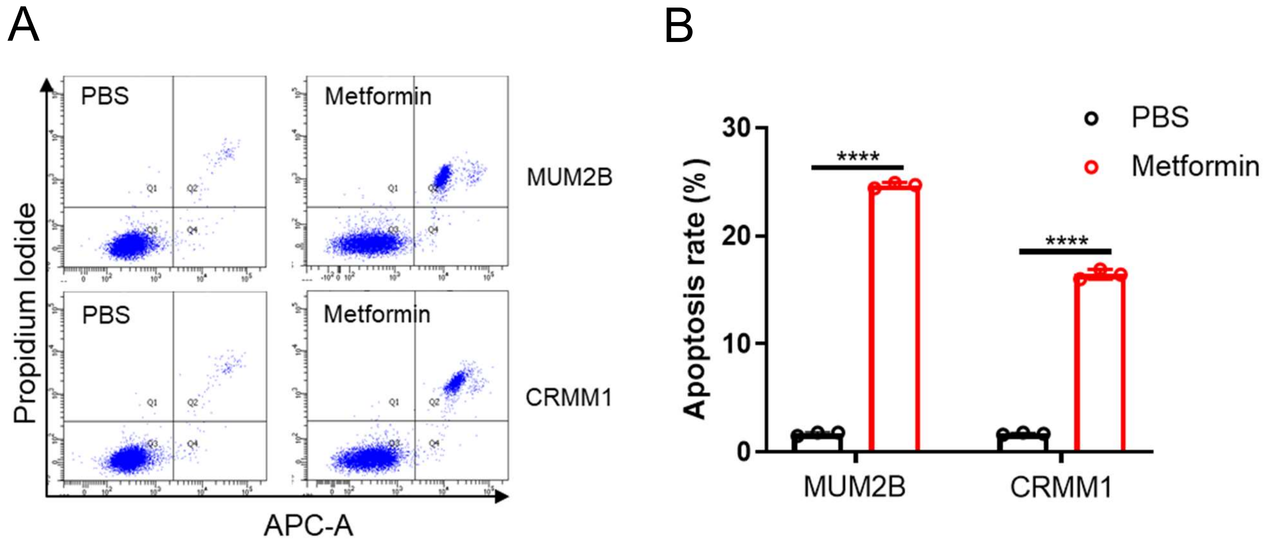
A
PBS
Metformin
MUM2B
PBS
Metformin
CRMM1
Propidium Iodide
APC-A
B
PBS
Metformin
30
20
10
0
Apoptosis rate (%)
****
****
MUM2B
CRMM1

Fig S2

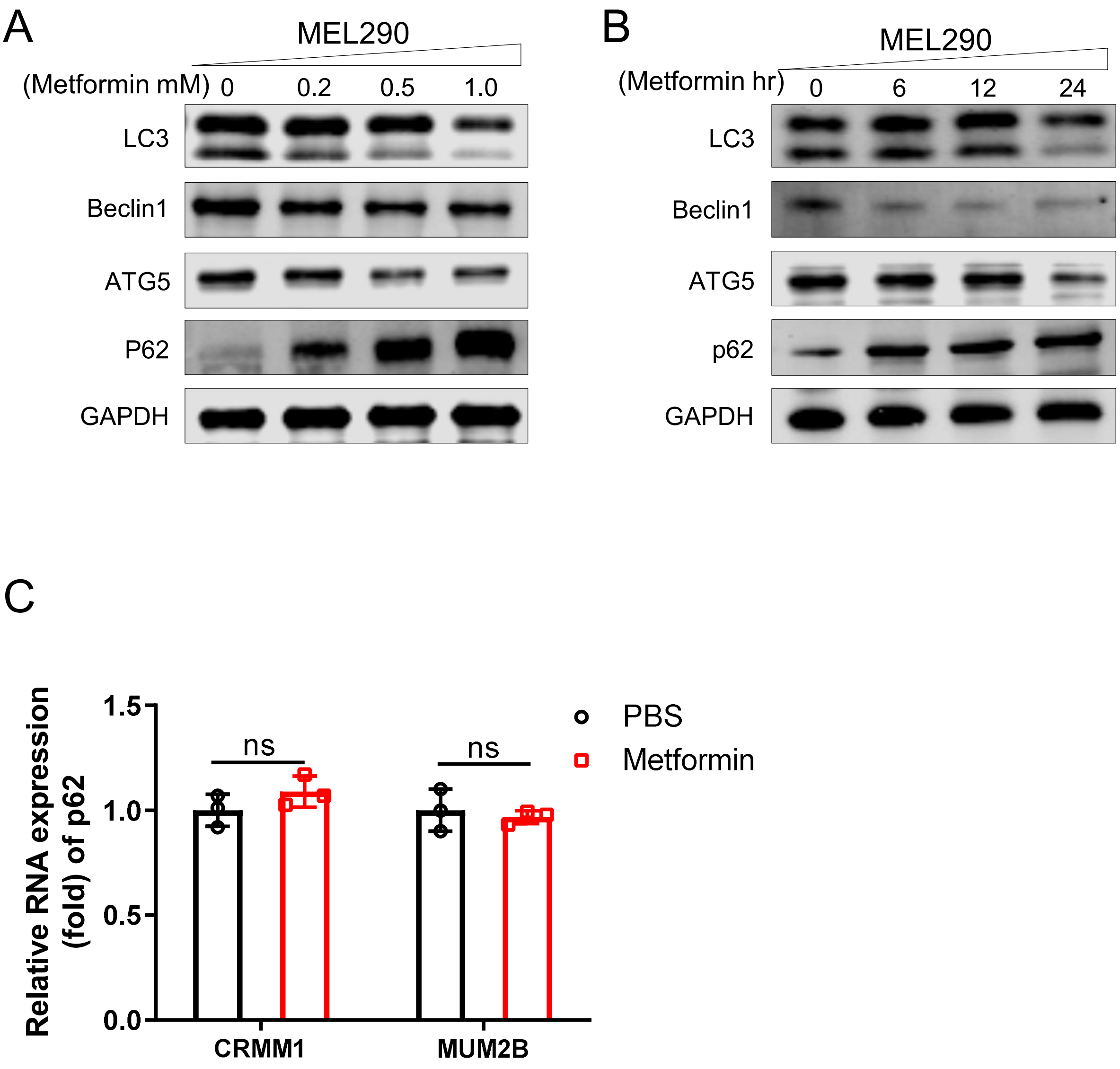
A
MEL290
(Metformin mM)
0
0.2
0.5
1.0
LC3
Beclin1
ATG5
P62
GAPDH
B
MEL290
(Metformin hr)
0
6
12
24
LC3
Beclin1
ATG5
p62
GAPDH
C
Relative RNA expression (fold) of p62
1.5
1.0
0.5
0.0
ns
ns
CRMM1
MUM2B
PBS
Metformin

Fig S3

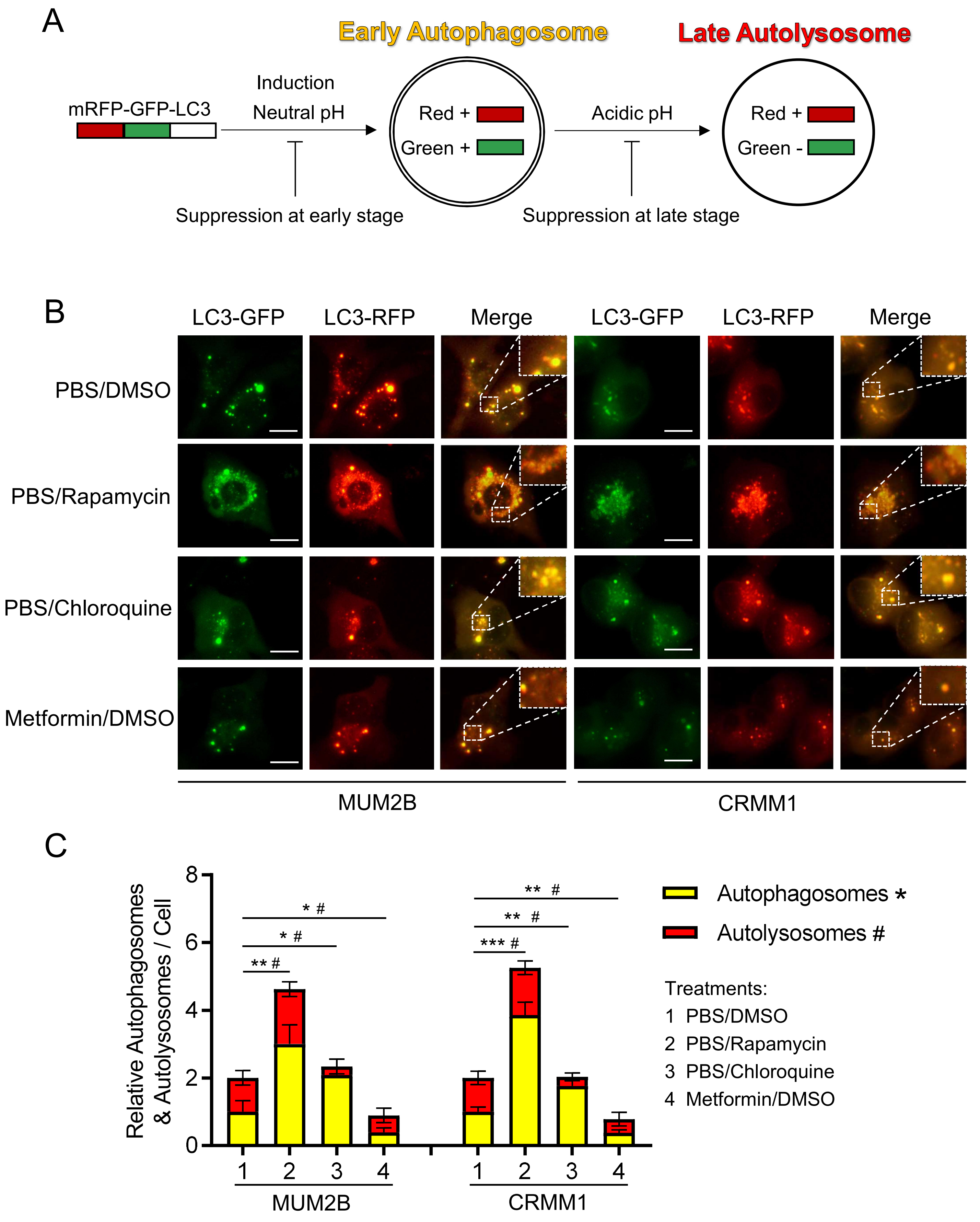
A
Early Autophagosome
Late Autolysosome
mRFP-GFP-LC3
Induction
Neutral pH
Red +
Green +
Acidic pH
Red +
Green -
Suppression at early stage
Suppression at late stage
B
LC3-GFP
LC3-RFP
Merge
LC3-GFP
LC3-RFP
Merge
PBS/DMSO
PBS/Rapamycin
PBS/Chloroquine
Metformin/DMSO
MUM2B
CRMM1
C
Relative Autophagosomes & Autolysosomes / Cell
8
6
4
2
0
** #
* #
* #
*** #
** #
** #
1 2 3 4
MUM2B
1 2 3 4
CRMM1
Autophagosomes *
Autolysosomes #
Treatments:
1 PBS/DMSO
2 PBS/Rapamycin
3 PBS/Chloroquine
4 Metformin/DMSO

Fig S4

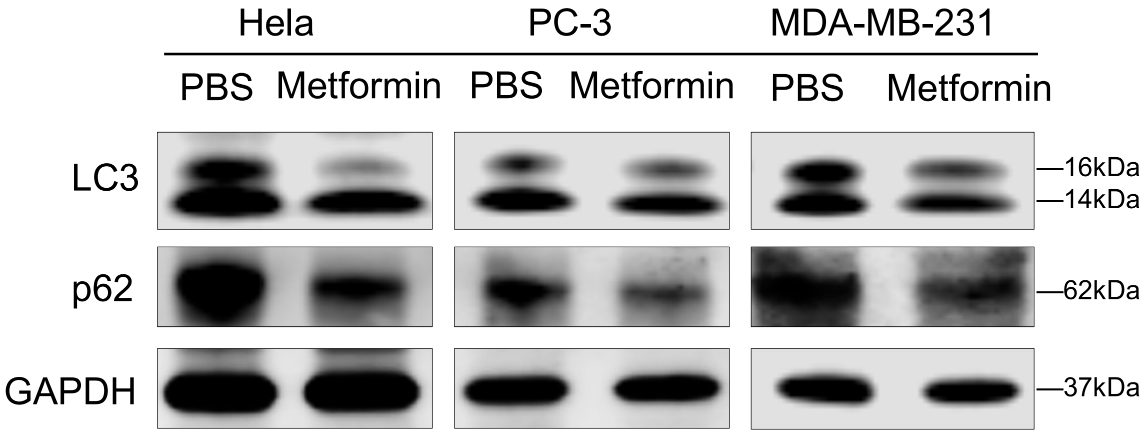
Hela
PC-3
MDA-MB-231
PBS
Metformin
PBS
Metformin
PBS
Metformin
LC3
p62
GAPDH
16kDa
14kDa
62kDa
37kDa

Fig S5

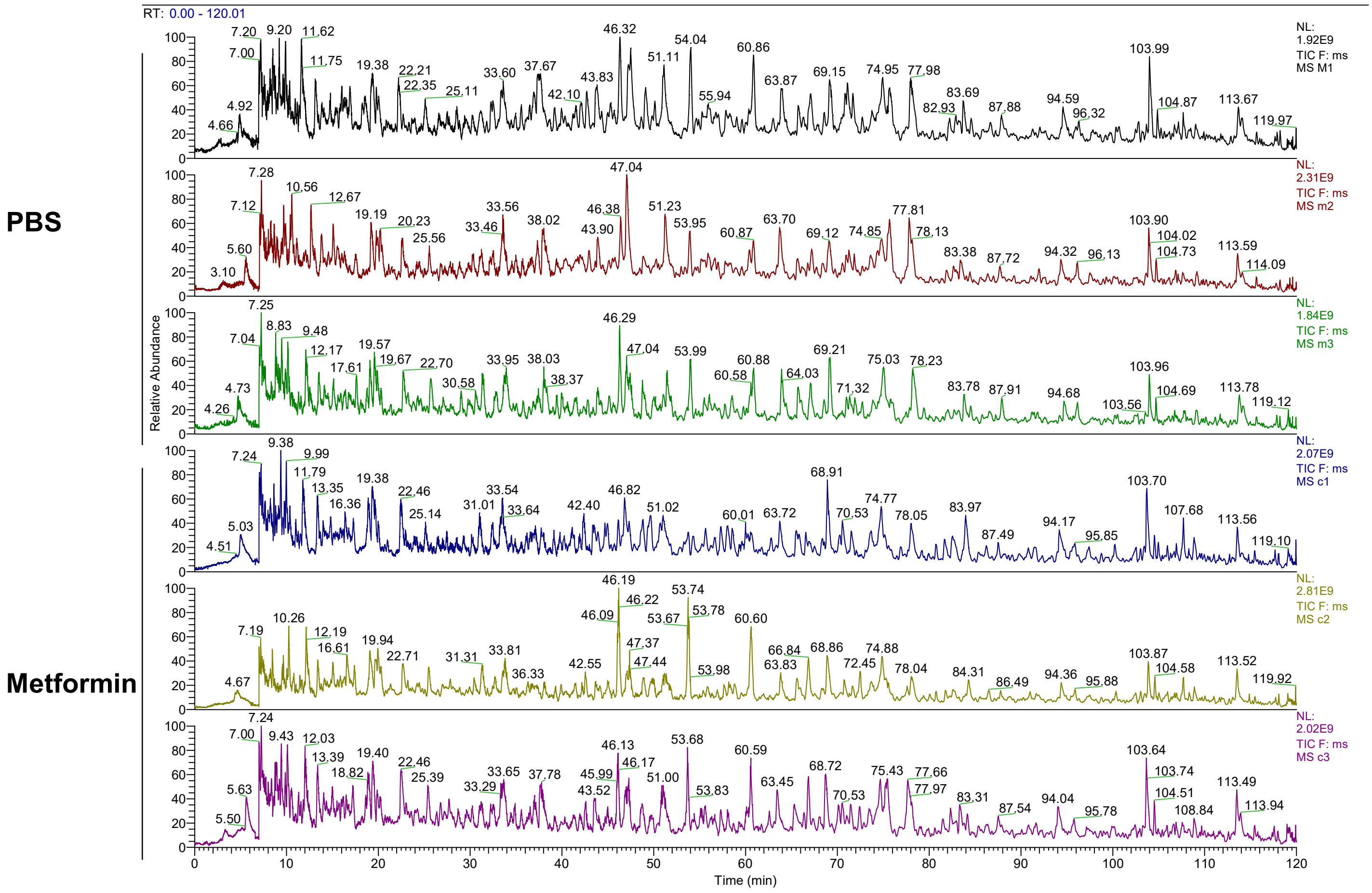

RT: 0.00 - 120.01
PBS
Metformin
Relative Abundance
Time (min)
NL: 1.92E9 TIC F: ms MS M1
7.20 9.20 11.62 7.00 11.75 19.38 22.21 22.35 25.11 33.60 37.67 42.10 43.83 46.32 51.11 54.04 55.94 60.86 63.87 69.15 74.95 77.98 82.93 83.69 87.88 94.59 96.32 103.99 104.87 113.67 119.97 4.92 4.66
NL: 2.31E9 TIC F: ms MS m2
7.28 10.56 12.67 7.12 19.19 20.23 25.56 33.46 33.56 38.02 43.90 46.38 47.04 51.23 53.95 60.87 63.70 69.12 74.85 77.81 78.13 83.38 87.72 94.32 96.13 103.90 104.02 104.73 113.59 114.09 5.60 3.10
NL: 1.84E9 TIC F: ms MS m3
7.25 8.83 9.48 7.04 12.17 17.61 19.57 19.67 22.70 30.58 33.95 38.03 38.37 46.29 47.04 53.99 60.58 60.88 64.03 69.21 71.32 75.03 78.23 83.78 87.91 94.68 103.56 103.96 104.69 113.78 119.12 4.73 4.26
NL: 2.07E9 TIC F: ms MS c1
9.38 7.24 9.99 11.79 13.35 16.36 19.38 22.46 25.14 31.01 33.54 33.64 42.40 46.82 51.02 60.01 63.72 68.91 70.53 74.77 78.05 83.97 87.49 94.17 95.85 103.70 107.68 113.56 119.10 5.03 4.51
NL: 2.81E9 TIC F: ms MS c2
46.19 46.22 46.09 53.74 53.67 53.78 60.60 7.19 10.26 12.19 16.61 19.94 22.71 31.31 33.81 36.33 42.55 47.37 47.44 53.98 66.84 63.83 68.86 72.45 74.88 78.04 84.31 86.49 94.36 95.88 103.87 104.58 113.52 119.92 4.67
NL: 2.02E9 TIC F: ms MS c3
7.24 7.00 9.43 12.03 13.39 18.82 19.40 22.46 25.39 33.29 33.65 37.78 43.52 45.99 46.13 46.17 51.00 53.68 53.83 60.59 63.45 68.72 70.53 75.43 77.66 77.97 83.31 87.54 94.04 95.78 103.64 103.74 104.51 108.84 113.49 113.94 5.63 5.50
0 10 20 30 40 50 60 70 80 90 100 110 120
0 20 40 60 80 100

Fig S6

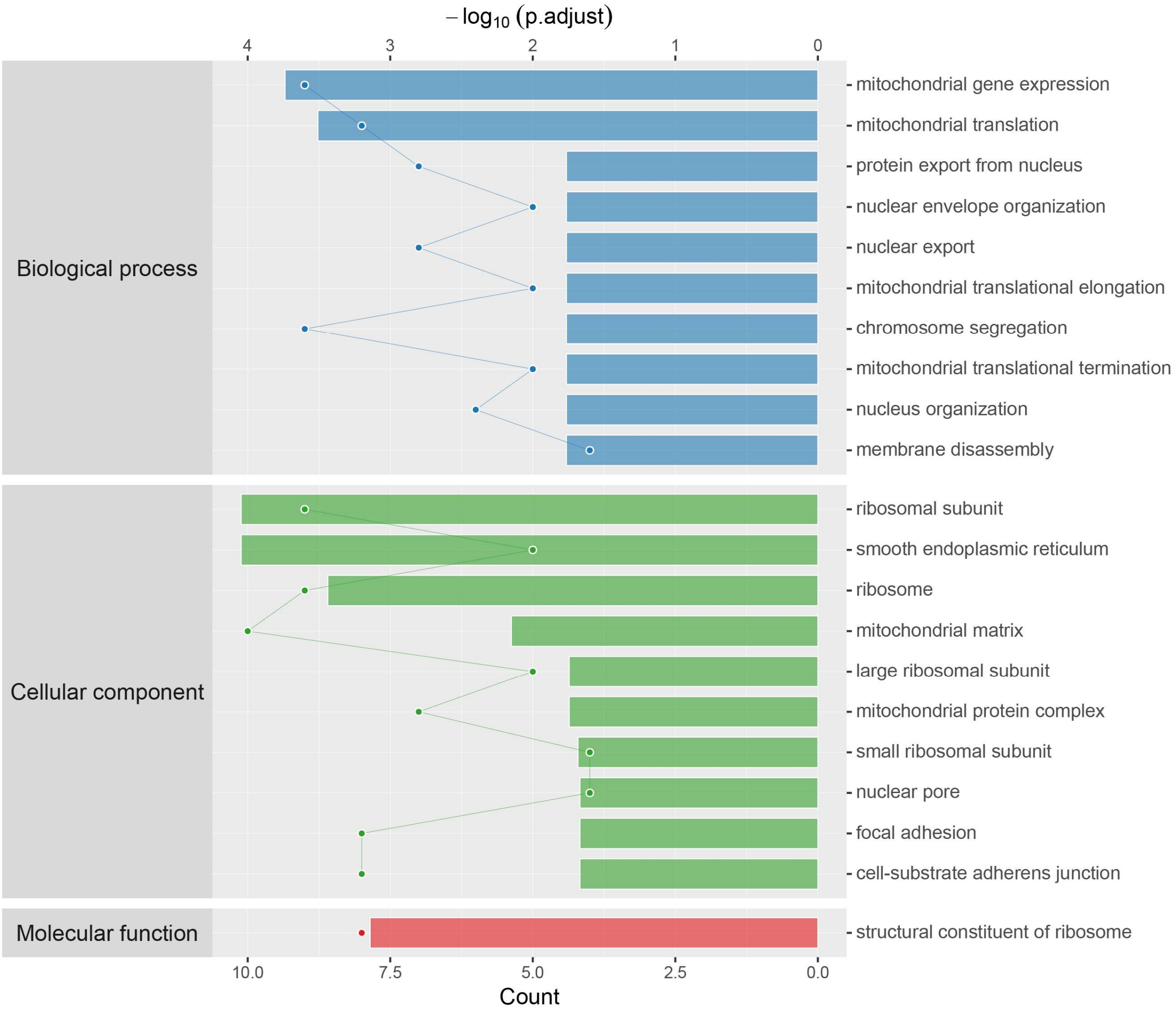

– log10 (p.adjust)
4
3
2
1
0
Biological process
Cellular component
Molecular function
mitochondrial gene expression
mitochondrial translation
protein export from nucleus
nuclear envelope organization
nuclear export
mitochondrial translational elongation
chromosome segregation
mitochondrial translational termination
nucleus organization
membrane disassembly
ribosomal subunit
smooth endoplasmic reticulum
ribosome
mitochondrial matrix
large ribosomal subunit
mitochondrial protein complex
small ribosomal subunit
nuclear pore
focal adhesion
cell-substrate adherens junction
structural constituent of ribosome
10.0
7.5
5.0
2.5
0.0
Count

Fig S7

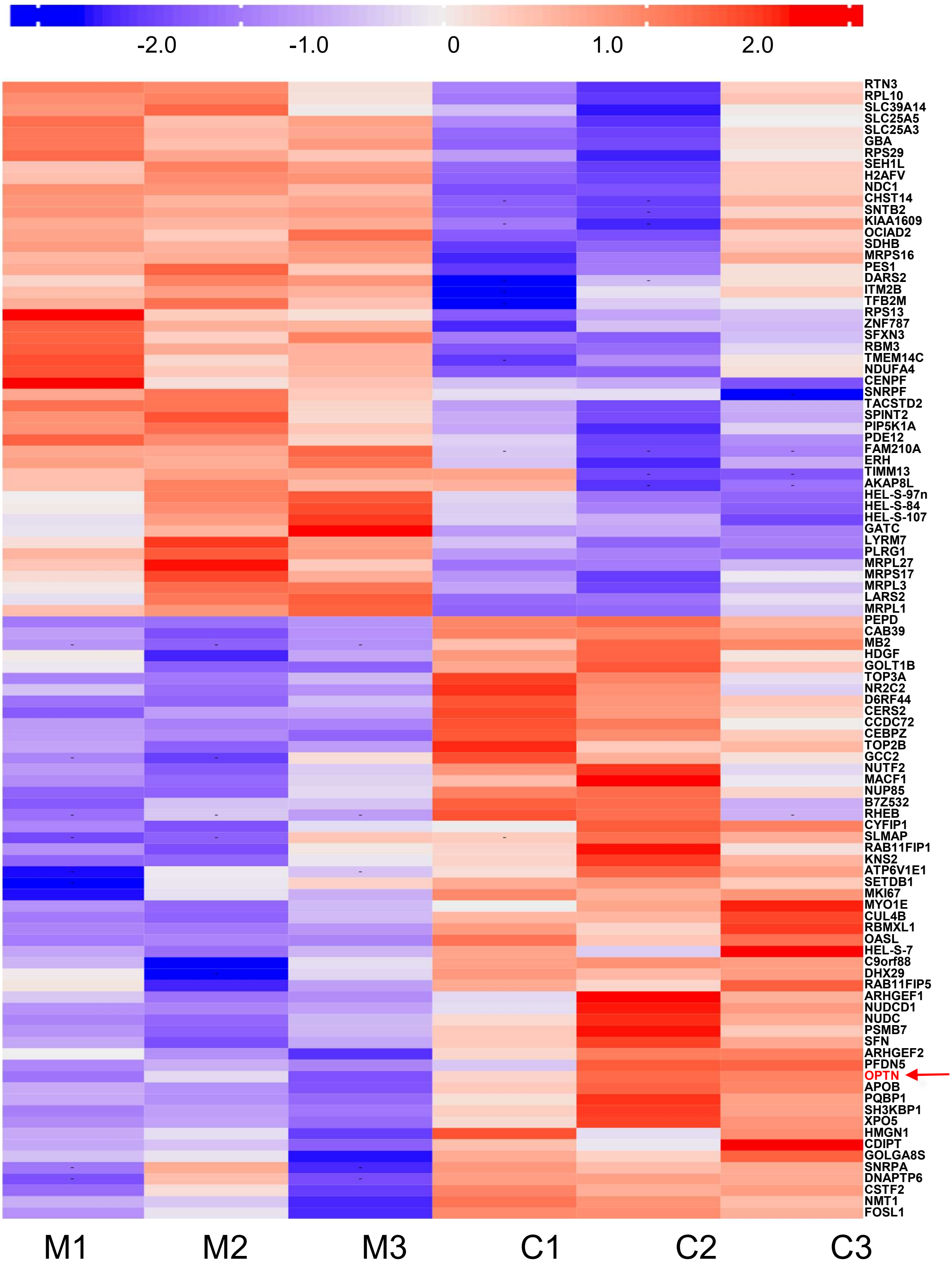

-2.0
-1.0
0
1.0
2.0
RTN3
RPL10
SLC39A14
SLC25A5
SLC25A3
GBA
RPS29
SEH1L
H2AFV
NDC1
CHST14
SNTB2
KIAA1609
OCIAD2
SDHB
MRPS16
PES1
DARS2
ITM2B
TFB2M
RPS13
ZNF787
SFXN3
RBM3
TMEM14C
NDUFA4
CENPF
SNRPF
TACSTD2
SPINT2
PIP5K1A
PDE12
FAM210A
ERH
TIMM13
AKAP8L
HEL-S-97n
HEL-S-84
HEL-S-107
GATC
LYRM7
PLRG1
MRPL27
MRPS17
MRPL3
LARS2
MRPL1
PEPD
CAB39
MB2
HDGF
GOLT1B
TOP3A
NR2C2
D6RF44
CERS2
CCDC72
CEBPZ
TOP2B
GCC2
NUTF2
MACF1
NUP85
B7Z532
RHEB
CYFIP1
SLMAP
RAB11FIP1
KNS2
ATP6V1E1
SETDB1
MKI67
MYO1E
CUL4B
RBMXL1
OASL
HEL-S-7
C9orf88
DHX29
RAB11FIP5
ARHGEF1
NUDCD1
NUDC
PSMB7
SFN
ARHGEF2
PFDN5
OPTN
APOB
PQBP1
SH3KBP1
XPO5
HMGN1
CDIPT
GOLGA8S
SNRPA
DNAPTP6
CSTF2
NMT1
FOSL1
M1
M2
M3
C1
C2
C3

Fig S8

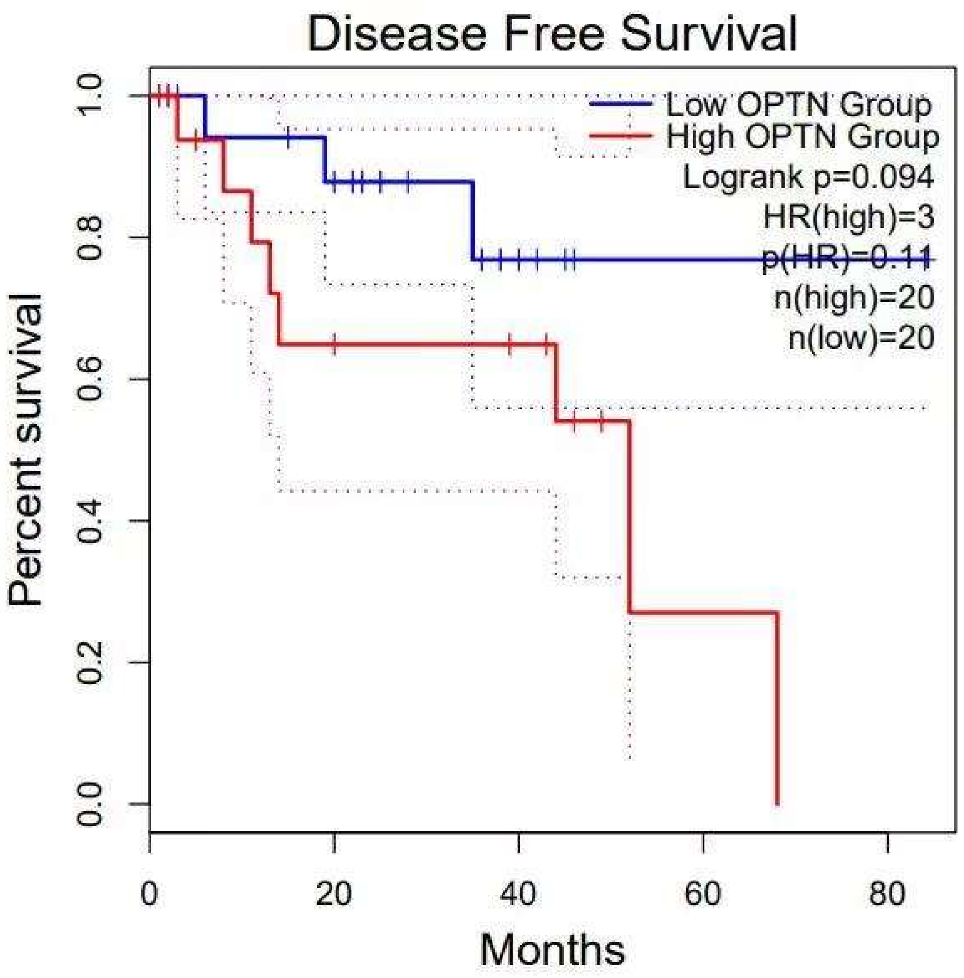
Disease Free Survival
Low OPTN Group
High OPTN Group
Logrank p=0.094
HR(high)=3
p(HR)=0.11
n(high)=20
n(low)=20
Percent survival
0.0
0.2
0.4
0.6
0.8
1.0
0
20
40
60
80
Months

Fig S9

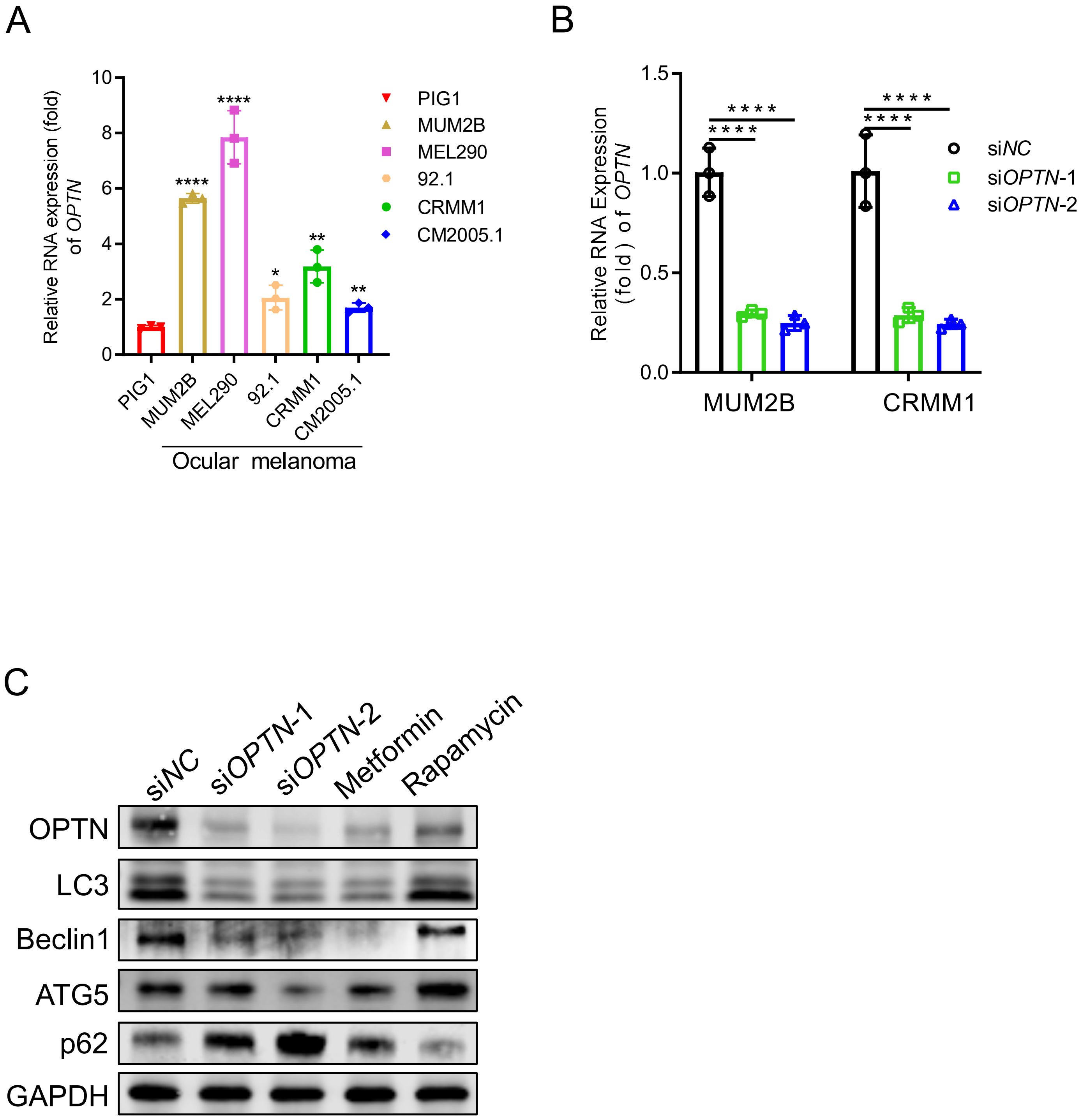
A
B
C
Relative RNA expression (fold) of OPTN
PIG1
MUM2B
MEL290
92.1
CRMM1
CM2005.1
Ocular melanoma
Relative RNA Expression (fold) of OPTN
siNC
siOPTN-1
siOPTN-2
MUM2B
CRMM1
siNC
siOPTN-1
siOPTN-2
Metformin
Rapamycin
OPTN
LC3
Beclin1
ATG5
p62
GAPDH

Fig S10

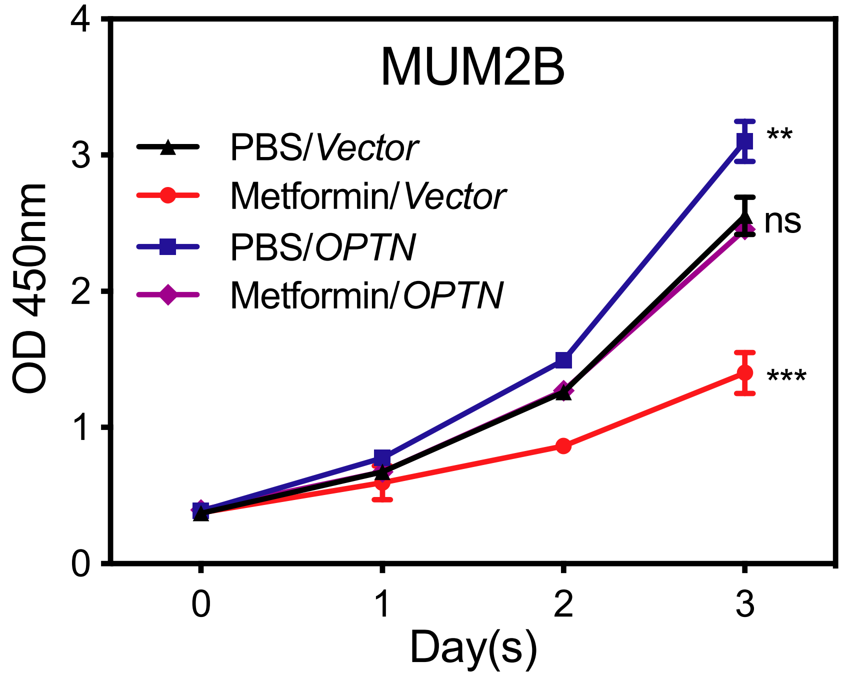
MUM2B
PBS/*Vector*
Metformin/*Vector*
PBS/*OPTN*
Metformin/*OPTN*
**
ns
***
OD 450nm
0
1
2
3
4
Day(s)

Fig S11

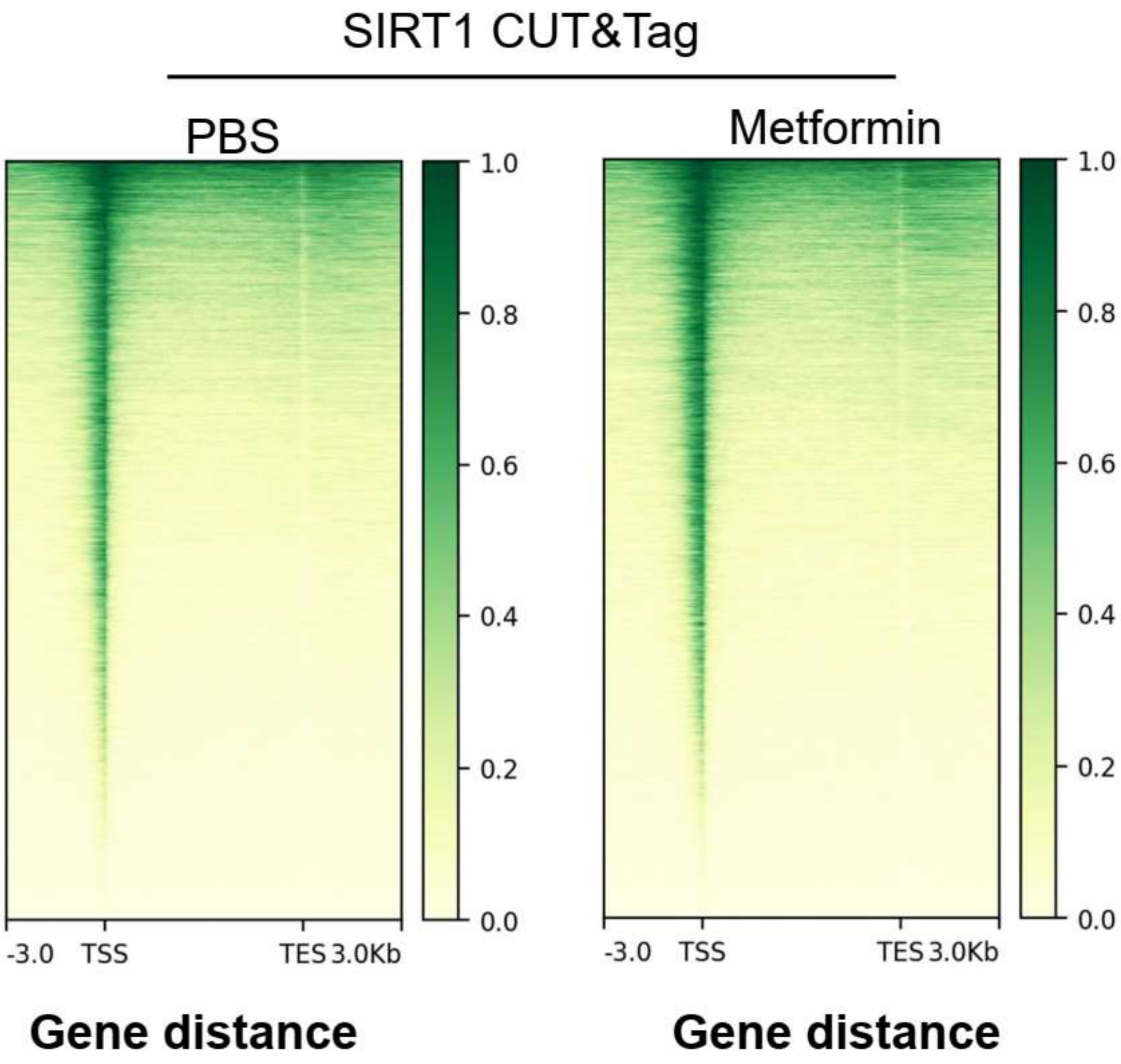
SIRT1 CUT&Tag
PBS
Metformin
1.0
0.8
0.6
0.4
0.2
0.0
-3.0
TSS
TES
3.0Kb
Gene distance

Fig S12

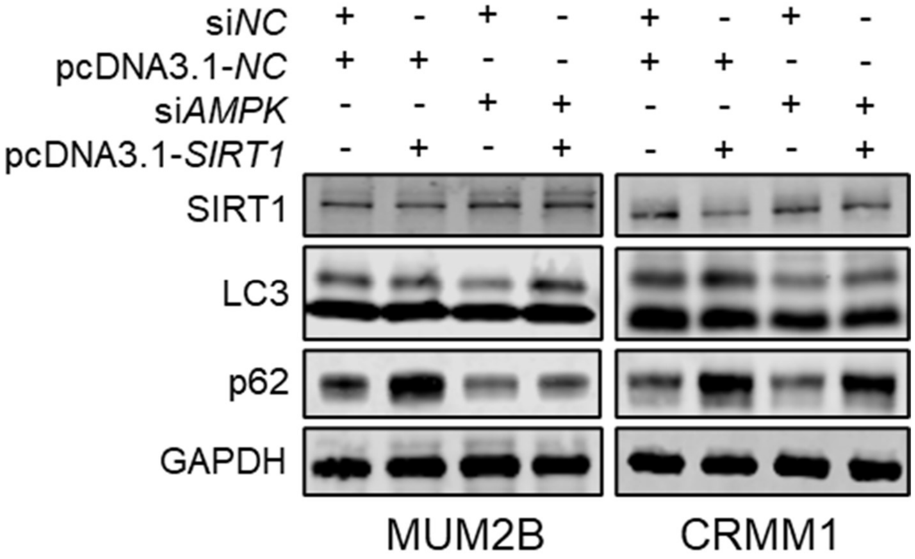

siNC
pcDNA3.1-NC
siAMPK
pcDNA3.1-SIRT1
SIRT1
LC3
p62
GAPDH
MUM2B
CRMM1

Fig S13

Figure 2A

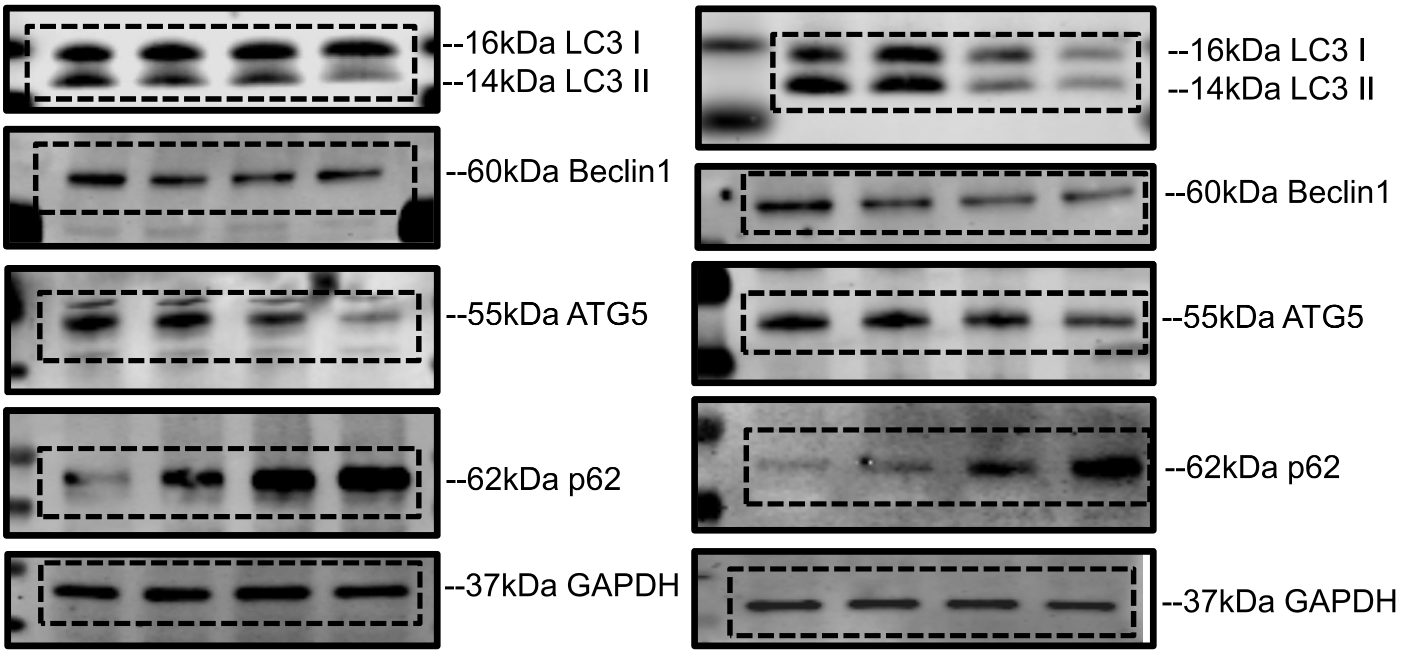

Figure 2B

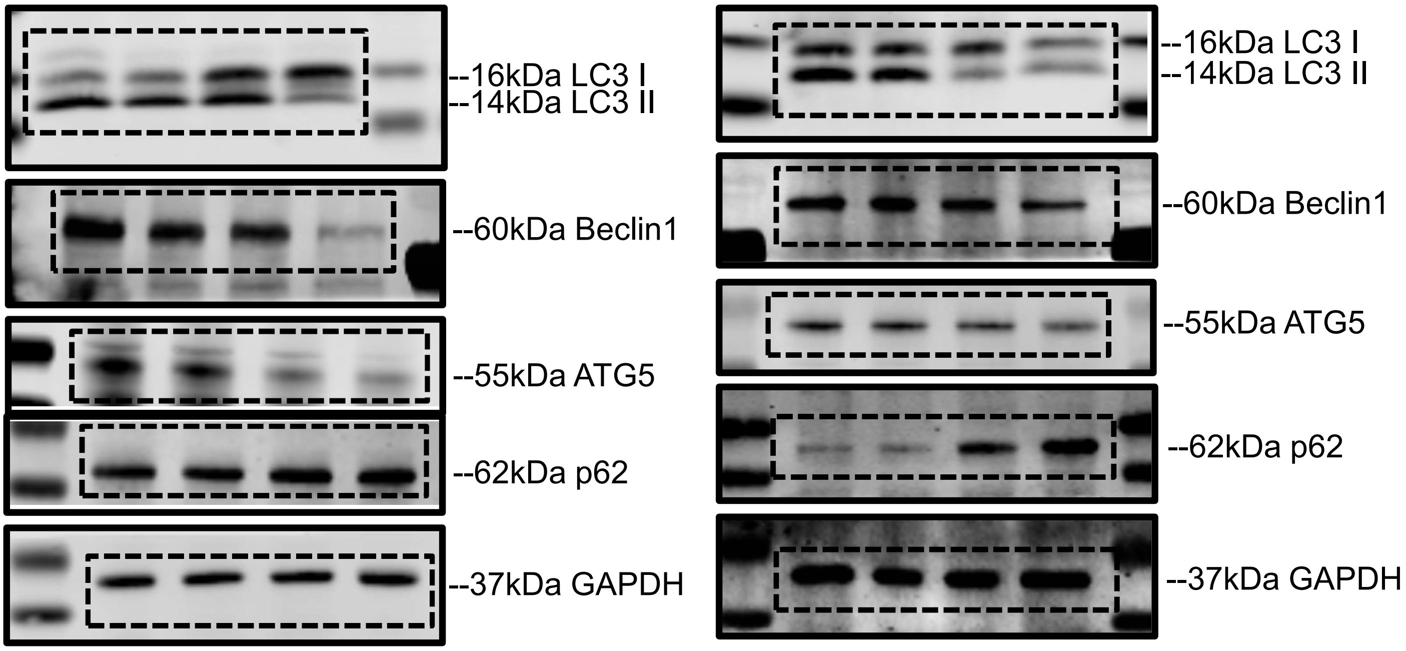

Fig S14

Figure 2D

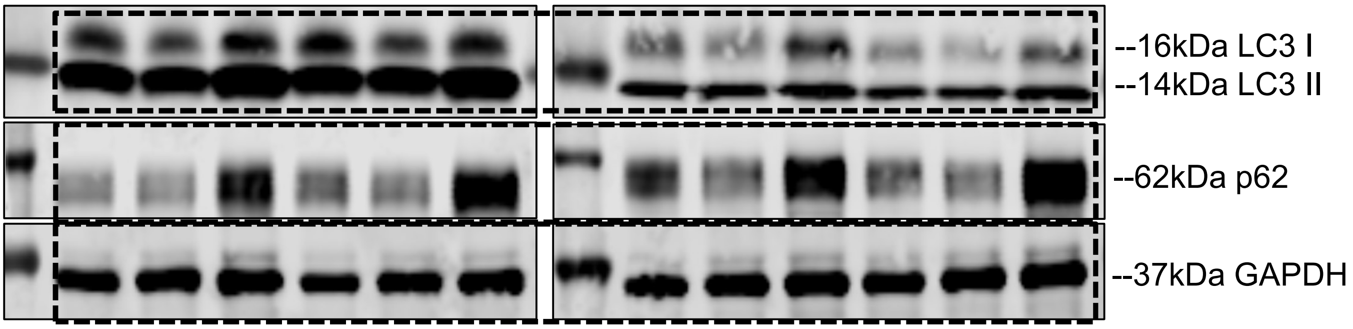

Figure 3C

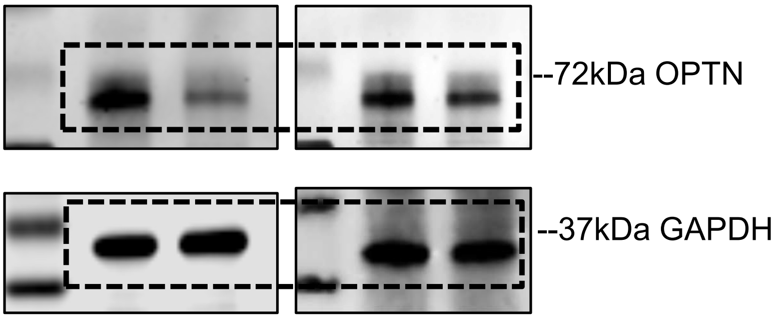

Figure 3G

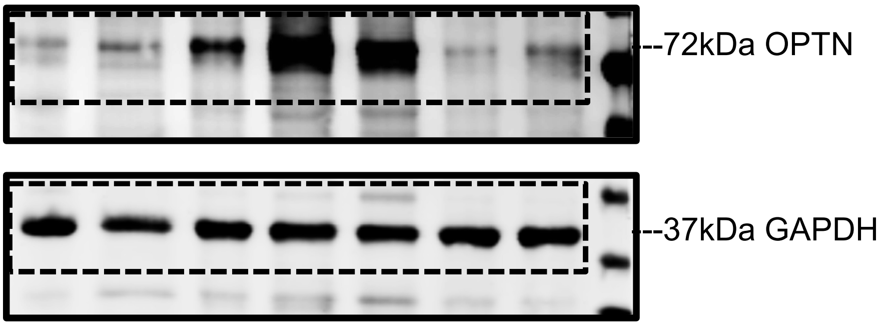

Fig S15

Figure 5B

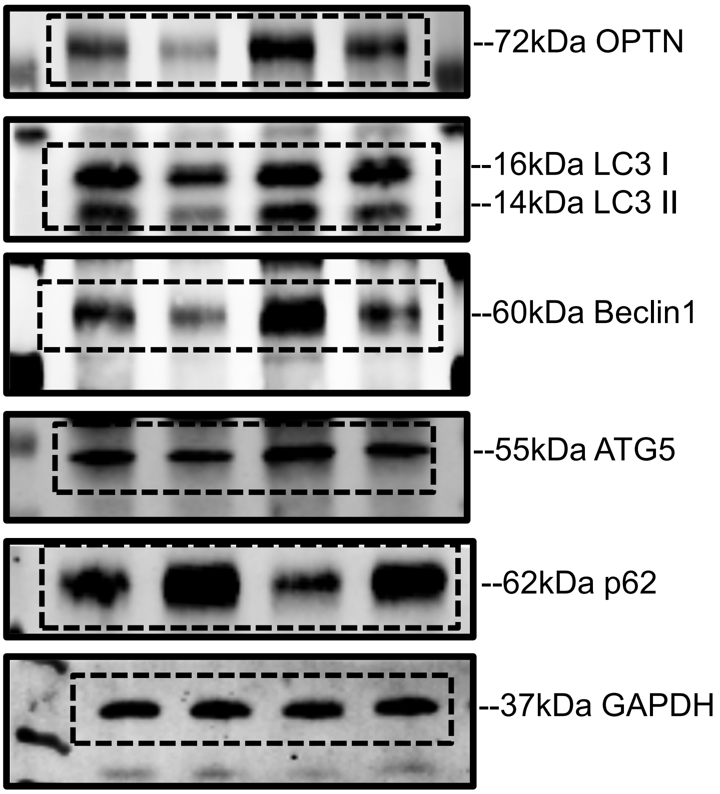

Figure 6K

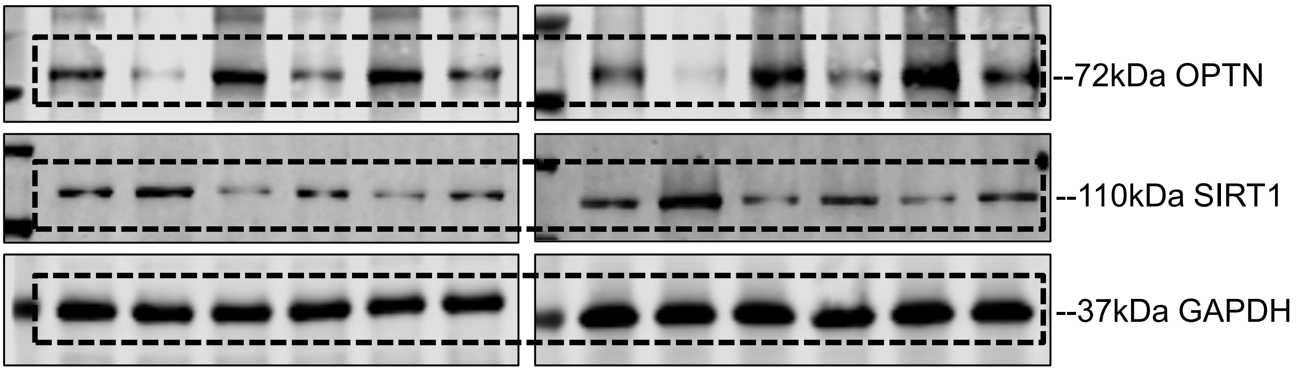

Figure 6L

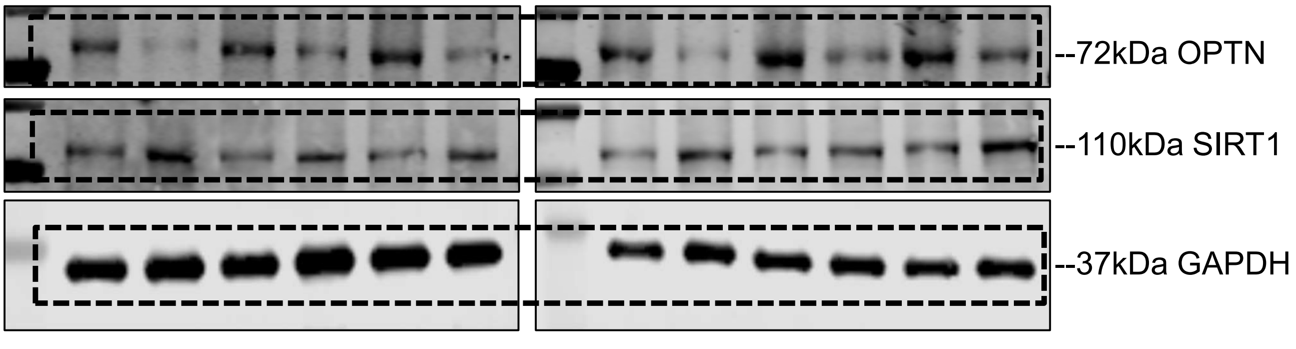

Fig S16

Figure 7A

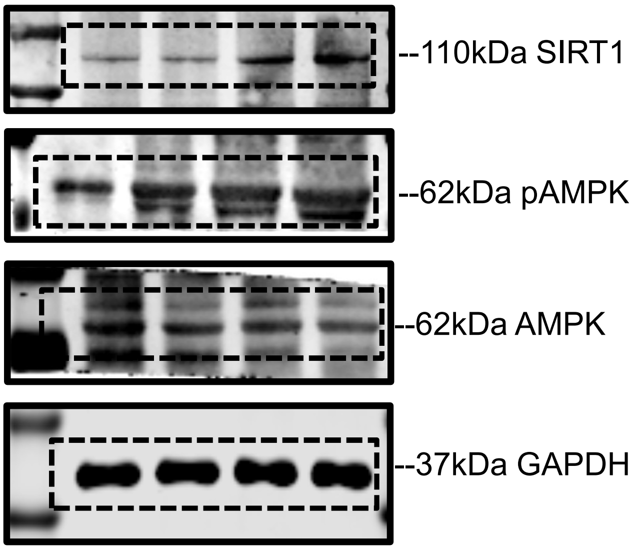

Figure 7B

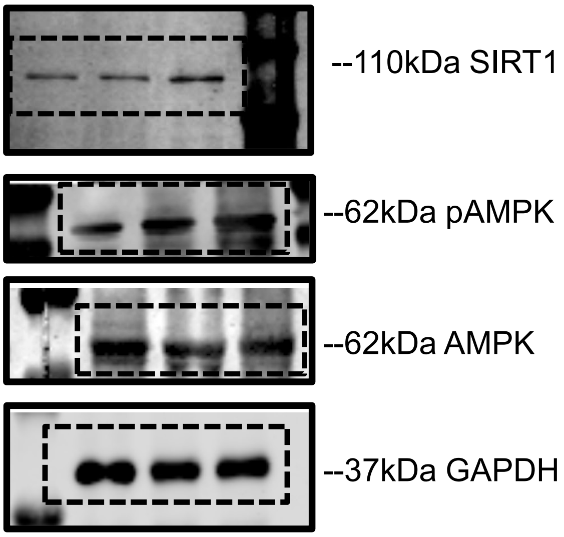

Figure 7C

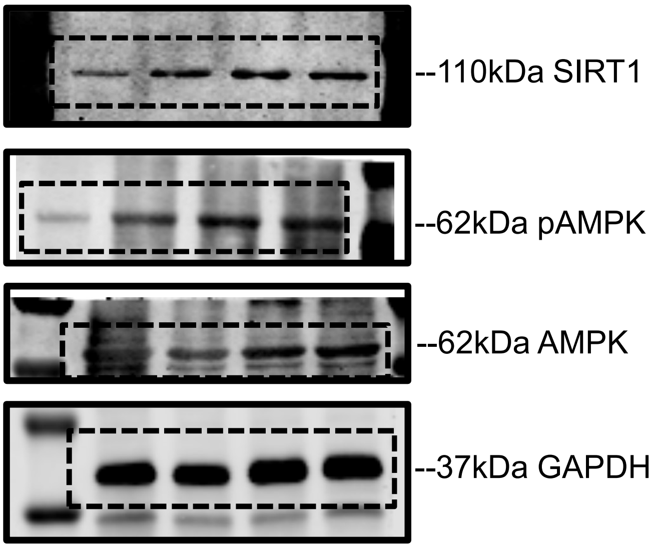

Figure 7D

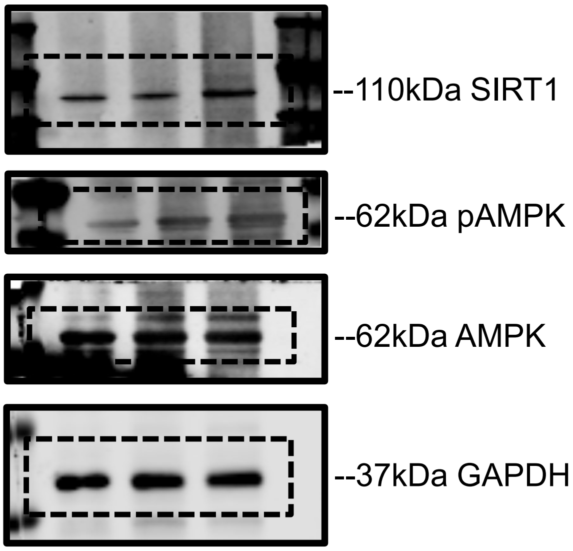

Fig S17

Figure 7E

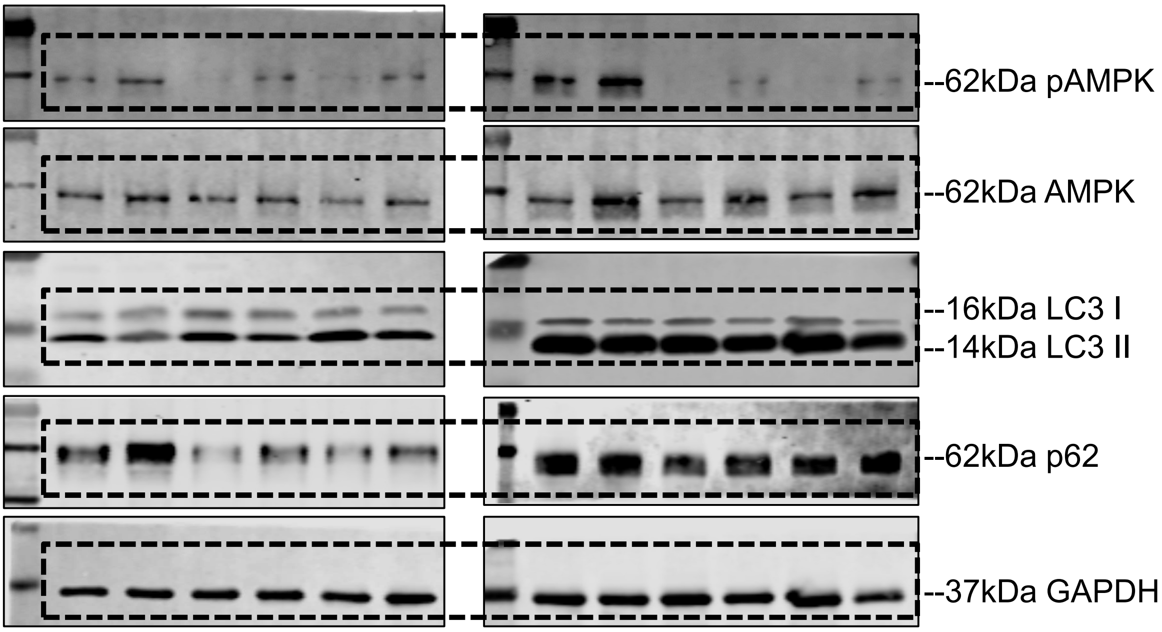

Figure 7F

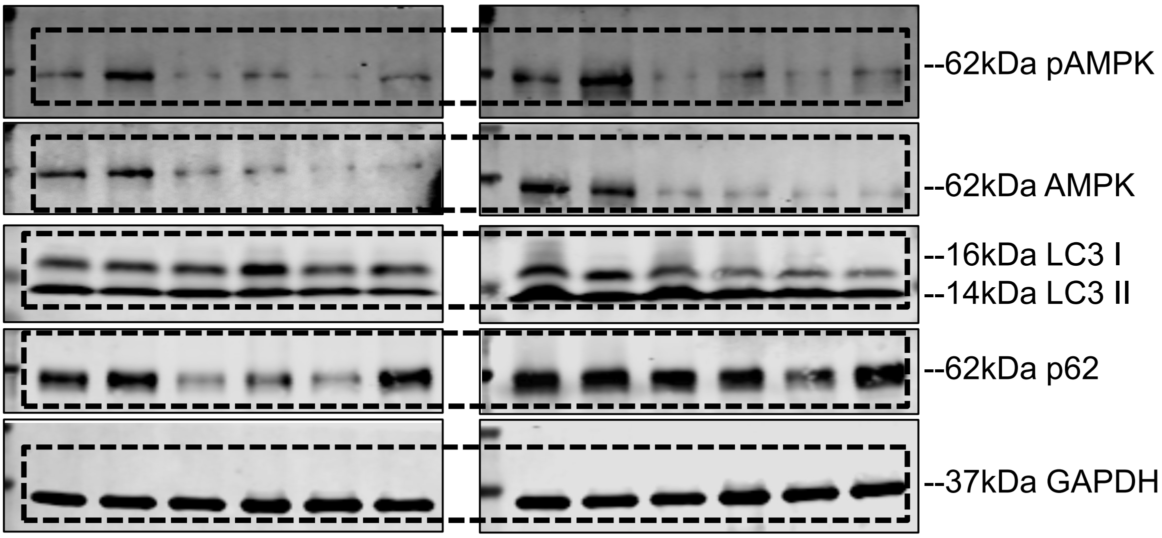

Supplement: Supplementary file 2 — Supporting Information [file CTM2-12-e660-s002.pdf]
